# Copper(II) Perfluorinated Carboxylate Complexes with Small Aliphatic Amines as Universal Precursors for Nanomaterial Fabrication

**DOI:** 10.3390/ma14237451

**Published:** 2021-12-04

**Authors:** Iwona B. Szymańska, Katarzyna Madajska, Aleksandra Butrymowicz, Magdalena Barwiołek

**Affiliations:** Faculty of Chemistry, Nicolaus Copernicus University in Toruń, Gagarina 7, 87-100 Toruń, Poland; 502533@doktorant.umk.pl (K.M.); aleksandra.butrymowicz@doktorant.umk.pl (A.B.); mbarwiolek@umk.pl (M.B.)

**Keywords:** Copper(II), ethylamine, isopropylamine, perfluorinated carboxylates, EI-MS, chemical vapor deposition, spin- and dip-coating precursors

## Abstract

Copper(II) carboxylate compounds with ethylamine and isopropylamine of the general formula [Cu_2_(RNH_2_)_2_(µ-O_2_CR_f_)_4_], where R = Et, ^i^Pr, and R_f_ = C_n_F_2n+1_, n = 1–6, were characterised in the condensed and gas phases by electron impact mass spectrometry (EI MS), IR spectroscopy, and thermal analysis. A mass spectra analysis confirmed the presence of metallated species in the gas phase. Among the observed fragments, the pseudomolecular ions [Cu_2_(RNH_2_)_2_(µ-O_2_CR_f_)_3_]^+^ were found, which suggests the dimeric structure of the studied complexes with axially N-coordinated ethyl- or isopropylamine molecules and bridging perfluorinated carboxylates. TGA studies demonstrated that copper transfer to the gas phase occurs even under atmospheric pressure. The temperature range of the [Cu_2_(RNH_2_)_2_(µ-O_2_CR_f_)_4_] and other copper carriers detection, observed in variable temperature infrared spectra, depends on the type of amine. The possible mechanisms of the decomposition of the tested compounds are proposed. The copper films were produced without additional reducing agents despite using Cu(II) CVD precursors in the chemical vapor deposition experiments. The layers of the gel-like complexes were fabricated in both spin- and dip-coating experiments, resulting in copper or copper oxide materials when heated. Dinuclear copper(II) carboxylate complexes with ethyl- and isopropylamine [Cu_2_(RNH_2_)_2_(µ-O_2_CR_f_)_4_] can be applied for the formation of metal or metal oxide materials, also in the nanoscale, by vapour and ‘wet’ deposition methods.

## 1. Introduction

Nowadays, copper compounds are applied as antifungal and antibacterial coatings, catalysts, or in the assembly of chemical and electrochemical sensors. [1]. Additionally, organic-inorganic hybrid compounds containing copper-bis(triazole) complexes and Keggin-type polyoxometalates (POMs) showed electrocatalytic activities towards the reduction of nitrites [2]. Copper-halide compounds may be employed as new light-emitting materials [3] and in photovoltaic application [4].

To the best of our knowledge, the literature data concerning Cu(II) carboxylate compounds with EtNH_2_ are limited to the dicarboxylate [Cu(EtNH_2_)_2_(C_2_O_4_)] derivative [5]. It was synthesised by adding ethylamine to a chloroform hemihydrate [Cu(C_2_O_4_)] 0.5 H_2_O solution, and its spectral, magnetic, and thermal properties were characterised. Information on copper compounds containing carboxylates and triethylamine of the following formula [Cu_2_(NEt_3_)_2_(μ-O_2_C^t^Bu)_4_] [6] and on copper polymers [Cu_2_(Et_3_NH)(syn,syn-η^1^:η^1^:μ-O_2_CR)_4_(anti,anti-η^1^:η^1^:μ-O_2_CR)]_n_, where R = Me or H and dimers such as [Cu_2_(Et_2_NH_2_)_2_(syn,syn-η^1^:η^1^:μ-O_2_CMe)_4_(anti-η^1^-O_2_CMe)_2_] and [Cu_2_(Et_3_NH)_2_(syn,syn-η^1^:η^1^:μ-O_2_CPh)_4_(anti-η^1^-O_2_CPh)_2_] can also be found in the literature [7]. Furthermore, copper(II) complexes with aromatic carboxylates with a substituent on the benzene ring (−CH_3_, −Cl, or −NO_2_) and diethylamine or dipropylamine have been previously described [8]. The possibility of *tert*-butylamine copper(II) carboxylate complexes formation by applying *tert*-butyl isocyanate was described in our previous paper [9]. Therefore, the applicability of this reaction type for the preparation of ethyl- and isopropylamine Cu(II) carboxylate compounds was a noteworthy issue. Working with isocyanates is easier due to their higher boiling points (e.g., for ethylamine. b.p = 16.6 °C so it is gaseous at room temperature, while for ethyl isocyanate, b.p = 60 °C).

The copper(II) carboxylate complexes with *tert*-butylamine were applied as Cu CVD precursors for the fabrication of copper nanomaterials fabrication. It is worth emphasizing that even with the first CVD experiments, all the [Cu_2_(^t^BuNH_2_)_2_(µ-O_2_CR_f_)_4_] (R_f_ = C_n_F_2n+1_, n = 1–6) complexes led to copper covers with no reducing agent despite those containing copper in the second oxidation state [9,10]. Over the past few years, the synthesis and research of analogous compounds have been continued and expanded. As a result, compounds with the formulas: [Cu_2_(^t^BuNH_2_)_2_(μ-O_2_CC_2_F_5_)_4_], [Cu_2_(^s^BuNH_2_)_2_(μ-O_2_CC_2_F_5_)_4_], [Cu_2_(EtNH_2_)_2_(μ-O_2_CC_2_F_5_)_4_], and [Cu_2_(EtNH_2_)_2_(μ-O_2_CC_3_F_7_)_4_] were synthesized and tested for interactions with low-energy electrons and a possible use in the focused electron beam induced deposition (FEBID) method. Due to their gel form, compounds could be directly deposited on a surface which allowed the studying them with surface science tools in a vacuum environment [11,12].

The obtained new compounds [Cu_2_(RNH_2_)_2_(µ-O_2_CR_f_)_4_], where R = Et, R_f_ = CF_3_, C_4_F_9_, C_5_F_11_, C_6_F_13_; and R = ^i^Pr, R_f_ = CF_3_, C_2_F_5_, C_3_F_7_, C_4_F_9_, form blue gels under normal conditions. Therefore, we want to examine how the N-donor ligand modification (EtNH_2_ vs. ^i^PrNH_2_) can affect the physicochemical properties of complexes, mainly their thermal stability and suitability for the generation of metallated volatile species. Due to a good solubility of the compounds, we have tested the applicability of these metal-organic compounds application in ‘wet’ coating methods. The literature data indicate that using spin- or dip- coating techniques and then heating the deposited precursors, metal or metal oxide layers at a thickness from a few tens up to several hundred nanometers can be obtained [13,14] in a low-cost process because they do not require complicated technical and hence, expensive equipment.

In the case of copper compounds, ‘wet’ deposition methods are the most often used for the copper(II) oxide materials formation. They are applied as heterogenic catalysts, in electrochemical cells for the Li_2_CuO_2_ preparation, as electrodes in photoelectrochemical PEC cells, and for the construction of gas sensors and high-temperature superconductors. Moreover, the obtained copper materials can be used for example, in electronic [15,16], plasmonics [17,18], catalysis [19,20,21], and as antibacterial agents [22,23]. In dip and spin-coating methods, systems based on copper carboxylates were applied. For example, the ethanolic solution of the [Cu_2_(H_2_O)_2_(µ-O_2_CCH_3_)_4_] complex in the presence of monoethanolamine (MEA) was used for the copper(II) oxide layer formation by the dip-coating method. The covers were obtained by heating the dip-coated films in the air at 723 K [24].

Furthermore, the anhydrous copper(II) acetate dissolved in ethanol was applied in a spin-coating process on glass substrates. The final cover (Cu_2_O) was fabricated by heating at 423 K under nitrogen. Moreover, copper carboxylate compounds were used for the metallic copper layer formation by a dip-coating method. The solution contained copper acetate dihydrate and diethanolamine (DEA) in 2-propanol. The molar ratio of DEA to Cu^2+^ was 2:1, but the concentration of the obtained sol ranged from 0.25 to 1.5 mol/dm^3^. The copper coatings were prepared by heating under nitrogen deposited on glass substrates materials [25]. The results of the complexing agents and surfactants addition aimed at improving the deposition process were described in literature while, for the compounds studied herein, the perfluorinated carboxylates as the complexing surfactants form the integral part of the molecules (*vide infra*).

In this paper, the synthesis, characteristics by electron impact mass spectrometry, infrared (IR) spectroscopy, variable temperature infrared spectroscopy (VT IR), thermal analysis, and applications of [Cu_2_(RNH_2_)_2_(µ-O_2_CR_f_)_4_] complexes, where R = Et, ^i^Pr; R_f_ = C_n_F_2n+1_, n = 1–6, in the Chemical Vapour Deposition process as well as in spin- and dip- coating methods will be described.

## 2. Experimental

### 2.1. Materials

CuCO_3_·Cu(OH)_2_, ethyl isocyanate EtNCO (97%), isopropyl isocyanate ^i^PrNCO (98%), CF_3_COOH and C_6_F_13_COOH (99%); C_3_F_7_COOH (98%); and R_f_COOH (97%) R_f_ = C_n_F_2n+1_; n = 2, 4, 5; were purchased from Sigma-Aldrich, acetonitrile (99.9%) from Honeywell, tetrahydrofuran (p.a.) from Avantor Performance Materials Poland S.A., ethanol (99.6%) from Bioetanol AEG, HF (40%) and HNO_3_ (65%) from Chempur and Si(111) from Institute of Economic Materials Technology in Warsaw. All reagents were used as received. Copper(II) carboxylates [Cu(µ-O_2_CR_f_)_2_], where R_f_ = C_n_F_2n+1_, n = 1–6, were prepared as reported [26].

### 2.2. Instrumentation

Mass spectra using a Finnigan MAT 95 mass spectrometer, Waltham, MA, USA and electron impact (EI) ionisation method were registered over the temperature range of 303–623 K. The isotopic patterns were simulated by using the shareware software Wsearch32. IR spectra were measured on a Vertex 70V spectrometer (Bruker Optik, Leipzig, Germany) using a reflective-single reflection diamond ATR unit (200–4000 cm^−1^) or using a PerkinElmer Spectrum 2000 spectrometer (Waltham, MA, USA) over 400–4000 cm^−1^ in KBr and over 200–400 cm^−1^ in PE, with a medium slit width and a peak resolution of 2.0 cm^−1^. The Cu content was determined with an iCE3300 FL atomic absorption spectrometer Thermo Fisher Scientific, Waltham, MA, USA. The content of C and H was found out with a Vario MACRO CHN ELEMENTAR Analysensysteme GmbH, Langenselbold, Germany. Thermal studies (TGA/DTA) were performed on an SDT 2960 TA analyser (New Castle, DE, USA) (dry N_2_; heating rate 2.5 K/min, heating range up to 598–692 K; sample mass 7–13 mg). Variable temperature infrared spectra (VT IR) were registered using a PerkinElmer Spectrum 2000 spectrometer (Waltham, MA, USA) over 400–4000 cm^−1^ with a medium slit width and a peak resolution of 2.0 cm^−1^. The glass vessel with the precursor sample (~100 mg) was placed in the homemade reactor tube and heated (from 303 to 753 K) under a dynamic vacuum (*p* = 10^−1^ mbar). IR spectra of vapours collected at selected temperatures were registered. Optical images of the deposited layers were made using an optical microscope RamanMicro 200 PerkinElmer (Waltham, MA, USA). The morphology and composition studies of obtained materials were performed using scanning electron microscopy: SEM-LEO 1430VP, Ltd., Cambridge, UK (operating voltage 28 kV) equipped with an energy dispersive X-ray spectrometer (EDS) Quantax 200 with detector XFlash 4010 (Bruker AXS microanalysis GmbH, Berlin, Germany). SEM images were registered using Quanta 3D FEG (FEI, Hillsboro, OR, USA). Transmission electron microscopy (TEM G2 F20X-Twin 200 kV FEI, Hillsboro, OR, USA) was used to confirm the composition of the deposited materials. To identify the chemical elements, energy-dispersive X-ray (EDX, RTEM model SN9577, 134eV, EDAX, FEI, Hillsboro, OR, USA) spectra and selected area (electron) diffraction pattern were recorded.

### 2.3. Chemical Vapour Deposition Parameters

CVD deposition experiments were conducted using the homemade horizontal hot-wall CVD reactor. Copper films were grown on Si(111) substrates in argon atmosphere within 60 min, at *p* = 10^−1^ mbar. Silicon substrate surfaces were previously cleaned according to the literature procedure [10,27]. The process parameters were selected based on the data obtained from the thermal analysis and the results of the VT IR.

### 2.4. Spin- and Dip-Coating Parameters

Precursors were dissolved either in tetrahydrofuran (THF) or acetonitrile, forming an almost saturated solution, and deposited on Si(111) substrates by the spin-coating technique using a spin-coater (Laurell 650SZ) and by the dip-coating method using a dip-coater (QPI-168 Qualtech Products Industry).

The spin coating was a multistage process, the spin speed was varied from 600 to 3000 rpm, and the process time was changed over the range 4–60 s. The SC (spin-coated) materials were obtained in four stages program. Each program consisted of three steps with variable spin speed and one drying stage (3000 rpm, 1 min). The solution introduction on the substrate and all deposition procedures for each layer were repeated twice.

The dip-coating parameters were the following: the withdraw rate 20–80 mm/min, the immersion rate 60–80 mm/min, the immersion time 5, 10, or 120 s, and the coating count 5–80. 

The films obtained by both techniques were characterised and next heated in a tube furnace PRW 55 Czylok equipped with a quartz reactor with steel gas heads. The process was carried out under a nitrogen atmosphere (1 cm^3^/min) at 673 K for 3 h. Then deposits were cooled down under nitrogen for 3 h. The heating temperature of the films was selected based on the thermal analysis results.

### 2.5. Synthesis of [Cu_2_(RNH_2_)_2_(µ-O_2_CR_f_)_4_]) (**1**–**10**)

In the general procedure, copper(II) carboxylate [Cu_2_(µ-O_2_CR_f_)_4_] (1·10^−3^ mol for R_f_ = CF_3_ (**1**) or 0.5·10^−3^ mol for R_f_ = C_4_F_9_, C_5_F_11_, C_6_F_13_ (**4**–**6**) and R = Et; 1·10^−3^ mol for R_f_ = CF_3_, C_2_F_5_, C_3_F_7_, C_4_F_9_ and R = ^i^Pr (**7**–**10**)) was dissolved in 20 cm^3^ acetonitrile, and ethyl or isopropyl isocyanate 1·10^−3^ mol or 0,5·10^−3^ mol was dropped in, respectively. The obtained reaction mixture was stirred for 4 h, on air, at room temperature for R_f_ = C_n_F_2n+1_, where n = 1–5 but at 303 K for R_f_ = C_6_F_13_ (**6**). Next, the solution was filtered, and the final product was isolated by solvent evaporation at the reduced pressure in argon. The obtained complexes were blue, gel-like solids, stable on air, which should only be moisture-protected during the storage. Yields ranged between 40–85% for R = Et and 62–72% for R = ^i^Pr. No crystals suitable for an X-ray structure determination were obtained. 

The synthesis of compounds [Cu_2_(EtNH_2_)_2_(µ-O_2_CC_2_F_5_)_4_] (**2**) and [Cu_2_(EtNH_2_)_2_(µ-O_2_CC_3_F_7_)_4_] (**3**) has been described by us earlier [11,12]. The complexes (**2**) and (**3**) were prepared and tested for interactions with low-energy electrons.

The results of elementary analyses and spectroscopic data are given below:

**[Cu_2_(EtNH_2_)_2_(µ-O_2_CCF_3_)_4_]** (**1**) C_12_H_14_Cu_2_F_12_N_2_O_8_ (calc./found) % Cu 19.0/18.3, C 21.53/21.72, H 2.11/2.28; EI-MS T = 305 K (*m/z*, RI %) [C_2_H_7_N]^+.^ (45, 45); [Cu(EtNH_2_)(O_2_CCF_3_)]^+^ (221, 11); [Cu_2_(O_2_CCF_3_)]^+^ (239, 100); [Cu_2_(EtNH_2_)(O_2_CCF_3_)]^+^ (284, 5); [Cu_2_(O_2_CCF_3_)_2_]^+.^ (352, 66); [Cu_2_(EtNH_2_)_2_(O_2_CCF_3_)_3_]^+^ (555, 3); IR (KBr, 3253, 3084, 2992, 2835, 2743, 2628, 2520, 2046, 1672, 1518, 1478, 1436, 1402, 1337, 1202, 1143, 1045, 996, 842, 797, 726, 612, 600, 520, 422; PE 426, 382, 347 cm^−1^).

Data for copper complexes (**2**) and (**3**) have been published previously [11,12]. 

**[Cu_2_(EtNH_2_)_2_(µ-O_2_CC_2_F_5_)_4_]** (**2**) C_16_H_14_Cu_2_F_20_N_2_O_8_ [12].

**[Cu_2_(EtNH_2_)_2_(µ-O_2_CC_3_F_7_)_4_]** (**3**) C_20_H_14_Cu_2_F_28_N_2_O_8_ [11].

**[Cu_2_(EtNH_2_)_2_(µ-O_2_CC_4_F_9_)_4_]** (**4**) C_24_H_14_Cu_2_F_36_N_2_O_8_ (calc./found) % Cu 10.0/10.2, C 22.71/23.03, H 1.11/1.53, EI-MS T = 345 K (*m/z*, RI %) [C_2_H_7_N]^+.^ (45,10); [Cu_2_(O_2_CC_4_F_9_)]^+^ (389, 100); [Cu_2_(EtNH_2_)(O_2_CC_4_F_9_)]^+^ (434, 1); [Cu_2_(O_2_CC_4_F_9_)_2_]^+.^ (652, 25); [Cu_2_(EtNH_2_)_2_(O_2_CC_4_F_9_)_3_]^+^ (1005, 1); IR (KBr, 3252, 3156, 3068, 2998, 2595, 2505, 1993, 1681, 1531, 1403, 1348, 1210, 1162, 1135, 1056, 1043, 1024, 914, 885, 815, 743, 714, 656, 556, 533, 452, 416; PE 421, 395, 350 cm^−1^).

**[Cu_2_(EtNH_2_)_2_(µ-O_2_CC_5_F_11_)_4_]** (**5**) C_28_H_14_Cu_2_F_44_N_2_O_8_ (calc./found) % Cu 8.7/8.2, C 22.88/22.2, H 0.96/1.10, EI-MS T = 319 K (*m/z*, RI %) [C_2_H_7_N]^+∙^ (45,28); [Cu_2_(O_2_CC_5_F_11_)]^+^ (439, 94); [Cu_2_(EtNH_2_)(O_2_CC_5_F_11_)]^+^ (484, 2); [Cu_2_(O_2_CC_5_F_11_)_2_]^+∙^ (752, 22); [Cu_2_(EtNH_2_)_2_(O_2_CC_5_F_11_)_3_]^+^ (1155, 2); IR (KBr, 3245, 3093, 2838, 2743, 2628, 2520, 2046, 1679, 1638, 1545, 1479, 1435, 1409, 1200, 1142, 1046, 996, 843, 796, 726, 612, 520; PE 427, 382, 342 cm^−1^).

**[Cu_2_(EtNH_2_)_2_(µ-O_2_CC_6_F_13_)_4_]** (**6**) C_32_H_14_Cu_2_F_52_N_2_O_8_ (calc./found) % Cu 7.6/7.7, C 23.02/22.50, H 0.85/1.13, EI-MS T = 365 K (*m/z*, RI %) ([C_2_H_7_N]^+∙^ (45, 8); [Cu_2_(O_2_CC_6_F_13_)]^+^ (489, 100); [Cu_2_(EtNH_2_)(O_2_CC_6_F_13_)]^+^ (534, 2); [Cu_2_(O_2_CC_6_F_13_)_2_]^+∙^ (852, 18); [Cu_2_(EtNH_2_)_2_(O_2_CC_6_F_13_)_3_]^+^ (1305, 1); IR (KBr, 3244, 3165, 3068, 2998, 2958, 2738, 2602, 2501, 1982, 1673, 1623, 1527, 1478, 1461, 1404, 1358, 1310, 1290, 1233, 1207, 1150, 1121, 1064, 1049, 976, 961, 930, 904, 849, 805, 788, 775, 744, 735, 718, 705, 663, 626, 591, 571, 551, 529, 459, 415; PE 429, 375, 343 cm^−1^).

**[Cu_2_(^i^PrNH_2_)_2_(µ-O_2_CCF_3_)_4_]** (**7**) C_14_H_18_Cu_2_F_12_N_2_O_8_ (calc./found) % Cu 18.21/18.42, C 24.11/24.91, H 2.60/3.51, EI-MS T = 389 K (*m/z*, RI %) [C_3_H_9_N]^+∙^ (59, 41); [Cu(^i^PrNH_2_)(O_2_CCF_3_)]^+^ (235, 28); [Cu_2_(^i^PrNH_2_)(O_2_CCF_3_)]^+^ (298, 14); [Cu_2_(O_2_CCF_3_)_2_]^+∙^ (352, 63); [Cu_2_(^i^PrNH_2_)_2_(O_2_CCF_3_)_3_]^+^ (583, 77); IR (ATR, 3082, 2983, 2947, 1666, 1520, 1471, 1431, 1400, 1385, 1190, 1136, 1024, 937, 922, 841, 795, 725, 613, 521, 469, 420, 375, 347 cm^−1^).

**[Cu_2_(^i^PrNH_2_)_2_(µ-O_2_CC_2_F_5_)_4_]** (**8**) C_18_H_18_Cu_2_F_20_N_2_O_8_ (calc./found) % Cu 14.15/14.84, C 24.09/23.90, H 2.02/2.39, EI-MS T = 404 K (*m/z*, RI %) [C_3_H_9_N]^+∙^ (59, 46); [Cu(^i^PrNH_2_)(O_2_CC_2_F_5_)]^+^ (285, 24); [Cu_2_(O_2_CC_2_F_5_)]^+^ (289, 3); [Cu_2_(^i^PrNH_2_)(O_2_CC_2_F_5_)]^+^ (348, 16); [Cu_2_(^i^PrNH_2_)_2_(O_2_CC_2_F_5_)_3_]^+^ (733, 72); IR (ATR, 3065, 2986, 1675, 1523, 1471, 1403, 1323, 1207, 1158, 1028, 818, 732, 586, 542, 471, 420, 377, 360 cm^−1^).

**[Cu_2_(^i^PrNH_2_)_2_(µ-O_2_CC_3_F_7_)_4_]** (**9**) C_22_H_18_Cu_2_F_28_N_2_O_8_ (calc./found) % Cu 11.57/14.09, C 24.08/24.09, H 1.65/2.02, EI-MS T = 372 K (*m/z*, RI %) [C_3_H_9_N]^+∙^ (59, 16); [Cu(^i^PrNH_2_)(O_2_CC_3_F_7_)]^+^ (334, 4); [Cu_2_(O_2_CC_3_F_7_)]^+^ (339, 24); [Cu_2_(^i^PrNH_2_)(O_2_CC_3_F_7_)]^+^ (398, 5); [Cu_2_(O_2_CC_3_F_7_)_2_]^+∙^ (552, 4); [Cu_2_(^i^PrNH_2_)_2_(O_2_CC_3_F_7_)_3_]^+^ (883, 24); IR (ATR, 3088, 2990, 2955, 1683, 1630, 1592, 1522, 1473, 1406, 1340, 1275, 1207, 1165, 1119, 1086, 967, 934, 818, 762, 745, 722, 645, 603, 589, 528, 475, 428, 376, 351 cm^−1^).

**[Cu_2_(^i^PrNH_2_)_2_(µ-O_2_CC_4_F_9_)_4_]** (**10**) C_26_H_18_Cu_2_F_36_N_2_O_8_ (calc./found) % Cu 9.79/10.32, C 24.07/23.39, H 1.40/2.23, EI-MS T = 401 K (*m/z*, RI %) [C_3_H_9_N]^+∙^ (59, 23); [Cu(^i^PrNH_2_)(O_2_CC_4_F_9_)]^+^ (385, 3); [Cu_2_(O_2_CC_4_F_9_)]^+^ (389, 64); [Cu_2_(^i^PrNH_2_)(O_2_CC_4_F_9_)]^+^ (448, 5); [Cu_2_(O_2_CC_4_F_9_)_2_]^+∙^ (652, 9); [Cu_2_(^i^PrNH_2_)_2_(O_2_CC_4_F_9_)_3_]^+^ (1033, 21), IR (ATR, 3095, 2987, 1681, 1627, 1588, 1522, 1475, 1403, 1347, 1198, 1163, 1130, 1060, 1023, 939, 916, 888, 813, 743, 717, 657, 603, 554, 535, 470, 419, 376, 352 cm^−1^).

## 3. Results and Discussion

In the case of other simple aliphatic amines, the following reaction:

2 [Cu(µ-O_2_CR_f_)_2_] + 2 RNCO + 2H_2_O → [Cu_2_(RNH_2_)_2_(µ-O_2_CR_f_)_4_] + 2CO_2_ is analogous to those we reported copper compounds containing tert-butylamine [9], as well as ethylamine [Cu_2_(EtNH_2_)_2_(µ-O_2_CC_2_F_5_)_4_] (**2**) and [Cu_2_(EtNH_2_)_2_(µ-O_2_CC_3_F_7_)_4_] (**3**) complexes [11,12].

The obtained compounds (**1**–**10**) were blue, gel-like solids, stable without moisture access for months. 

### 3.1. Infrared Spectra Analysis

The results of infrared spectroscopy are given in (Table 1, Figures S1–S10) The strong absorption bands typical of stretching vibrations of the isocyanate group ν_as_N=C=O = 2279 cm^−1^ for ethyl isocyanate and ν_as_N=C=O = 2267 cm^−1^ for isopropyl isocyanate disappeared. Simultaneously, the broad and intense band of the amine N-H stretching and hydrogen bonding vibrations were detected over the range 3060–3100 cm^−1^ (Table 1). The coordination shifts of this band were calculated in relation to the free amine (for ethylamine—3360 cm^−1^, isopropylamine—3380 cm^−1^), and they achieved the values approximately 300–270 cm^−1^ (**1**–**6**) and 320–280 cm^−1^ (**7**−**10**), respectively. The Cu–N bond formation and the occurrence of hydrogen bonds NH–F influenced the high value of this shift [28]. The shift of the N-H stretching band towards the lower wavenumber was also observed for other amine complexes of copper as well as palladium and silver [29,30,31]. Additionally, the signals over the range 1520–1545 cm^−1^ were detected. They can be assigned to the scissors deformation vibration of the amine group. These bands were shifted down for about 102–80 cm^−1^ (**1**−**6**) and 98–95 cm^−1^ (**7**−**10**), which confirms the ethyl- and isopropylamine coordination. The shift of the stretching and deformation bands changed irregularly with the length of the carboxylate carbon chain. 

Asymmetric and symmetric stretching vibrations of the carboxylic group were detected over the ranges: ν_as_COO = 1673–1681 cm^−1^ and ν_s_COO = 1403–1436 cm^−1^ (Table 1, Figures S1–S10). The calculated ∆ν = ν_as_COO − ν_s_COO parameter for the [Cu_2_(EtNH_2_)_2_(µ-O_2_CCF_3_)_4_] (**1**) amounted ∆ν = 236 cm^−1^ and in the case of [Cu_2_(EtNH_2_)_2_(µ-O_2_CC_n_F_2n+1_)_4_], n = 2–6 (**2**–**6**) achieved ∆ν = 262–278 cm^−1^. While for compounds containing an isopropyl group, the calculated amounted ∆ν = 235 cm^−1^ for the [Cu_2_(^i^PrNH_2_)_2_(µ-O_2_CCF_3_)_4_] (**7**) and reached the value of ∆ν = 272–278 cm^−1^ for the [Cu_2_(^i^PrNH_2_)_2_(µ-O_2_CC_n_F_2n+1_)_4_], n = 2–4 (**8**–**10**). These values are close to the same parameter found for the appropriate sodium salts (Δν(CF_3_COONa) = 223 cm^−1^ and Δν(C_n_F_2n+1_COONa; n = 2–6) = 268–272 cm^−1^) which suggests the bridging coordination mode of carboxylates. The analogous relationship was observed in the case previously described [Cu_2_(^t^BuNH_2_)_2_(µ-O_2_CC_n_F_2n+1_)_4_] complexes with *tert*-butylamine [9]. The bridging mode of copper(II) carboxylates is in favor of complexes’ dimeric units in the solid state [32,33].

Assuming the C_4v_ microsymmetry of the Cu(II) coordination sphere [9] spectra in the metal-ligand vibrations region (600–100 cm^−1^) can be described in the following manner: the bands detected over the range 419–429 cm^−1^ can be assigned to Cu–N stretching vibrations and these observed over 375–395 cm^−1^ and 331–360 cm^−1^—to asymmetric and symmetric CuO_4_ stretching vibrations, respectively [28].

### 3.2. Mass Spectra Analysis

Mass spectra (EI MS) of the studied compounds were measured between 303 and 623 K (Figures S11–S20) and applied for the molecular mass determination and structural characteristics. Additionally, metallated fragments in the gas phase can be identified, and a preliminary usefulness evaluation of usefulness by vapour deposition techniques can also be considered. 

For the [Cu_2_(EtNH_2_)_2_(µ-O_2_CR_f_)_4_] complexes (**1**–**6**) (Table 2 and Tables S1–S5), the following fragments, characteristic of ethylamine, were detected: [C_2_H_7_N]^+^ (45 *m/z*), [C_2_H_4_N]^+^ (42 *m/z*), [C_2_H_3_N]^+^ (41 *m/z*), [C_2_H_2_N]^+^ (40 *m/z*). Among these ions, the [C_2_H_7_N]^+^ fragment exhibits RI = 69% for (**1**) (Table 2) at 355 K. Other complexes (**2**–**6**) revealed a different behaviour, and their RI achieved only 10–38% over the temperature range 305–357 K. Additionally, the copper-amine ion [Cu(EtNH_2_)]^+^ (108 *m/z*) was observed for the following complexes: (**2**) (3–4% RI, up to 357 K), (**3**) (2–3% RI, up to 463 K), and (**6**) (1–2% RI, up to 431 K).

Similar ions were observed for the [Cu_2_(^i^PrNH_2_)_2_(µ-O_2_CR_f_)_4_] complexes (**7**–**10**) (Table 3 and Tables S6–S8), a molecular ion characteristic for isopropylamine was recorded [C_3_H_9_N]^+^ (59 *m/z*) and achieved the highest intensity RI = 46% for (**8**) at 404 K, for other compounds intensity of this ion were in the range of 16–41% for (**7, 9, 10**) at 372–389 K. The highest intensity of a metallated fragment with isopropylamine molecule [Cu(^i^PrNH_2_)]^+^ (122 *m/z*) were observed for (**8**) RI = 53% at 404 K, while for other compounds (**7**) RI = 40% at 389 K and (**9**) RI = 25% at 372 K.

**Figure 1:**
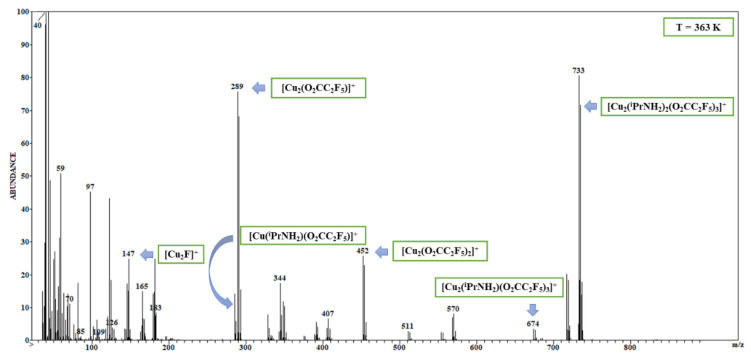
EI MS spectrum of [Cu_2_(^i^PrNH_2_)_2_(µ-O_2_CC_2_F_5_)_4_] (**8**), T = 363 K.

**Figure 2:**
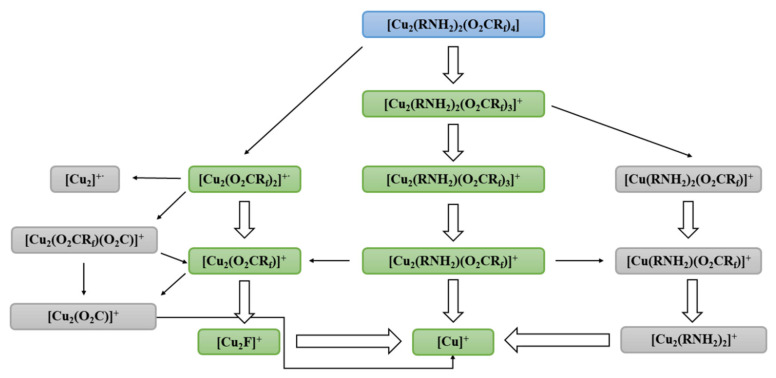
Fragmentation scheme for metallated fragments of [Cu_2_(RNH_2_)_2_(μ-O_2_CR_f_)_4_] (marked green—observed for all compounds (**1**–**10**); marked grey—observed for some complexes).

The [Cu_2_(O_2_CR_f_)]^+^ and [Cu_2_(O_2_CR_f_)_2_]^+·^ dicopper carboxylate ions were noticed in the gas phase of all the studied complexes (Figure 1 and Figure 2), which confirms the dimeric structure of the compounds (**1**–**10**). For the complexes with ethylamine, the [Cu_2_(O_2_CR_f_)]^+^ ion achieved the highest intensity (up to 100%) over the range 305–425 K (Table 2 and Tables S1–S5). Interestingly, for the complexes (**2**) and (**3**), the high intensity (100% RI) of the [Cu_2_(O_2_CR_f_)]^+^ ion was maintained up to 542 K (Tables S1 and S2). For compounds with isopropylamine, the [Cu_2_(O_2_CR_f_)]^+^ ion reaches a maximum intensity of 64% RI at 401 K for compound (**10**). The [Cu_2_(O_2_CR_f_)_2_]^+·^ fragment generally accompanies the [Cu_2_(O_2_CR_f_)]^+^ ion, but its intensity is usually lower. The fragmentation of the long-chain carboxylates influences the amount and intensity of the fluorocarbon species, e.g., [C_2_F_5_]^+^, [C_4_F_7_]^+^. The formation of the [C_3_F_5_]^+^ ion (RI to 100%), which is the analogue of the allyl cation, was observed for the compounds (**3**–**6** and **9**–**10**). Moreover, the less complicated metallated fragments: [Cu_2_F]^+^ (145 *m/z*), [Cu_2_]^+·^ (126 *m/z*), and [Cu]^+^ (63 *m/z*) were detected as the fragmentation terminal products. This fact may indicate the sensitivity of the compounds to the electron beam and the formation of a mixture of copper and copper(I) fluoride. The intensity of the [Cu_2_F]^+^ ion was relatively high comparison to the analogous complexes with *tert*-butylamine [9]. The [Cu_3_(O_2_CR_f_)_5_]^+^ ion was also detected for some studied carboxylate compounds with ethylamine (**1**, **4**, **5**) and isopropylamine (**9, 10**).

The detection of the metallated fragments containing both ligands, i.e., [Cu(RNH_2_)(O_2_CR_f_)]^+^, [Cu(RNH_2_)_2_(O_2_CR_f_)]^+^, [Cu_2_(RNH_2_)(O_2_CR_f_)]^+^, [Cu_2_(RNH_2_)(O_2_CR_f_)_3_]^+^ and the pseudomolecular [Cu_2_(RNH_2_)_2_(O_2_CR_f_)_3_]^+^ ion (simulations of isotopic patterns are shown in the Figures S11–S20) was the most important (Figure 1 and Figure 2). The latter was formed by one carboxylate ligand detaching. It was also observed for the complex (**6**) whose molecular mass is the highest. The majority of metallated fragments were dinuclear, but in the case of the complexes (**1**), (**7**)–(**10**), monocopper fragments containing both ligands were detected as well (Table 2 and Tables S6–S8). In the mass spectra of the tested compounds, the following fragments which contain Cu(I) ions appear: [Cu(RNH_2_)]^+^, [Cu_2_(O_2_CR_f_)]^+^, [Cu_2_(O_2_CR_f_)_2_]^+·^, and [Cu_2_(RNH_2_)(O_2_CR_f_)]^+^. It suggests that the copper(II) reduction can occur during the thermolysis. When the [Cu_2_(^t^BuNH_2_)_2_(µ-O_2_CR_f_)_4_] complexes were used as Cu CVD precursors, this process resulted in metallic copper formation [10]. Generally, the metallated fragments group achieved the highest intensity (at least the level of a few percent) over the temperature range 305–600 K and 340−548 K for complexes with ethyl- and isopropylamine, respectively. The complexes (**1**), (**2**), and (**5**) showed the lowest temperature of the metallated fragments generation, and they seemed to be promising for CVD purposes.

Summarising, the types of the observed fragments in the case of ethyl- and isopropylamine complexes were almost the same as those of the *tert*-butylamine derivatives. Additionally, the [Cu(O_2_C)]^+^ ion (**2, 3, 6, 8**) and the [Cu(RNH_2_)_2_(O_2_CCF_3_)]^+^ fragment (**1, 7**–**10**) were detected. The temperatures at which the [Cu_2_(EtNH_2_)_2_(O_2_CR_f_)_3_]^+^ pseudomolecular ions achieved the highest intensity, were generally lower (305–357 K) for the [Cu_2_(EtNH_2_)_2_(µ-O_2_CR_f_)_4_] complexes than in the case of the [Cu_2_(^i^PrNH_2_)_2_(µ-O_2_CR_f_)_4_] (389−404 K) and [Cu_2_(^t^BuNH_2_)_2_(µ-O_2_CR_f_)_4_] analogues (369–465 K). It means that the complexes with ethylamine show the highest volatility among the tested compounds. A similar dependence was observed for the [Cu_2_(O_2_CR_f_)]^+^ ions. The highest intensities (up to 100%) were achieved over the temperature range 305–425 K (EtNH_2_) in relation to 401–548 K (^i^PrNH_2_) and 505–573 K (^t^BuNH_2_). Seemingly, these data concerning temperatures may appear as promising for the chemical vapour deposition purposes. Additionally, the [Cu]^+^ high intensity (40% RI), especially observed simultaneously with the pseudomolecular ion detection, suggests that, from among the studied complexes with EtNH_2_, ^i^PrNH_2_, and ^t^BuNH_2_, to the electron, is [Cu_2_(EtNH_2_)_2_(µ-O_2_CCF_3_)_4_] is the compound most sensitive to the electrons (**1**).

### 3.3. Results of Thermal Analysis

The thermal analysis of the complexes (**1**–**4**) and (**7**–**10**) indicates that the slow weight loss occurs from the beginning of heating of the complex (Figures S21 and S23–S28). A complicated process is observed at higher temperatures. Analysis of the DTA curve for these compounds indicates the endothermic effects connected with thermal decomposition.

The temperature of the decomposition process onset changed over the range 388–410 K and 308–355 K for complexes containing ethyl or isopropyl group, respectively. The temperature of the final product formation varied from 505 K to 532 K (**1**), (**3**), (**4**), and 478 K to 548 K (**7**–**10**). The lowest T_m_ (466 K and 463–464 K) was observed for the compound (**3**), (**9**), and (**10**) containing chain R_f_ = C_3_F_7_, C_4_F_9_ and the highest value (486 K and 474 K) for (**1**) and (**7**) including group CF_3_. The mass of the residues after the thermal analysis for compounds (**1**) and (**7**) reveals that the decomposition product of the compounds with EtNH_2_ is metallic copper, while for compounds with ^i^PrNH_2,_ it is copper(II) oxide (Table 4). The formation of these substances is also confirmed by the color of the residue, red-orange and black, respectively. In the case of the complexes (**3**), (**4**), (**8**), and (**9**), the mass of the final decomposition products was lower than the value calculated for pure copper or copper(II) oxide (Table 4), which suggests that the copper transfer to the gas phase occurred under the atmospheric pressure, which seemed promising for the CVD and FEBID application. The higher residue mass with regard to its theoretical value for the compound (**10**) (R_f_ = C_6_F_13_) may indicate carbon contamination during thermal decomposition.

In the case of [Cu_2_(EtNH_2_)_2_(µ-O_2_CR_f_)_4_] complexes, the onset temperatures achieved higher values than those for the *tert*-butylamine analogues. However, for the [Cu_2_(^i^PrNH_2_)_2_(µ-O_2_CR_f_)_4_] complexes, the initial temperatures of the decomposition process were similar to the temperatures observed for the compounds containing the *tert*-butylamine group. On the contrary, the observed final decomposition temperatures were lower for the compounds containing ethylamine and also the isopropylamine group. It means that the thermal decomposition ranges for the [Cu_2_(RNH_2_)_2_(µ-O_2_CR_f_)_4_] complexes discussed here are narrower than those for previously described [Cu_2_(^t^BuNH_2_)_2_(µ-O_2_CR_f_)_4_] compounds. Interestingly, in the case of complexes with ^t^BuNH_2_, [Cu_2_(^t^BuNH_2_)_2_(µ-O_2_CCF_3_)_4_] was the most volatile, while for ^i^PrNH_2_ and EtNH_2,_ it was observed that compounds with trifluoroacetate have the lowest volatility [9].

**Figure 3:**
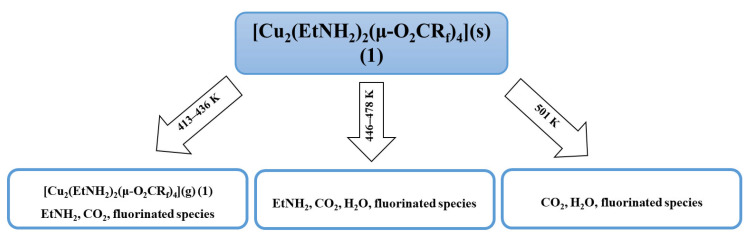
The thermal decomposition scheme for [Cu_2_(EtNH_2_)_2_(μ-O_2_CCF_3_)_4_] (**1**) proposed based on TG-IR results.

Due to the complexity of the process of thermal decomposition of the compounds, we decided to investigate it in more detail for the complex (**1**) by examining gas phase composition during thermal analysis by infrared spectroscopy (TGA/IR). The spectra analysis showed the characteristic absorption bands for coordinated ethylamine (νCH = 2990 cm^−1^, δNH_2_ = 1524 cm^−1^) and carboxylate ligands (ν_as_COO = 1701 cm^−1^, ν_s_COO = 1440 cm^−1^). These results suggested that from 413 to 436 K, the complex (**1**) undergoes evaporation. The 2361 cm^−1^ band, characteristics of CO_2_ [34], was detected over the whole studied temperature range (413–501 K), but between 446 and 478 K, its intensity decreased rapidly (Figure S22). The bands typical for fluorinated species (νCF = 1199 cm^−1^, 1156 cm^−1^) were observed throughout the temperature range. The typical bands for H_2_O (νOH = 4000–3500 cm^−1^, δOH = 1800–1300 cm^−1^) [35] were registered over the 446–501 K. The free amine [36] was detected over 413–478 K. The observed organic molecule bands indicate the partial compound decomposition in the gas phase (Figure 3). 

The TGA/IR results confirm that the thermal decomposition of the compound (**1**) is a complicated process. Additionally, it has been shown that the complex (**1**) enters the gas phase in the initial heating phase in a narrow temperature range and is accompanied by its decomposition.

### 3.4. Temperature Variable Infrared Spectroscopy

The following selected compounds [Cu_2_(EtNH_2_)_2_(µ-O_2_CCF_3_)_4_] (**1**), [Cu_2_(EtNH_2_)_2_(µ-O_2_CC_2_F_5_)_4_] (**2**), [Cu_2_(EtNH_2_)_2_(µ-O_2_CC_4_F_9_)_4_] (**4**), [Cu_2_(^i^PrNH_2_)_2_(µ-O_2_CCF_3_)_4_] (**7**), and [Cu_2_(^i^PrNH_2_)_2_(µ-O_2_CC_2_F_5_)_4_] (**8**) were studied by temperature variable infrared spectroscopy (VT IR) over the temperature range 303–753 K.

**Figure 4:**
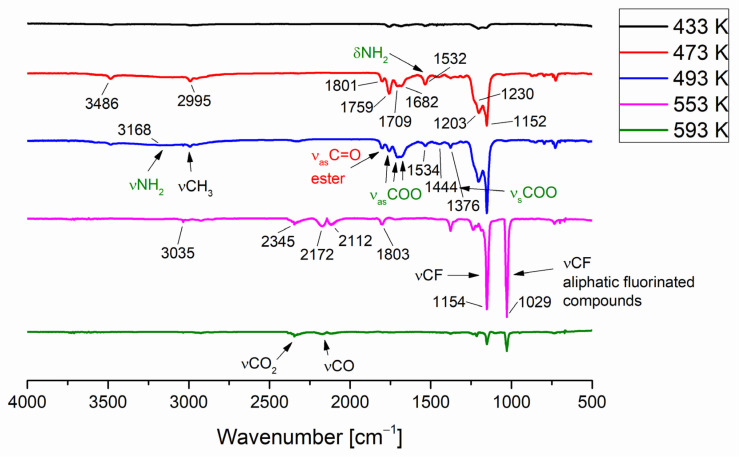
Temperature variable infrared spectra VT IR for the vapour formed during the [Cu_2_(EtNH_2_)_2_(µ-O_2_CCF_3_)_4_] complex (**1**) heating.

**Figure 5:**
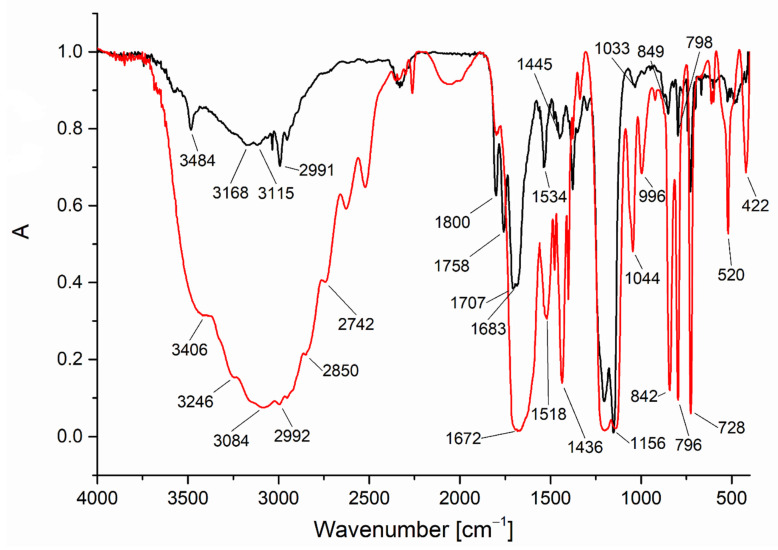
The spectra comparison for the [Cu_2_(EtNH_2_)_2_(µ-O_2_CCF_3_)_4_] complex (**1**) in the solid-state (red line) and the gas phase at 493 K (black line).

**Figure 6:**
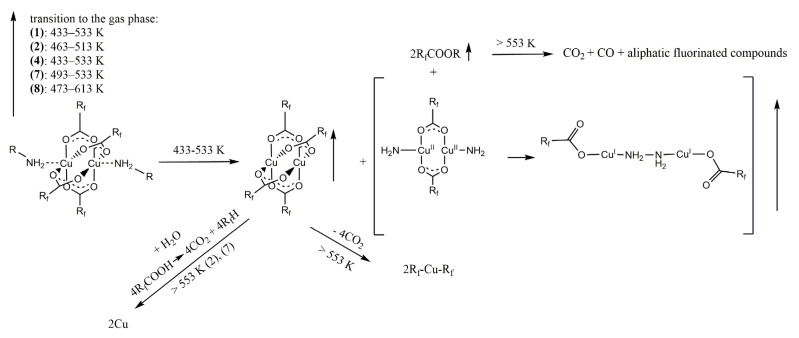
The thermal decomposition scheme for [Cu_2_(RNH_2_)_2_(μ-O_2_CR_f_)_4_] compounds (**1, 2, 4, 7,** and **8**) based on VT IR results.

In the case of the [Cu_2_(EtNH_2_)_2_(µ-O_2_CCF_3_)_4_] (**1**) four bands (1801 cm^−1^, 1759 cm^−1^, 1709 cm^−1^, and 1682 cm^−1^) within the range characteristic of C=O stretching vibrations were observed in the temperature between 433 and 533 K. The first is typical of gas-phase ethyl trifluoroacetate, which seems to be the decomposition product [37] The carboxylic acid formation was also considered, but in the spectrum of CF_3_COOH acid in the gas phase, the band νC=O band appears at 1826 cm^−1^ and 1788 cm^−1^ for the monomeric and the dimeric form, respectively [38,39,40]. Due to the existence of a band coordination shift relative to ν_as_COO for the free carboxylic acid, other bands in this area can be attributed to asymmetric vibrations of the coordinated COO group (Figure 4). Therefore, the lowest band (1682 cm^−1^) can be assigned to the ν_as_COO vibrations in the copper carboxylate [Cu_2_(µ-O_2_CCF_3_)_4_], as evidenced by the gas-phase spectrum measured for copper(II) pentafluoropropionate (Figure S29). In the solid-phase spectra, the v_as_COO band are shifted towards higher values for the amine-containing compounds when compared to the copper carboxylate (1647 cm^−1^ → 1672 cm^−1^; Figure S30 and Figure 5). Taking this fact into account, the band at 1709 cm^−1^ can be assigned to the vibration of ν_as_COO in the molecule of the complex (**1**) in the gas phase, which means shifting up 37 cm^−1^ with regard to the compound (**1**) in the solid phase (Figure 5). The coordinated ethylamine vibrations bands were also identified at 2995 cm^−1^ (νCH_3_), 3168 cm^−1^ (νNH_2_), and 1532 cm^−1^ (δNH_2_), which additionally confirmed the evaporation of the complex during heating. Interestingly, no bands characteristic of free ethylamine in the gas phase were observed in the spectra (3345 cm^−1^,1620 cm^−1^ [41]). The occurrence of the v_as_COO at 1759 cm^−1^ (shift towards higher values) indicates a change in the coordination mode from bidentate to unidentate [42] Combining this fact with the presence of the vNH_2_ at 3486 cm^−1^, the formation of the transitional copper(II) carboxylate complex with coordinated amido group (NH_2_^−^) in the gas phase, in which the coordination center is reduced to Cu(I) and a hydrazine-bridged complex is generated, has been proposed (Figure 6). A similar chemical reaction was observed for the nickel complexes. [43] The strongest registered signals came from the νCF stretching vibrations over the 1152–1203 cm^−1^ range. 

In the next step, at the temperature of 553 K, the band at 3035 cm^−1^ from the νCH_3_ stretching vibrations and at 1154 cm^−1^ from the νCF stretching vibrations were registered. Ethyl trifluoroacetate was still observed in the gas phase. Moreover, the identified bands at 2345 cm^−1^ and 2172 cm^−1^ can be assigned to the CO_2_ and CO molecules, respectively. A band characteristic of aliphatic fluorinated compounds (1029 cm^−1^) was also detected. The above data analysis leads to a conclusion that the [Cu_2_(EtNH_2_)_2_(µ-O_2_CCF_3_)_4_] complex (**1**) exists from 473 K to 533 K in the gas phase. Over this temperature, the compound (**1**) disappeared, and the decomposition products mixture was formed.

Similar products in the gas phase were observed in the VT IR spectra for compounds (**2**), (**4**), (**7**), and (**8**). Bands characteristic of the studied complexes were noted for each of them, and they are also shifted towards higher values in relation to the signals in the solid phase (Figures S31–S36). In conclusion, the compounds with ethylamine evaporate in the temperature range 433–533 K but those with isopropylamine in the range 473–613 K (Table 5).

The presence of carboxylic acid was identified for compounds (**2**) and (**7**) in the gas phase. In the case of the complex (**2**), relatively intense signals characteristic of water contamination in the gas phase were also observed in the spectra. Its presence may explain the formation of acid during decomposition. For the compound (**7**), the bands for water are not visible. The low intensity of the ν_as_COO band for the acid testifies to its little concentration in the gas phase. Therefore, the water content in the gas phase may be so low that it is invisible in the spectra but sufficient to form an acid. In addition, for the complex (**2**), the formation of CO_2_ was detected at the temperature of 413 K before the appearance of metal carriers in the gas phase. In the case of the compound (**4**), the bands characteristic of copper carboxylates with a coordinated amide (NH_2_^−^) group were not registered. The possible mechanisms of the decomposition of the tested compounds are shown in Figure 6.

The intensity of bands registered above 3000 cm^−1^ in the VT IR spectra of the compounds (**1**), (**2**), (**4**), (**7**), and (**8**) was lower than that in the solid phase, which confirms that in the gas phase, the number of hydrogen bonds decreased, as expected. Comparing all the [Cu_2_(RNH_2_)_2_(μ-O_2_CR_f_)_4_] complexes (R = Et, ^i^Pr, ^t^Bu), the earlier described compound [Cu_2_(^t^BuNH_2_)_2_(µ-O_2_CC_2_F_5_)_4_] with *tert*-butylamine [9,10] has the lowest evaporation temperature (413 K). On the other hand, the gas-phase complex occurs in the widest temperature range, in the case of [Cu_2_(^i^PrNH_2_)_2_(µ–O_2_CC_2_F_5_)_4_] (**8**). 

### 3.5. CVD Experiments

The above-mentioned studies in the gas phase indicated that from among the obtained complexes, [Cu_2_(EtNH_2_)_2_(µ-O_2_CCF_3_)_4_] (**1**), [Cu_2_(EtNH_2_)_2_(µ-O_2_CC_2_F_5_)_4_] (**2**), [Cu_2_(^i^PrNH_2_)_2_(µ-O_2_CCF_3_)_4_] (**7**), [Cu_2_(^i^PrNH_2_)_2_(µ-O_2_CC_2_F_5_)_4_] (**8**), and [Cu_2_(^i^PrNH_2_)_2_(µ-O_2_CC_3_F_7_)_4_] (**9**) revealed the best properties for the purposes of Chemical Vapor Deposition precursors formation. These are derivatives of the carboxylate ligands with shorter carbon chains (number of carbon atoms in the chain n = 1–3). For all the selected compounds ((**1**), (**2**), (**7**), (**8**), and (**9**)), deposits were obtained by the CVD method. The vaporisation temperatures T_V_ were 453 K (**7**) and 473 K (**1, 2, 8,** and **9**), whereas the deposition temperatures T_D_ values were from 573 K to 713 K (Table 6).

**Figure 7:**
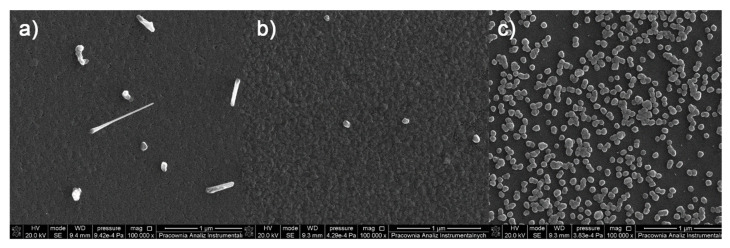
SEM images of deposits for the precursor [Cu_2_(EtNH_2_)_2_(µ-O_2_CCF_3_)_4_] (**1**), Mag = 100,000× ((**a**–**c**)—orderly with the increasing transport way), T_V_ = 473 K, T_D_ = 573 K, Si(111), Ar.

The surface morphology of the formed deposits from the complex [Cu_2_(EtNH_2_)_2_(µ-O_2_CCF_3_)_4_] (**1)** is differentiated due to the size, shape, and density of the objects (Figure 7) and depends on the length of the transport pathway in the CVD reactor. On the first covered silicone substrate (Figure 7a), sparsely distributed nanowires with the length of 300 nm to 800 nm in length and around 50 nm to 90 nm in diameter were observed. Instead, the surface of the sample (Figure 7b) is covered by a heterogeneous and rough deposit layer on which a few nanoparticles of 80 nm in diameter were visible. The surface of the next deposit (Figure 7c) is most different from the other two because it is covered by grains with of 40 nm to 70 nm in diameter that overlap in some areas.

**Figure 8:**
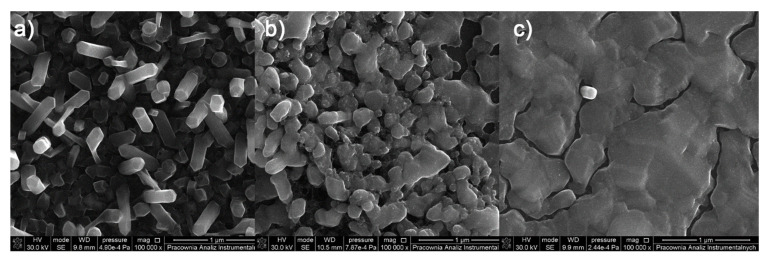
SEM pictures of deposits for the precursor [Cu_2_(EtNH_2_)_2_(µ-O_2_CC_2_F_5_)_4_] (**2**), Mag = 150,000×, ((**a**–**c**)—orderly with the increasing transport way), T_V_ = 473 K, T_D_ = 713 K, Si(111), Ar.

SEM images (Figure 8) for the compound [Cu_2_(EtNH_2_)_2_(µ-O_2_CC_2_F_5_)_4_] (**2**) show that the surface morphology of the obtained materials is diversified. The dependence of the deposit morphology on the length of the transport pathway of the precursor is evident. The coverage silicone substrate (a) (Figure 8a) morphology is extremely interesting. It consists of densely packed vertical copper nanorods with a diameter of 60 to 150 nm in diameter and approximately 500 nm in length. The material (b) (Figure 8b) is composed of densely packed grains with a size of 60–150 nm in size, which begin to interconnect with each other. The deposit (Figure 8c) shows that the grains virtually completely coalesce with the formation of a continuous layer. Considering data for the morphology of the obtained materials, it was found that the grains merged more and more intensively with the extension of the transport way. 

**Figure 9:**
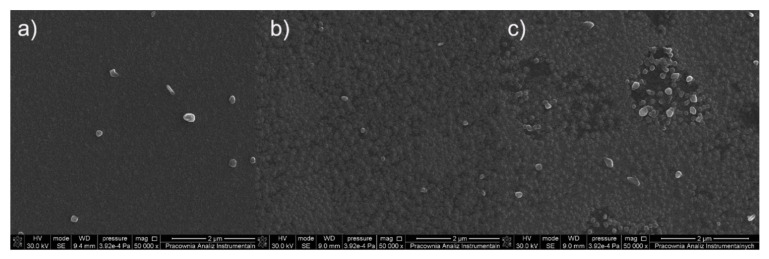
SEM pictures of deposits for the precursor [Cu_2_(^i^PrNH_2_)_2_(µ-O_2_CCF_3_)_4_] (**7**), Mag = 50,000×, ((**a**–**c**)-orderly with the increasing transport way), T_V_ = 453 K, T_D_ = 573 K, Si(111), Ar.

In order to check the effect of an amine on the type of deposits formed in the CVD process, complexes containing ^i^PrNH_2_ in the axial position were also used. The surface morphology of the obtained materials (a, b, c) (Figure 9) for the complex [Cu_2_(^i^PrNH_2_)_2_(µ-O_2_CCF_3_)_4_] (**7**) is heterogeneous. The Si(111) substrate (a) is covered by a rugged film on which oblong elements are visible. The deposit (b) consists of clusters of elements forming a rough surface. A similar situation is observed for the cover (c) but, this surface is even less homogeneous, and the areas with holes are visible. 

**Figure 10:**
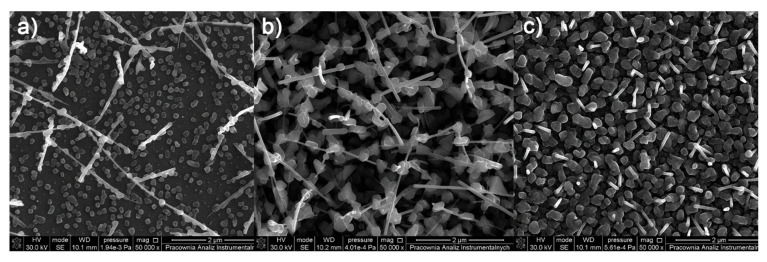
SEM pictures of deposits for the precursor [Cu_2_(^i^PrNH_2_)_2_(µ-O_2_CC_2_F_5_)_4_] (**8**), Mag = 50,000×, ((**a**–**c**)—orderly with the increasing transport pathway), T_V_ = 473 K, T_D_ = 713 K, Si(111), Ar.

As for the complex (**2**), in the case of the deposits obtained for the complex [Cu_2_(^i^PrNH_2_)_2_(µ-O_2_CC_2_F_5_)_4_] (**8**) (Figure 10), the influence of the precursor transport way on the surface morphology and the formation of nanowires was observed. However, when the compound (**8**) was used, the obtained nanorods were overgrown with droplet-shaped grains with a size of 80–200 nm in size (Figure 10a,b). The size of the nanorods (a) was 50–150 nm in diameter and about 900 nm in length, but the structures (b) were 20–50 nm in diameter and 500–900 nm in length. In the formed deposit (c), the packed grains with in size of 140–300 nm and nanorods in diameter of 30–70 nm and about 300–600 nm in length were grown. 

**Figure 11:**
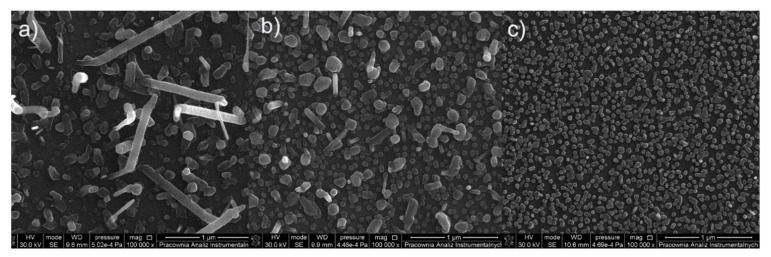
SEM pictures of deposits for the precursor [Cu_2_(^i^PrNH_2_)_2_(µ-O_2_CC_3_F_7_)_4_] (**9**), Mag = 100,000×, ((**a**–**c**)—orderly with the increasing transport way), T_V_ = 473 K, T_D_ = 673 K, Si(111), Ar.

Morphology of the compound [Cu_2_(^i^PrNH_2_)_2_(µ-O_2_CC_3_F_7_)_4_] (**9**) deposits (Figure 11) is also heterogeneous. On the surface (a), rods of about 1 µm and single grains are visible. The substrate (b) is covered by grains differing in shape (100–180 nm) and round, smaller elements (approx. 50nm in diameter) between which single rods are visible. In comparison, surface (c) is uniformly coated by small and round grains (30–80 nm).

**Figure 12:**
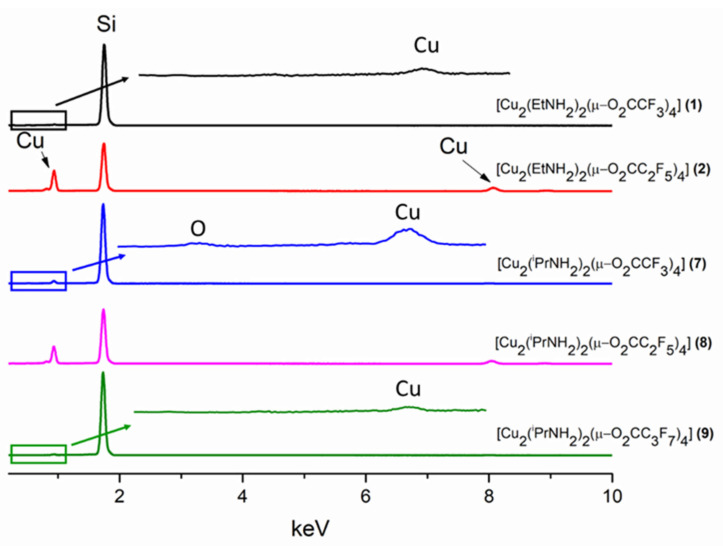
Composition analysis of the obtained materials using precursors: [Cu_2_(EtNH_2_)_2_(µ-O_2_CCF_3_)_4_] (**1**) (black line)_,_ [Cu_2_(EtNH_2_)_2_(µ-O_2_CC_2_F_5_)_4_] (**2**) (red line)_,_ [Cu_2_(^i^PrNH_2_)_2_(µ-O_2_CCF_3_)_4_] (**7**) (blue line)_,_ [Cu_2_(^i^PrNH_2_)_2_(µ-O_2_CC_2_F_5_)_4_] (**8**) (pink line)_,_ [Cu_2_(^i^PrNH_2_)_2_(µ-O_2_CC_3_F_7_)_4_] (**9**) (green line).

The EDX spectra (Figure 12) confirm the presence of copper in the obtained deposits. For the majority of materials, signals from oxygen, nitrogen, carbon, and fluorine, of which the precursor was composed of which, were not observed in the spectra. A slight peak corresponding to oxygen was recorded for the cover obtained from the complex (**7**). It may be due to the formation of small amounts of copper oxide. In all the cases, the evaporation temperatures were similar as opposed to the deposition temperatures. In the case of the precursors (**2**) and (**8**), the copper signal in the spectrum is the most intense because the resulting covers contain a densely packed material. This result may due to the better transport of the metal carriers in the gas phase and the higher decomposition temperature used.

### 3.6. Spin- and Dip-Coating Deposition

Since the first preliminary attempts to deposit nanomaterials using the ethylamine derivative in the CVD method failed, dip- and spin-coating methods were used to prepare thin layers of the gel-like copper complexes on a silicon substrate. The thus fabricated materials were then heated to decompose the compounds to produce thin copper oxide or copper layers.

#### 3.6.1. Spin-Coated Materials

**Figure 13:**
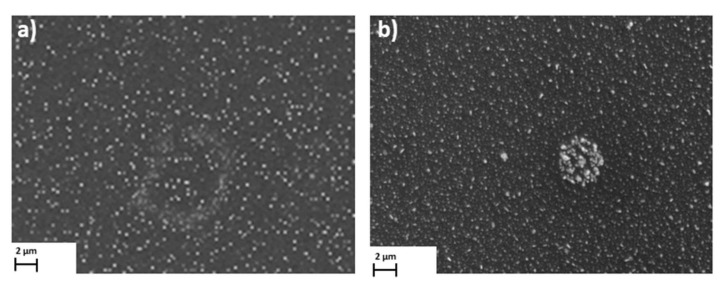
SEM images of [Cu_2_(EtNH_2_)_2_(µ-O_2_CC_3_F_7_)_4_] (**3**)/Si (**a**) before, (**b**) after heating Mag = 10,000× (1100 rpm, 30 s).

**Figure 14:**
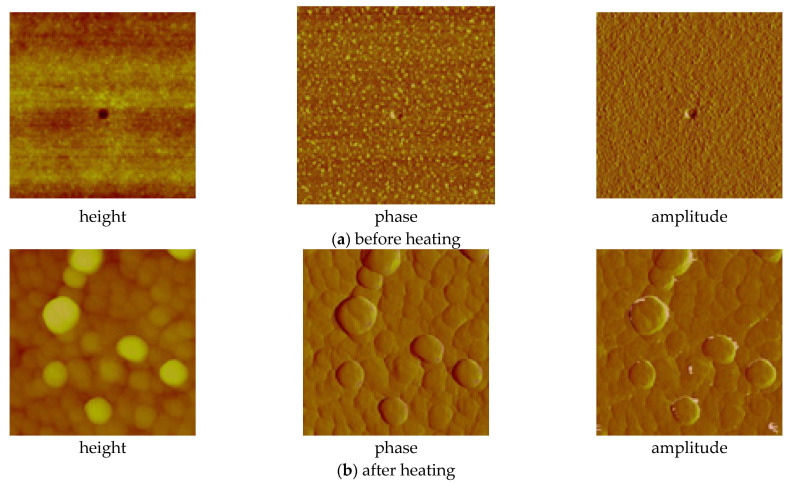
AFM images of [Cu_2_(EtNH_2_)_2_(µ-O_2_CC_3_F_7_)_4_] (**3**)/Si (**a**) before, (**b**) after heating (1100 rpm, 30 s).

In the case of the [Cu_2_(EtNH_2_)_2_(µ-O_2_CC_3_F_7_)_4_] complex (**3**) deposited on the Si substrate (at 1100 rpm, 30 s), the copper compound covered the surface evenly—in the dots shape (Figure 13). The phase AFM images show homogeneity of the covers (Figure 14a). One area of the complex layer was observed. The height (thickness) of the layers ranged from 2.5 to 25 nm.

After annealing, the dots become flatter and more extensive. The roughness parameters achieved R_a_ = 2.46 nm, R_q_ = 2.14 nm before heating, and R_a_ = 8.76 nm, R_q_ = 27.50 nm after heating, which points to the roughness increasing after the complex thermal decomposition Figure 14b. 

The application of the [Cu_2_(EtNH_2_)_2_(µ-O_2_CC_2_F_5_)_4_] complex (**2**) on Si(111) in the multistage spin coating process gave the formation of a new type of layers type, in which small islands of the compound occasionally appeared. The size of these islands did not exceed 1 µm. Depending on the spin coating conditions, the films with different arrangements were obtained. The complex formed grains of regular size and covered the surface evenly without empty spaces. The layer was smooth with R_a_ = 1.78 nm, R_q_ = 2.37 nm, and 15 nm thick. Similar to how it was observed for the [Cu_2_(EtNH_2_)_2_(µ-O_2_CC_3_F_7_)_4_] (**3**) materials, SEM analysis indicated the presence of the small crystallites in the complex surface. 

**Figure 15:**
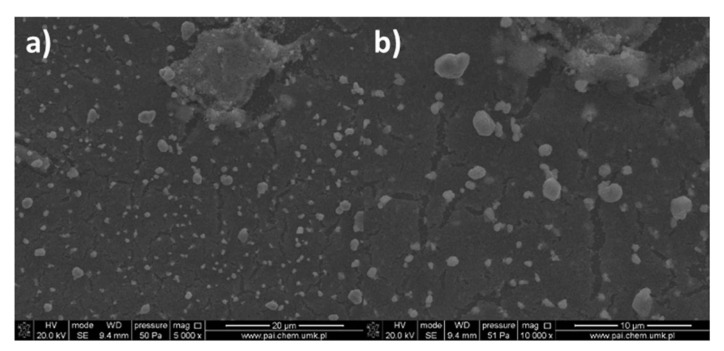
SEM images of [Cu_2_(EtNH_2_)_2_(µ-O_2_CC_2_F_5_)_4_] (**2**)/Si after heating, Cu 5.53 wt.%, O 10.48 wt.%, (800 rpm, 10 s, 600 rpm, 10 s ×2, drying 3000 rpm, 60 s) (**a**) Mag = 5000× (**b**) Mag = 10,000×.

**Figure 16:**
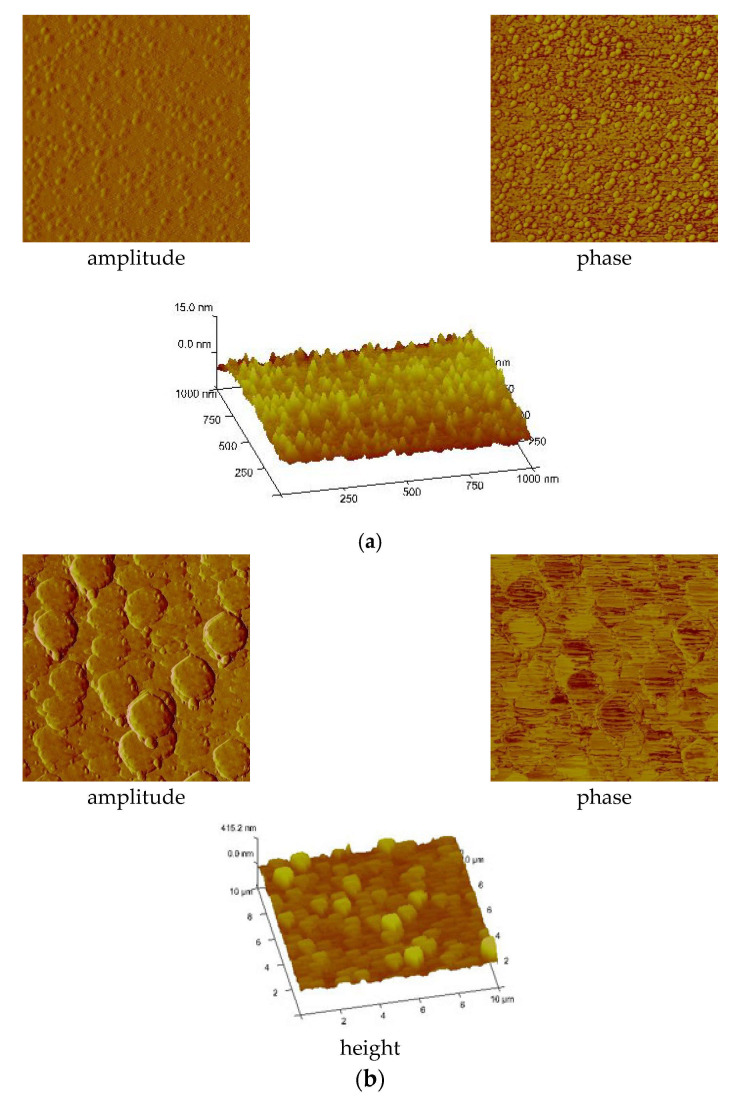
AFM images of [Cu_2_(EtNH_2_)_2_(µ-O_2_CC_2_F_5_)_4_] (**2**)/Si (**a**) before, (**b**) after heating (multistage process: 800 rpm, 10 s, 600 rpm, 10 s, 600 rpm, 10 s, drying 3000 rpm, 60 s).

After heating, the size of the grains increased, and sometimes the empty spaces appeared; the cracks were also noted resulting in inhomogeneous cover (Figure 15). This fact influenced the surface roughness, and the parameters R_a_ and R_q_ increased significantly, achieving 15.1 nm and 18.7 nm, respectively. Generally, the roughness of the obtained layers increased after annealing (Figure 16). Additionally, after heating the layers, the defects (spaces between compounds structures) increased twice from about 60 nm to 120 nm. As a consequence, the discontinuity of the films was observed. Different film thicknesses can be explained by the above-mentioned spin coating process as a result of which defects upon heating can be produced. Unequal evaporation of the in situ formed reaction products leads to partially cracked films of copper compounds films. A similar phenomenon was observed in the case of other copper and silver complexes [44].

EDX results for the [Cu_2_(EtNH_2_)_2_(µ-O_2_CC_3_F_7_)_4_] (**3**)/Si material formed at the spin speed 1000 rpm and time of coating 30 s were: Cu 1.69; N 9.80; F 10.44; O 5.0/Cu 2.10; O 6.11 wt.% (before/after heating). The same phenomenon was observed for [Cu_2_(EtNH_2_)_2_(µ-O_2_CC_2_F_5_)_4_]/Si (**2**) material (1100 rpm 30 s), and the following amount of copper: 0.78 wt.% before and 1.05 wt.% after heating were found. Moreover, the punctual copper quantity was much higher and achieved 20.4 wt.%. This situation results from the heating and an irregularly arrangement of a compound on the substrate surface (the presence of local islands of compounds). After heating, only Cu and O elements were detected as the result of the copper complex thermal decomposition. The lack of fluorine and nitrogen signals indicated the most probable formation of CuO as the final product. According to our expectations, the relative concentration of copper in the layers increased after heating. The above discussion suggests that the quality of the layer (uniformity, roughness) can be optimised by the spin speed and deposition time variation.

#### 3.6.2. Dip-Coated Materials

Dip coating was the second method used to obtain thin layers of both complexes (**2**) and (**3**). Considering the essential factors affecting the formation and quality of the fabricated covers, the effect of the following parameters was taken into account: the number of coating counts (varied from 5 to 30), immersion rate (from 20 to 80 mm/min), and the immersion time (over 5−120 s). Selected from among twenty-one, the four different sets of process parameters, for which the deposition effects were most promising, were chosen (Table 7). The influence of coating counts on the properties of the [Cu_2_(EtNH_2_)_2_(µ-O_2_CC_2_F_5_)_4_] (**2**) layers were studied as well. 

Again, the complex layers were heated to obtain copper oxide or thin copper materials.

**Figure 17:**
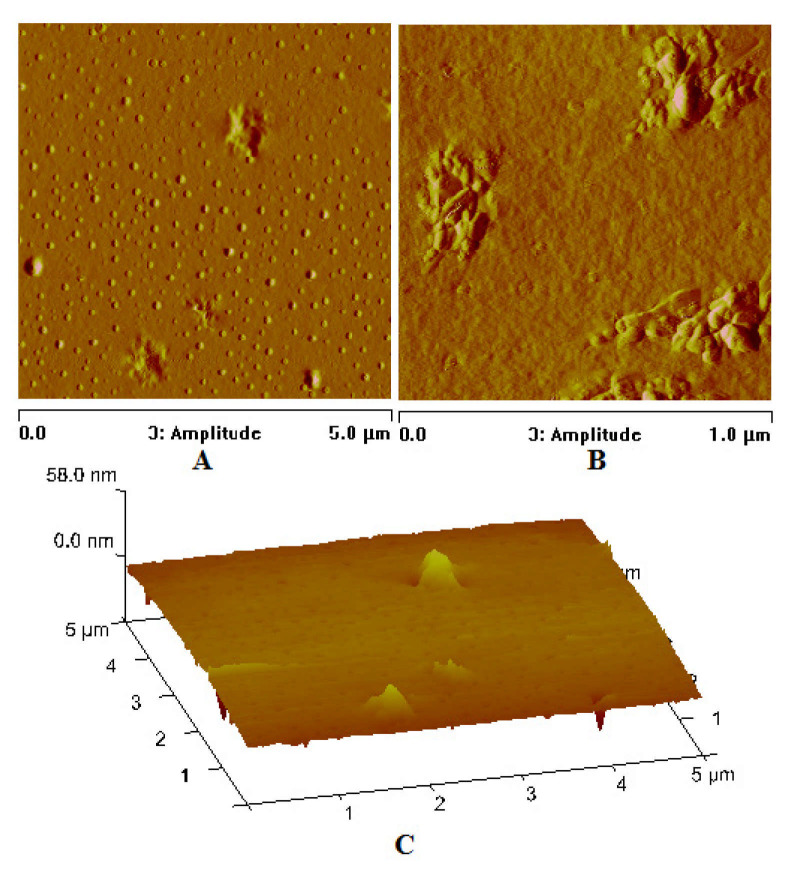
AFM images of [Cu_2_(EtNH_2_)_2_(µ-O_2_CC_3_F_7_)_4_] (**3**)/Si immersion time 30 s, amplitude Mag = 5 µm (**A**); Mag = 1 µm (**B**), height (**C**) before heating, dip coating, R_a_ = 2.90 nm, R_q_ = 3.98 nm.

**Figure 18:**
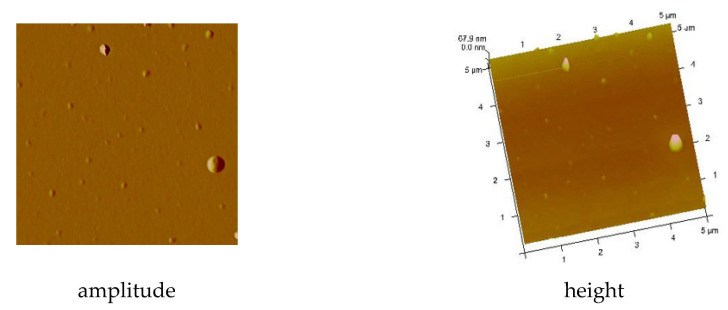
AFM images of [Cu_2_(EtNH_2_)_2_(µ-O_2_CC_2_F_5_)_4_] (**2**)/Si, Mag = 5 µm, dip coating before heating.

AFM results showed evenly distributed copper complexes on the silicon surface and layers without discontinuities. The phase AFM images exhibited only one area of the complex layers (Figure 17 and Figure 18). 

The covers of [Cu_2_(EtNH_2_)_2_(µ-O_2_CC_3_F_7_)_4_] (**3**) obtained at the immersion time 30 s exhibited tight surface; in some places, depressions and ridges appeared (R_a_ = 2.90 nm, R_q_ = 3.98 nm) (Figures S37, S38 and Figure 17). These layers are slightly rougher than the deposits obtained in the spin coating process (R_a_ = 2.46 nm, R_q_ = 2.14 nm). Reducing the immersion time to 20 s led to a more covered surface—the weight percent of silicon equals 10, which suggests a relatively good deposition of the copper complex on the surface. The best dip-coating parameters were immersion rate 10, immersion speed 80 mm/min, immersion time 20 s (Figures S15 and S39). The EDX analysis confirmed the presence of the complex by the percentage of copper 9.93 wt.%, oxygen 3.25 wt.%, fluoride 55.34 wt.%, carbon 6.29 wt.%, and nitrogen 4.22 wt.%. 

The [Cu_2_(EtNH_2_)_2_(µ-O_2_CC_2_F_5_)_4_] (**2**) complex forming materials obtained by deposition on Si(111) exhibited a thin, regular structure at the following roughness parameters as follows: R_a_ = 0.34–0.40 nm, R_q_ = 0.45–0.50 nm. In some cases, small, single crystallites appeared. These layers are smoother than those fabricated by the spin-coating method (R_a_ = 1.78 nm, R_q_ = 2.37 nm for SC). The phase AFM images show homogeneity of the covers (Figure 18). One area of the complex layer was observed. The height of the layer was equal to 68 nm. Additionally, the AFM analysis pointed out a difference in the materials depending on the coating counts (5 or 10). In both cases, the layers are smooth, but increasing the coating count leads to the layers in which the singular, randomly distributed crystallites appear.

**Figure 19:**
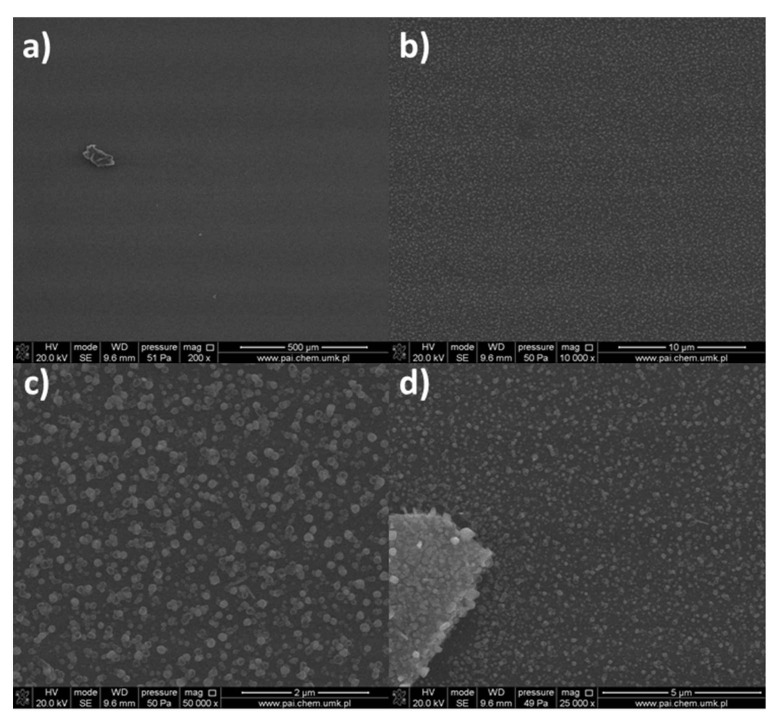
SEM images of [Cu_2_(EtNH_2_)_2_(µ-O_2_CC_3_F_7_)_4_]/Si (**3**), Mag = 200× (**a**), Mag = 10,000× (**b**), Mag = 50,000× (**c**), and Mag = 25,000× (**d**), immersion time 20 s, after heating.

**Figure 20:**
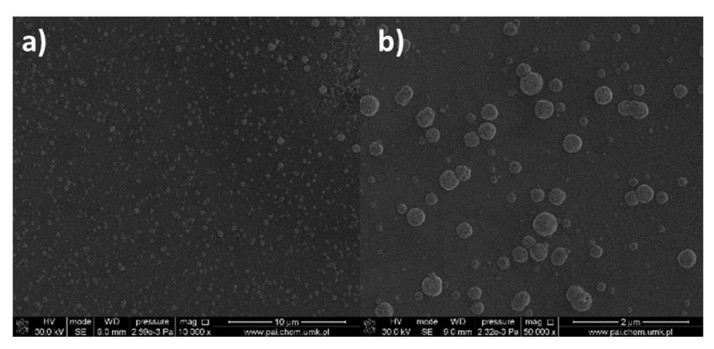
SEM images of [Cu_2_(EtNH_2_)_2_(µ-O_2_CC_3_F_7_)_4_] (**3**)/Si after heating, (**a**) Mag = 10,000×, (**b**) Mag = 50,000×.

**Figure 21:**
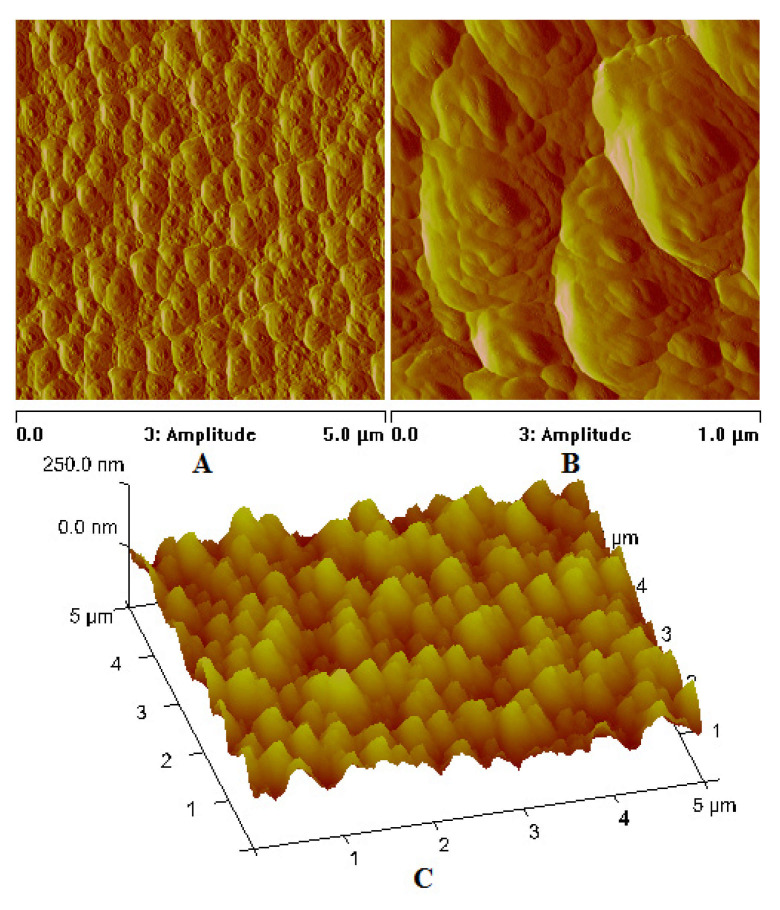
AFM images of [Cu_2_(EtNH_2_)_2_(µ-O_2_CC_3_F_7_)_4_] (**3**)/Si immersion time 20 s, Mag 5 µm (**A**); Mag 1 µm (**B**) height (**C**), after heating.

For all the dip-coated materials, the roughness of the layers increased after heating. The enhancement of the roughness is a consequence of the solvent lose and decomposition of the copper complex. Other authors observed the same effect in the case of silver and copper complexes [13,14,45]. After heating, the layers’ shape, structure, and composition were changed significantly. In the [Cu_2_(EtNH_2_)_2_(µ-O_2_CC_3_F_7_)_4_] (**3**)/Si materials, the grains of sizes from 5 to 500 nm, formed irregular aggregates. The regular grains were evenly dispersed on the silicon surface (Figure S40A and Figure 19, Figure 20, Figure 21).

Surprisingly, the relative copper content decreased several times in some cases (e.g., from 9.93 to 5.64 wt.%). The layers were smooth with R_a_ = 3.53 nm, R_q_ = 4.31 nm before heating and rough R_a_ = 33.4 nm, R_q_ = 42.3 nm after heating (Figure 21).

Similar results were observed in the case of nickel and cobalt films, which were achieved by a dip-coating process using acetolhydrazone (ALH) as a dissolving and reducing agent [46]. When the copper acetate and diethanoldiamine as solvent were used and the produced films were then heated, thin, homogenous, and smooth layers without cracks were obtained [25]. The films consisted of grains of sizes dependent on heating temperature. The roughness of these films was relatively low and ranged from 50 to 100 nm.

**Figure 22:**
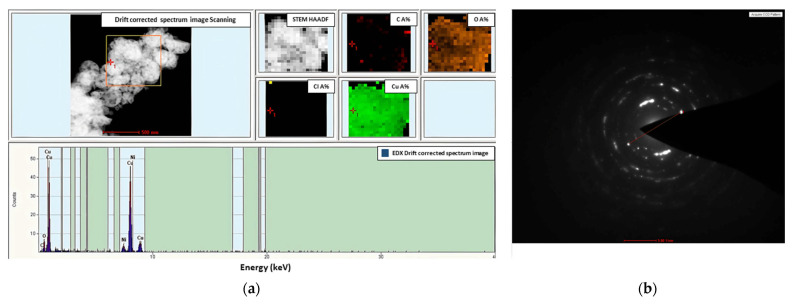
TEM data of the [Cu_2_(EtNH_2_)_2_(µ-O_2_CC_3_F_7_)_4_] (**3**)/Si after heating, (**a**) TEM image (top left), chemical mapping (top right) and EDX spectrum (bottom), (**b**) diffraction pattern d _h k l_ = 2/5 nm = 0.4 nm.

Additionally, for [Cu_2_(EtNH_2_)_2_(µ-O_2_CC_3_F_7_)_4_] (**3**)/Si covers after heating, TEM analysis was performed (Figure 22). TEM results confirmed the presence of copper (96.51 atomic %) and a small amount of oxygen in the layer, suggesting the metallic copper materials as the final product of thermal treatment. The diffraction analysis pointed to the d _h k l_ = 2/5 nm = 0.4 nm and confirmed the copper grains formation as the final heating product [47]. The materials were formed by spherical structures of around 50 nm in size and were also grouped, creating agglomerates.

**Figure 23:**
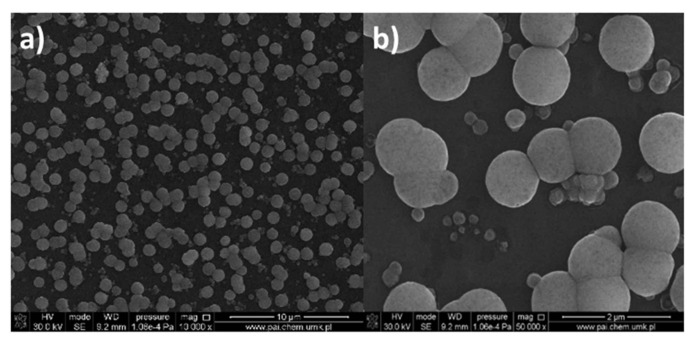
SEM images of [Cu_2_(EtNH_2_)_2_(µ-O_2_CC_2_F_5_)_4_] (**2**)/Si, after heating, (**a**) Mag = 10,000×, (**b**) Mag 50,000×.

Moreover, for [Cu_2_(EtNH_2_)_2_(µ-O_2_CC_2_F_5_)_4_] (**2**)/Si, the regular spherical shapes of 7–420 nm in dimension were observed (Figure S40B and Figure 23). These grains were regularly disposed on the silicon surface. These structures are unusual regarding materials obtained by the dip-coating method.

**Figure 24:**
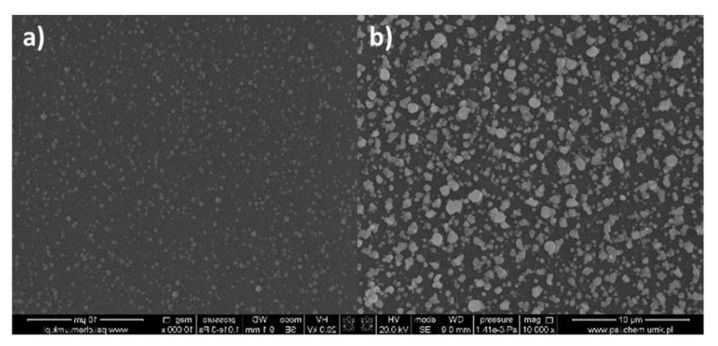
SEM image of [Cu_2_(EtNH_2_)_2_(µ-O_2_CC_3_F_7_)_4_] (**3**)/Si, immersion time 20 s, after heating, (**a**) the main, homogeneous cover, (**b**) the area at the bottom with the high deposit concentration

**Figure 25:**
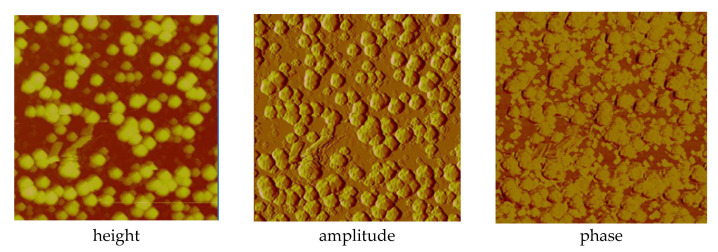
AFM images of [Cu_2_(EtNH_2_)_2_(µ-O_2_CC_3_F_7_)_4_] (**3**)/Si, immersion time 20 s. Mag 5 µm, after heating.

The EDX results indicated the presence of copper and oxygen suggesting copper oxide to be the thermal decomposition product. Carbon as an impurity was detected as well (a few weight percent). Grains were evenly spread on the silicon surface. Therefore, a tight, rough surface of materials without cracks was observed (Figure 24 and Figure 25). The surface roughness increased from several to tens of nanometres (R_a_ over the range 14.0–33.0 nm, R_q_ = 21.5–42.0 nm). The height of the layer is about 0.5 µm. The increase in the surface’s roughness was observed for materials resulting from applying both, the spin- and dip-coating methods.

The comparison of the layers obtained by two different wet methods, i.e., dip- and spin- coating indicates that the better results–thin, smooth materials without cracks with high copper content- were obtained by the dip-coating technique (immersion rate: 10 or 5, immersion speed 80 mm/min, immersion time 20 s). Moreover, the best conditions for the spin-coating process were 1000 rpm and of 30s coating time. Better results were obtained for the [Cu_2_(EtNH_2_)_2_(µ-O_2_CC_3_F_7_)_4_] (**3**) complex.

After heating, all the surfaces prepared by the spin-coating method change colour and roughness (from several to tens of nanometres). Additionally, the presence of cracks was noted. However, the films obtained by the dip-coating technique keep the tight structure, changing their colour and roughness (from several to tens of nanometres). This latter method can be an alternative to the CVD technique.

## 4. Conclusions

Copper(II) carboxylate complexes group with ethylamine and isopropylamine of the general formula [Cu_2_(RNH_2_)_2_(µ-O_2_CR_f_)_4_] (**1**–**10**), where R = Et, ^i^Pr, R_f_ = C_n_F_2n+1,_ n = 1–6, were synthesized in the reaction of copper(II) perfluorinated carboxylates with amines in situ generated from ethyl or isopropyl isocyanate. These compounds form blue gel-like solids. Based on the IR spectra analysis, the carboxylates bridging coordination mode and the axially N-bonded amine molecules were proposed, which results in the CuNO_4_ coordination sphere and the dimeric structure of the compounds in the solid state. The electron impact mass spectra analysis exhibited the occurrence of metallated species in the gas phase. The registered pseudomolecular ions [Cu_2_(EtNH_2_)_2_(µ-O_2_CR_f_)_3_]^+^ and [Cu_2_(^i^PrNH_2_)_2_(µ-O_2_CR_f_)_3_]^+^ confirmed the above proposed dimeric structure for the studied complexes both in the solid-state and in the gas phase. The thermal analysis showed that copper transfer to the gas phase took place at atmospheric pressure for complexes [Cu_2_(EtNH_2_)_2_(µ-O_2_CC_3_F_7_)_4_] (**3**), [Cu_2_(EtNH_2_)_2_(µ-O_2_CC_4_F_9_)_4_] (**4**), [Cu_2_(^i^PrNH_2_)_2_(μ-O_2_CC_2_F_5_)_4_] (**8**), and [Cu_2_(^i^PrNH_2_)_2_(μ-O_2_CC_3_F_7_)_4_] (**9**). In VT IR spectra, vapours of the (**1**) and (**4**) complexes vapours were observed over the temperature range 433–533 K, but in the case of the (**2**) compound—from 463 to 513 K. For the compounds containing isopropyl group bands, vapours of the corresponding complexes were noticed over 493–533 K (**7**) and 473–613 K (**8**). Therefore, the complexes with ethylamine require a lower evaporation temperature than precursors with isopropylamine.

The CVD experiments confirmed that the [Cu_2_(RNH_2_)_2_(µ-O_2_CR_f_)_4_] compounds are the source of metal carriers in the gas phase. The best-quality deposits consisting of pure copper were obtained using precursors [Cu_2_(EtNH_2_)_2_(µ-O_2_CC_2_F_5_)_4_] (**2**) and [Cu_2_(^i^PrNH_2_)_2_(μ-O_2_CC_2_F_5_)_4_] (**8**), the evaporation temperature of 473 K, and the deposition temperature of 713 K. Nanorods, nanoparticles, and densely packed grains are produced from the synthesised precursors in the CVD process. The great advantage of the [Cu_2_(RNH_2_)_2_(µ-O_2_CR_f_)_4_] compounds is that a metallic film can be obtained from the air-stable copper(II) compound with no need to use any additional reducing agent such as H_2_.

The procedure of the [Cu_2_(EtNH_2_)_2_(µ-O_2_CR_f_)_4_] complex layer formation was not as complicated as that for copper acetate described in the literature. The use of dip- and spin-coating indicates that better results, i.e., the thin, smooth materials without cracks, with a high copper content, can be obtained by dip-coating technique. The best conditions for the spin coating process were 1000 rpm and 30s coating time. The dip-coating parameters (for the [Cu_2_(EtNH_2_)_2_(µ-O_2_CC_3_F_7_)_4_] complex (**3**)) were as follows: immersion rate 10, immersion speed 80 mm/min, and immersion time 20 s. Heating the materials resulting from the dip- and spin-coating processes lead to thin copper metallic or copper oxide materials. In the case of the dip-coating method, spherical structures on the substrate surface were formed.

The presented results of EI MS, TG-IR, and VT IR studies confirmed the applicability of the proposed methodology for the CVD precursors selection. The thermal analysis for the temperature determination for the spin- and dip-coating materials heating was also proven.

## Figures and Tables

**Table 1:** Selected IR absorption bands (cm^−1^) of studied compounds (**1**–**10**).

Compound	νNH_2_ br	ν_as_COO	νNH_2 scci_	ν_s_COO	∆νCOO
**(1)** **[Cu_2_(EtNH_2_)_2_(µ-O_2_CCF_3_)_4_]**	3080	1672	1518	1436	236
**(2)** **[Cu_2_(EtNH_2_)_2_(µ-O_2_CC_2_F_5_)_4_]**	3080	1675	1531	1413	262
**(3)** **[Cu_2_(EtNH_2_)_2_(µ-O_2_CC_3_F_7_)_4_]**	3060	1679	1528	1405	274
**(4)** **[Cu_2_(EtNH_2_)_2_(µ-O_2_CC_4_F_9_)_4_]**	3070	1681	1531	1403	278
**(5)** **[Cu_2_(EtNH_2_)_2_(µ-O_2_CC_5_F_11_)_4_]**	3090	1679	1545	1409	270
**(6)** **[Cu_2_(EtNH_2_)_2_(µ-O_2_CC_6_F_13_)_4_]**	3070	1673	1527	1404	269
**(7)** **[Cu_2_(^i^PrNH_2_)_2_(µ-O_2_CCF_3_)_4_]**	3080	1666	1520	1431	235
**(8)** **[Cu_2_(^i^PrNH_2_)_2_(µ-O_2_CC_2_F_5_)_4_]**	3060	1675	1523	1403	272
**(9)** **[Cu_2_(^i^PrNH_2_)_2_(µ-O_2_CC_3_F_7_)_4_]**	3090	1683	1522	1406	277
**(10)** **[Cu_2_(^i^PrNH_2_)_2_(µ-O_2_CC_4_F_9_)_4_]**	3100	1681	1522	1403	278
EtNH_2_	3360	-	1625	-	-
^i^PrNH_2_	3380	-	1618	-	-

Δν(CF_3_COONa) = 223 cm^−1^, Δν(C_2_F_5_COONa) = 268 cm^−1^, Δν(C_n_F_2n+1_COONa; n = 3–6) = 272 cm^−1^; br, broad (approximate values).

**Table 2:** Mass spectrometry EI MS data of [Cu_2_(EtNH_2_)_2_(µ-O_2_CCF_3_)_4_] (**1**).

Fragments	*m/z*	RI[%]		
		305 K	355 K	600 K
[C_2_H_2_N]^+^	40	7	6	-
[C_2_H_3_N]^+^	41	11	8	-
[C_2_H_4_N]^+^	42	15	13	-
[CO_2_]^+^	44	35	33	100
[C_2_H_7_N]^+.^/[COOH]^+.^	45	45	69	3
[CF_2_]^+^	50	20	20	11
[CF_3_]^+^	69	93	100	61
[CF_3_CO]^+^	97	9	9	6
[Cu]^+^	63	40	26	1
[Cu_2_]^+∙^	126	6	7	-
[Cu_2_F]^+^	145	25	24	2
[Cu(EtNH_2_)(O_2_CCF_3_)]^+^	221	11	3	-
[Cu_2_(O_2_CCF_3_)]^+^	239	100	98	6
[Cu_2_(EtNH_2_)(O_2_CCF_3_)]^+^	284	5	2	-
[Cu_2_(O_2_CCF_3_)_2_]^+∙^	352	66	61	4
[Cu_2_(O_2_CCF_3_)_3_]^+^	465	7	1	-
[Cu_2_(EtNH_2_)(O_2_CCF_3_)_3_]^+^	510	11	3	-
[Cu_2_(EtNH_2_)_2_(O_2_CCF_3_)_3_]^+^	555	3	-	-
[Cu_3_(O_2_CCF_3_)_5_]^+^	756	2	-	-

**Table 3:** Mass spectrometry EI MS data of [Cu_2_(^i^PrNH_2_)_2_(µ-O_2_CCF_3_)_4_] (**7**).

Fragments	*m/z*	RI[%]		
		368 K	389 K	548 K
[C_2_H_2_N]^+^	40	1	50	3
[C_2_H_3_N]^+^	41	-	1	8
[C_2_H_4_N]^+^	42	1	1	6
[C_3_H_7_]^+^	43	42	80	2
[CO_2_]^+^	44	100	6	4
[C_2_H_7_N]^+^/[COOH]^+∙^	45	40	37	40
[CF_2_]^+^	50	8	67	52
[C_3_H_7_N]^+^	57	18	19	-
[C_3_H_8_N]^+^	58	69	1	-
[C_3_H_9_N]^+∙^	59	19	41	2
[CF_3_]^+^	69	44	2	1
[CF_3_CO]^+^	97	4	55	32
[Cu]^+^	63	-	20	44
[Cu(^i^PrNH_2_)]^+^	122	2	40	-
[Cu_2_]^+∙^	126	-	8	11
[Cu_2_F]^+^	145	-	29	39
[Cu(^i^PrNH_2_)(O_2_CCF_3_)]^+^	235	1	28	2
[Cu_2_(O_2_CCF_3_)]^+^	239	-	-	99
[Cu(^i^PrNH_2_)_2_(O_2_CCF_3_)]^+^	294	1	22	-
[Cu_2_(^i^PrNH_2_)(O_2_CCF_3_)]^+^	298	1	14	-
[Cu_2_(O_2_CCF_3_)_2_]^+∙^	352	-	63	45
[Cu_2_(O_2_CCF_3_)_3_]^+ a)^	465	-	-	-
[Cu_2_(^i^PrNH_2_)(O_2_CCF_3_)_3_]^+^	524	-	12	-
[Cu_2_(^i^PrNH_2_)_2_(O_2_CCF_3_)_3_]^+^	583	5	77	-

^a)^ The **[Cu_2_(O_2_CCF_3_)_3_]^+^_2_** ion (465 *m/z*) was detected but not at the temperatures selected for Table 3.

**Table 4:** Thermal analysis data.

Complex	Temperature (K)			Residue (%)	
	T_i_	T_m_	T_f_	Found	Calc.
**[Cu_2_(EtNH_2_)_2_(μ-O_2_CCF_3_)_4_] (1)**	410	486	532	18.17	18.99 (Cu)
**[Cu_2_(EtNH_2_)_2_(μ-O_2_CC_3_F_7_)_4_] (3)**	395	466	505	6.03	11.88 (Cu)
**[Cu_2_(EtNH_2_)_2_(μ-O_2_CC_4_F_9_)_4_] (4)**	388	476	520	6.97	10.01 (Cu)
**[Cu_2_(^i^PrNH_2_)_2_(μ-O_2_CCF_3_)_4_] (7)**	312	474	528	22.9	22.8 (CuO)
**[Cu_2_(^i^PrNH_2_)_2_(μ-O_2_CC_2_F_5_)_4_] (8)**	323	449	478	4.72	17.7 (CuO)
**[Cu_2_(^i^PrNH_2_)_2_(μ-O_2_CC_3_F_7_)_4_] (9)**	308	464	548	7.2	14.5 (CuO)
**[Cu_2_(^i^PrNH_2_)_2_(μ-O_2_CC_4_F_9_)_4_] (10)**	355	463	510	14.4	12.2 (CuO)

T_i_, initial temperature; T_m_, maximum temperature; T_f_, final temperature.

**Table 5:** Temperature ranges of occurrence of decomposition products in the gas phase for compounds (**1**), (**2**), (**4**), (**7**), and (**8**).

The Product in the Gas Phase	Temperature Range [K]				
	(1)	(2)	(4)	(7)	(8)
[Cu_2_(RNH_2_)_2_(O_2_CR_f_)_4_]	433–533	463–513	433–533	493–533	473–613
[Cu_2_(O_2_CR_f_)_4_]	433–533	463–513	433–513	433–533	473–613
[Cu_2_(NH_2_)_2_(O_2_CR_f_)_2_]	433–533	≥463	473–533 *	433–533	≥473
R_f_COOR	433–573	≥463	≥433	≥453	533–613
R_f_COOH	–	473–513	–	≥513	–
CO_2_	≥553	≥413	≥533	≥493	≥473
CO	≥553	–	533–553	≥553	553–613
aliphatic fluorinated compounds	≥553	–	≥533	–	–

* Very low concentration.

**Table 6:** Summary CVD conditions for experiments.

	Vaporization Temperature T_V_ [K]	Deposition Temperature T_D_ [K]	Precursor Mass (m) [mg]	Carrier Gas	Substrate	Deposition Time (t) [min]
**[Cu_2_(EtNH_2_)_2_(µ-O_2_CCF_3_)_4_] (1)**	473	573	80	Ar	Si(111)	60
**[Cu_2_(EtNH_2_)_2_(µ-O_2_CC_2_F_5_)_4_] (2)**	473	713	100			
**[Cu_2_(^i^PrNH_2_)_2_(µ-O_2_CCF_3_)_4_] (7)**	453	573	80			
**[Cu_2_(^i^PrNH_2_)_2_(µ-O_2_CC_2_F_5_)_4_] (8)**	473	713	100			
**[Cu_2_(^i^PrNH_2_)_2_(µ-O_2_CC_3_F_7_)_4_] (9)**	473	673	100			

**Table 7:** The list of selected parameters of the dip-coating process for the best quality layers of [Cu_2_(EtNH_2_)_2_(µ-O_2_CC_3_F_7_)_4_] (**3**).

Set Number	Coating Counts	Immersion Rate [mm/min]	Immersion Time[s]
1	10	80	5
2	10	80	20
3	10	80	30
4	30	80	10

## Data Availability

The data presented in this study are available on request from the corresponding author.

## Supplementary Materials

The following are available online at www.mdpi.com/article/10.3390/ma14237451/s1, Table S1 Mass spectrometry EI MS data of [Cu_2_(EtNH_2_)_2_(µ-O_2_CC_2_F_5_)_4_] (2); Table S2 Mass spectrometry EI MS data of [Cu_2_(EtNH_2_)_2_(µ-O_2_CC_3_F_7_)_4_] (3); Table S3 Mass spectrometry EI MS data of [Cu_2_(EtNH_2_)_2_(µ-O_2_CC_4_F_9_)_4_] (4); Table S4 Mass spectrometry EI MS data of [Cu_2_(EtNH_2_)_2_(µ-O_2_CC_5_F_11_)_4_] (5); Table S5 Mass spectrometry EI MS data of [Cu_2_(EtNH_2_)_2_(µ-O_2_CC_6_F_13_)_4_] (6); Table S6 Mass spectrometry EI MS data of [Cu_2_(^i^PrNH_2_)_2_(µ-O_2_CC_2_F_5_)_4_] (8); Table S7 Mass spectrometry EI MS data of [Cu_2_(^i^PrNH_2_)_2_(µ-O_2_CC_3_F_7_)_4_] (9); Table S8 Mass spectrometry EI MS data of [Cu_2_(^i^PrNH_2_)_2_(µ-O_2_CC_4_F_9_)_4_] (10); Figure S1 Infrared spectrum (thin film on KBr plates) of [Cu_2_(EtNH_2_)_2_(µ-O_2_CCF_3_)_4_] (1); Figure S2 Infrared spectrum (thin film on KBr plates) of [Cu_2_(EtNH_2_)_2_(µ-O_2_CC_2_F_5_)_4_] (2); Figure S3 Infrared spectrum (thin film on KBr plates) of [Cu_2_(EtNH_2_)_2_(µ-O_2_CC_3_F_7_)_4_] (3); Figure S4 Infrared spectrum (thin film on KBr plates) of [Cu_2_(EtNH_2_)_2_(µ-O_2_CC_4_F_9_)_4_] (4); Figure S5 Infrared spectrum (thin film on KBr plates) of [Cu_2_(EtNH_2_)_2_(µ-O_2_CC_5_F_11_)_4_] (5); Figure S6 Infrared spectrum (thin film on KBr plates) of [Cu_2_(EtNH_2_)_2_(µ-O_2_CC_6_F_13_)_4_] (6); Figure S7 ATR-IR spectrum of [Cu_2_(^i^PrNH_2_)_2_(µ-O_2_CCF_3_)_4_] (7); Figure S8 ATR-IR spectrum of [Cu_2_(^i^PrNH_2_)_2_(µ-O_2_CC_2_F_5_)_4_] (8); Figure S9 ATR-IR spectra of [Cu_2_(^i^PrNH_2_)_2_(µ-O_2_CC_3_F_7_)_4_] (9); Figure S10 ATR-IR spectrum of [Cu_2_(^i^PrNH_2_)_2_(µ-O_2_CC_3_F_7_)_4_] (10); Figure S11 Mass spectrometry EI MS spectra of [Cu_2_(EtNH_2_)_2_(µ-O_2_CCF_3_)_4_] (1) at temperature: (a) 305 K, (b) 355 K, (c) 600 K and (d) simulation of isotopic pattern for a pseudomolecular ion [Cu_2_(EtNH_2_)_2_(O_2_CCF_3_)_3_]^+^; Figure S12 Mass spectrometry EI MS spectra of [Cu_2_(EtNH_2_)_2_(µ-O_2_CC_2_F_5_)_4_] (2) at temperature: (a) 310 K, (b) 331 K, (c) 357 K (d) 542 K and (e) simulation of isotopic pattern for a pseudomolecular ion [Cu_2_(EtNH_2_)_2_(O_2_CC_2_F_5_)_3_]^+^; Figure S13 Mass spectrometry EI MS spectra of [Cu_2_(EtNH_2_)_2_(µ-O_2_CC_3_F_7_)_4_] (3) at temperature: (a) 357 K, (b) 417 K, (c) 463 K (d) 542 K and (e) simulation of isotopic pattern for a pseudomolecular ion [Cu_2_(EtNH_2_)_2_(O_2_CC_3_F_7_)_3_]^+^; Figure S14 Mass spectrometry EI MS spectra of [Cu_2_(EtNH_2_)_2_(µ-O_2_CC_4_F_9_)_4_] (4) at temperature: (a) 305 K, (b) 345 K, (c) 424 K (d) 590 K and (e) simulation of isotopic pattern for a pseudomolecular ion [Cu_2_(EtNH_2_)_2_(O_2_CC_4_F_9_)_3_]^+^; Figure S15 Mass spectrometry EI MS spectra of [Cu_2_(EtNH_2_)_2_(µ-O_2_CC_5_F_11_)_4_] (5) at temperature: (a) 319 K, (b) 425 K, (c) 590 K and (d) simulation of isotopic pattern for a pseudomolecular ion [Cu_2_(EtNH_2_)_2_(O_2_CC_5_F_11_)_3_]^+^; Figure S16 Mass spectrometry EI MS spectra of [Cu_2_(EtNH_2_)_2_(µ-O_2_CC_6_F_13_)_4_] (6) at temperature: (a) 305 K, (b) 398 K, (c) 431 K (d) 616 K and (e) simulation of isotopic pattern for a pseudomolecular ion [Cu_2_(EtNH_2_)_2_(O_2_CC_6_F_13_)_3_]^+^; Figure S17 Mass spectrometry EI MS spectra of [Cu_2_(^i^PrNH_2_)_2_(µ-O_2_CCF_3_)_4_] (7) at temperature: (a) 368 K, (b) 389 K, (c) 548 and (d) simulation of isotopic pattern for a pseudomolecular ion [Cu_2_(^i^PrNH_2_)_2_(O_2_CCF_3_)_3_]^+^; Figure S18 Mass spectrometry EI MS spectra of [Cu_2_(^i^PrNH_2_)_2_(µ-O_2_CC_2_F_5_)_4_] (8) at temperature: (a) 357 K, (b) 404 K, (c) 458 K, (d) 505 K and (e) simulation of isotopic pattern for a pseudomolecular ion [Cu_2_(^i^PrNH_2_)_2_(O_2_CC_2_F_5_)_3_]^+^; Figure S19 Mass spectrometry EI MS spectra of [Cu_2_(^i^PrNH_2_)_2_(µ-O_2_CC_3_F_7_)_4_] (9) at temperature: (a) 340 K, (b) 372 K, (c) 499 K, (d) 544 K and (e) simulation of isotopic pattern for a pseudomolecular ion [Cu_2_(^i^PrNH_2_)_2_(O_2_CC_3_F_7_)_3_]^+^; Figure S20 Mass spectrometry EI MS spectra of [Cu_2_(^i^PrNH_2_)_2_(µ-O_2_CC_4_F_9_)_4_] (10) (a) 369 K, (b) 388 K, (c) 401 K, (d) 490 K and (e) simulation of isotopic pattern for a pseudomolecular ion [Cu_2_(^i^PrNH_2_)_2_(O_2_CC_4_F_9_)_3_]^+^; Figure S21 Thermal decomposition of [Cu_2_(EtNH_2_)_2_(µ-O_2_CCF_3_)_4_] (1) (TG, DTG, DTA curves); Figure S22 TGA/IR spectrum of [Cu_2_(EtNH_2_)_2_(µ-O_2_CCF_3_)_4_] (1) registered at temperature 423 K; Figure S23 Thermal decomposition of [Cu_2_(EtNH_2_)_2_(µ-O_2_CC_3_F_7_)_4_] (3) (TG, DTG, DTA curves); Figure S24 Thermal decomposition of [Cu_2_(EtNH_2_)_2_(µ-O_2_CC_4_F_9_)_4_] (4) (TG, DTG, DTA curves); Figure S25 Thermal decomposition of [Cu_2_(^i^PrNH_2_)_2_(µ-O_2_CCF_3_)_4_] (7); Figure S26 Thermal decomposition of [Cu_2_(^i^PrNH_2_)_2_(µ-O_2_CC_2_F_5_)_4_] (8); Figure S27 Thermal decomposition of [Cu_2_(^i^PrNH_2_)_2_(µ-O_2_CC_3_F_7_)_4_] (9); Figure S28 Thermal decomposition of [Cu_2_(^i^PrNH_2_)_2_(µ-O_2_CC_3_F_7_)_4_] (10); Figure S29 Temperature variable infrared spectra VT IR of vapors formed during the [Cu_2_(µ-O_2_CC_2_F_5_)_4_] heating; Figure S30 Infrared spectra for the [Cu_2_(µ-O_2_CCF_3_)_4_] in the solid state; Figure S31 Temperature variable infrared spectra VT IR of vapors formed during the [Cu_2_(EtNH_2_)_2_(µ-O_2_CC_2_F_5_)_4_] complex (2) heating; Figure S32 The spectra comparison for the [Cu_2_(EtNH_2_)_2_(µO_2_CC_2_F_5_)_4_] complex (2) in the solid state (orange line) and in the gas phase at 473 K (blue line); Figure S33 Temperature variable infrared spectra VT IR of vapors formed during the [Cu_2_(EtNH_2_)_2_(µ-O_2_CC_4_F_9_)_4_] complex (4) heating; Figure S34 The spectra comparison for the [Cu_2_(EtNH_2_)_2_(µO_2_CC_4_F_9_)_4_] complex (4) a) in the solid state (red line) and in the gas phase at 493 K (black line) b) at temperature 493 K and 533 K; Figure S35 Temperature variable infrared spectra VT IR for the vapor formed during the [Cu_2_(^i^PrNH_2_)_2_(µ-O_2_CCF_3_)_4_] complex (7) heating; Figure S36 Temperature variable infrared spectra VT IR of vapors formed during the [Cu_2_(^i^PrNH_2_)_2_(µ-O_2_CC_2_F_5_)_4_] complex (8) heating 433-733K and at temperature 493 K (red line); Figure S37 Optical microscope images of [Cu_2_(EtNH_2_)_2_(µ-O_2_CC_3_F_7_)_4_](3)/Si before heating, immersion time 30, before heating (A) the immersion line, (B) middle of the layer; Figure S38 SEM image of [Cu_2_(EtNH_2_)_2_(µ-O_2_CC_3_F_7_)_4_](**3**)/Si immersion time 30, Mag = 200x, before heating; Figure S39 Optical microscope images of [Cu_2_(EtNH_2_)_2_(µ-O_2_CC_3_F_7_)_4_](**3**)/Si, before heating, immersion time 20, coating count 10, Mag 5x, lower part of the substrate (A), middle of the layer (B), immersion line (C); Figure S40 Images from optical microscope after heating (A) [Cu_2_(EtNH_2_)_2_(µ-O_2_CC_3_F_7_)_4_](**3**)/Si immersion time 20; (B) [Cu_2_(EtNH_2_)_2_(µ-O_2_CC_2_F_5_)_4_](**2**)/Si immersion time 20.
