# Supplementary material for: Copper(II) Perfluorinated Carboxylate Complexes with Small Aliphatic Amines as Universal Precursors for Nanomaterial Fabrication
Source: Materials (Basel). 2021 Dec 4;14(23):7451. doi: 10.3390/ma14237451 (PMC8659220; doi:10.3390/ma14237451)
Supplement: Supplementary file 1 [file materials-14-07451-s001.zip › materials-1468918-supplementary.pdf]

materials

MDPI

Supplementary Materials

Copper(II) Perfluorinated Carboxylate Complexes with Small Aliphatic Amines as Universal Precursors for Nanomaterial Fabrication

Iwona B. Szymańska *, Katarzyna Madajska, Aleksandra Butrymowicz and Magdalena Barwiołek

Faculty of Chemistry, Nicolaus Copernicus University in Toruń, Gagarina 7, 87-100 Toruń, Poland; 502533@doktorant.umk.pl (K.M.); aleksandra.butrymowicz@doktorant.umk.pl (A.B.); mbarwiolek@umk.pl (M.B.)

* Correspondence: pola@umk.pl; Tel.: +48-566114317

Citation: Szymańska, I.B.; Madajska, K.; Butrymowicz, A.; Barwiołek, M. Copper(II) Perfluorinated Carboxylate Complexes with Small Aliphatic Amines as Universal Precursors for Nanomaterial Fabrication. *Materials* **2021**, *14*, 7451. https://doi.org/10.3390/ma14237451

Academic Editor: Piotr Smoleński

Received: 2 November 2021
Accepted: 30 November 2021
Published: 4 December 2021

Publisher's Note: MDPI stays neutral with regard to jurisdictional claims in published maps and institutional affiliations.

Copyright: © 2021 by the authors. Licensee MDPI, Basel, Switzerland. This article is an open access article distributed under the terms and conditions of the Creative Commons Attribution (CC BY) license (http://creativecommons.org/licenses/by/4.0/).

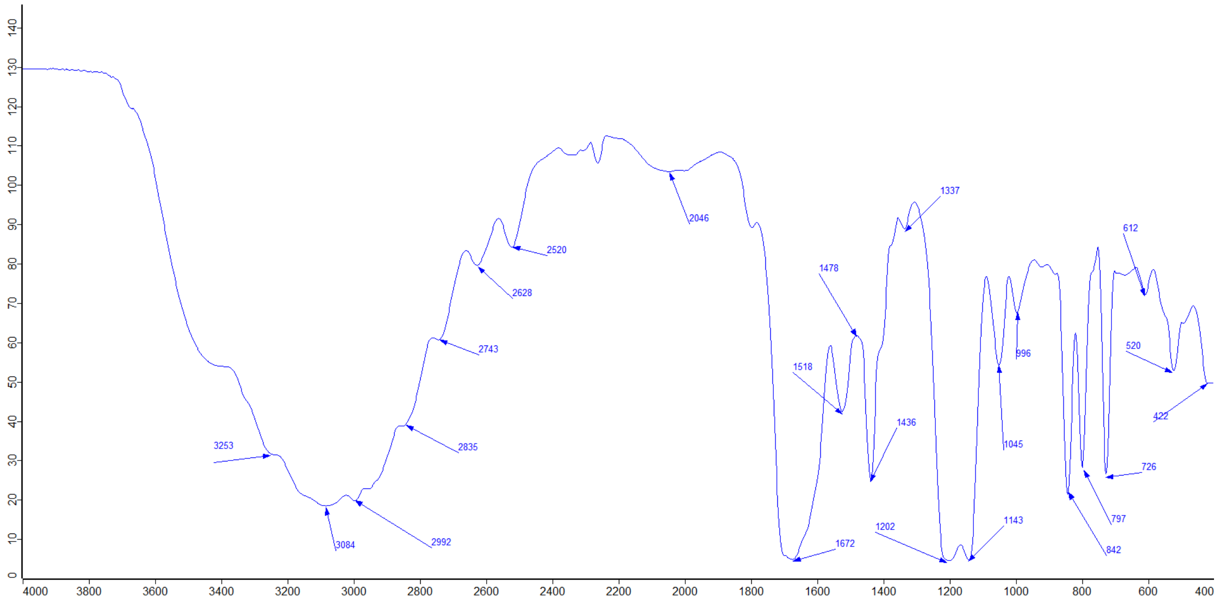

Figure S1. Infrared spectrum (thin film on KBr plates) of $[Cu_2(EtNH_2)_2(\mu\text{-}O_2CCF_3)_4]$ (1).

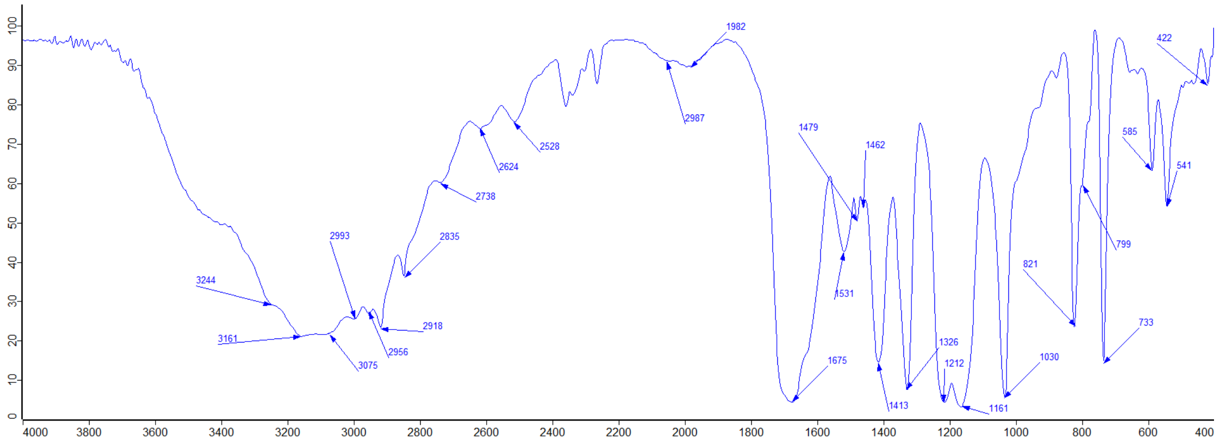

Figure S2. Infrared spectrum (thin film on KBr plates) of $[Cu_2(EtNH_2)_2(\mu\text{-}O_2CC_2F_5)_4]$ (2).

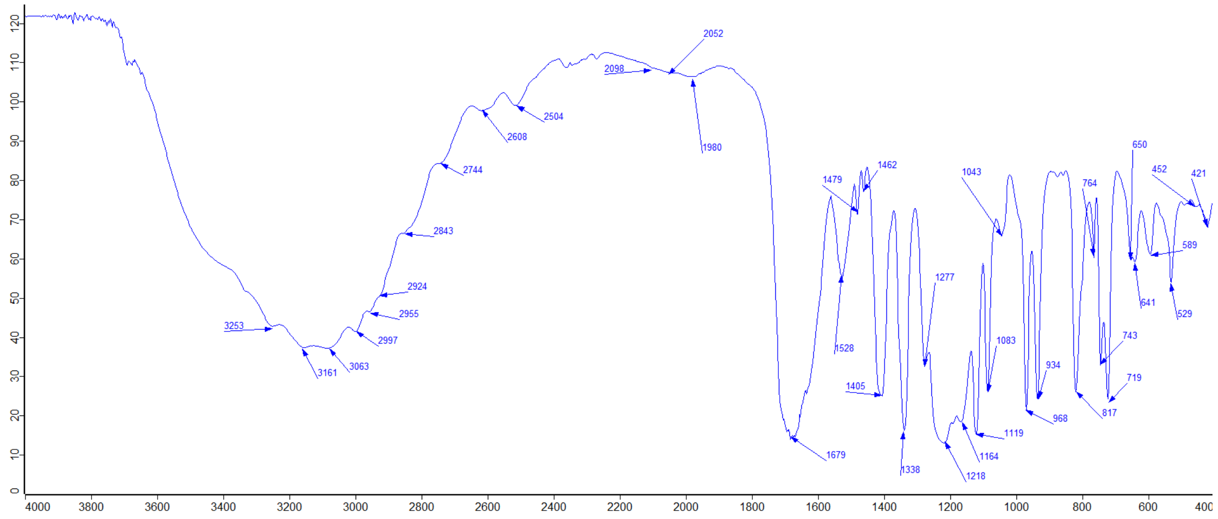

Figure S3. Infrared spectrum (thin film on KBr plates) of [$Cu_2(EtNH_2)_2(\mu\text{-}O_2CC_3F_7)_4$] (3).

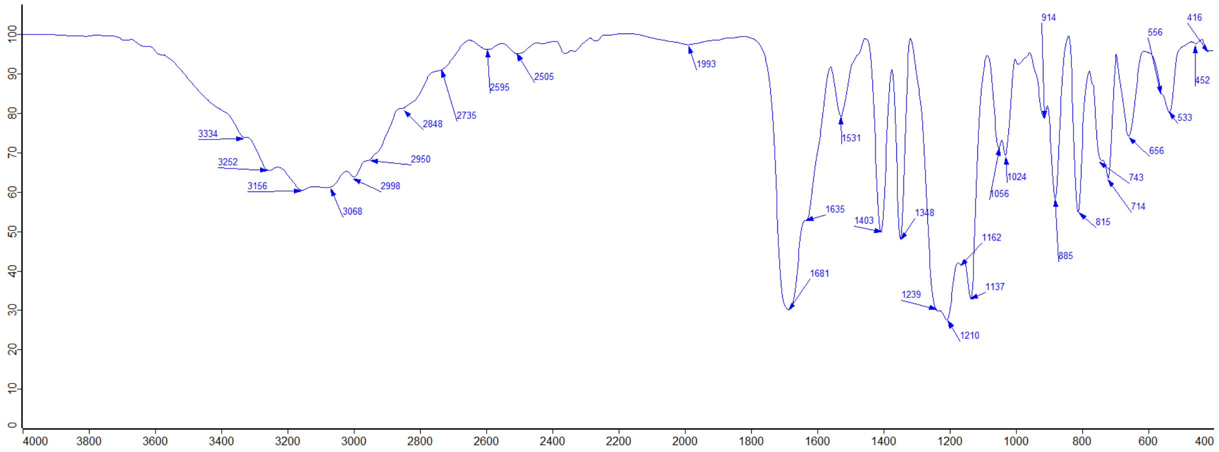

Figure S4. Infrared spectrum (thin film on KBr plates) of [$Cu_2(EtNH_2)_2(\mu\text{-}O_2CC_4F_9)_4$] (4).

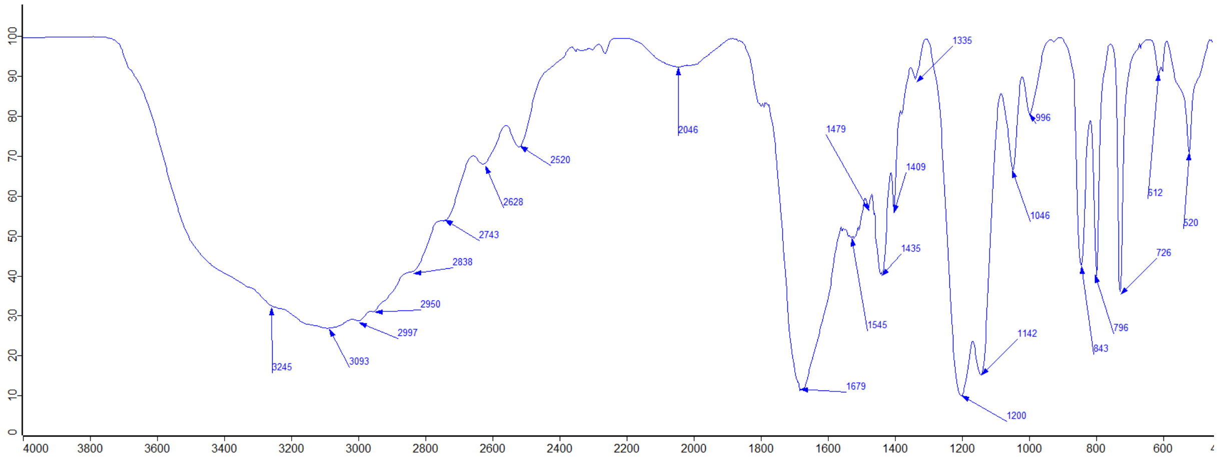

Figure S5. Infrared spectrum (thin film on KBr plates) of [$Cu_2(EtNH_2)_2(\mu\text{-}O_2CC_5F_{11})_4$] (5).

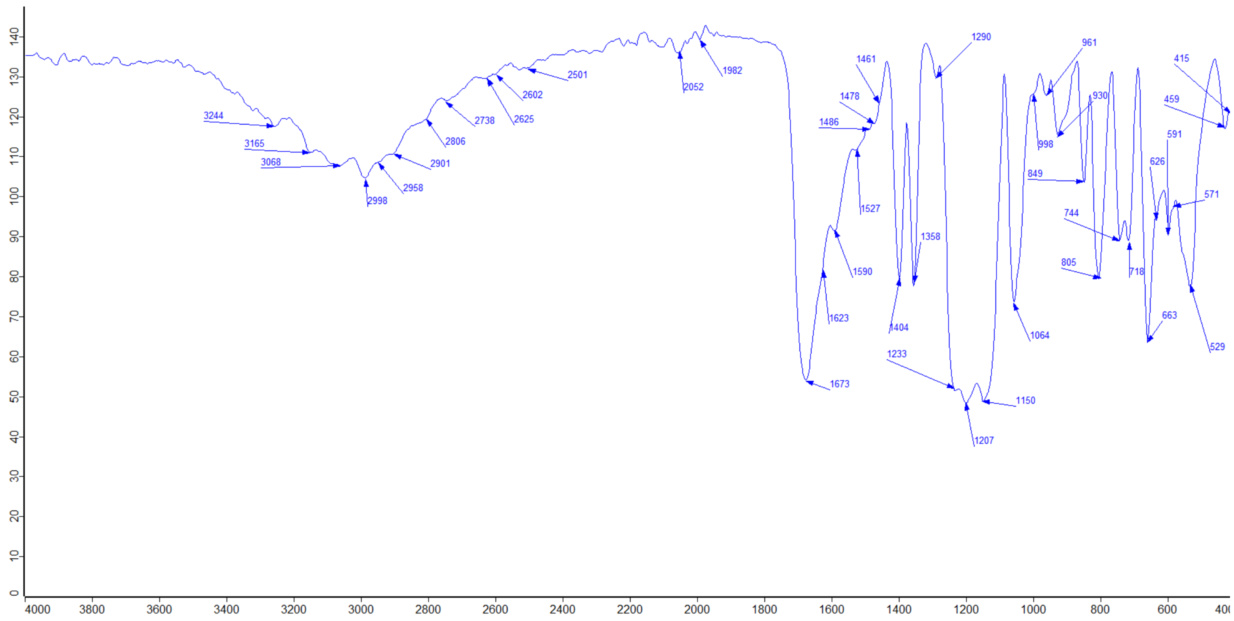

Figure S6. ATR-IR spectrum $[Cu_2(EtNH_2)_2(\mu\text{-}O_2CC_6F_{13})_4]$ (6).

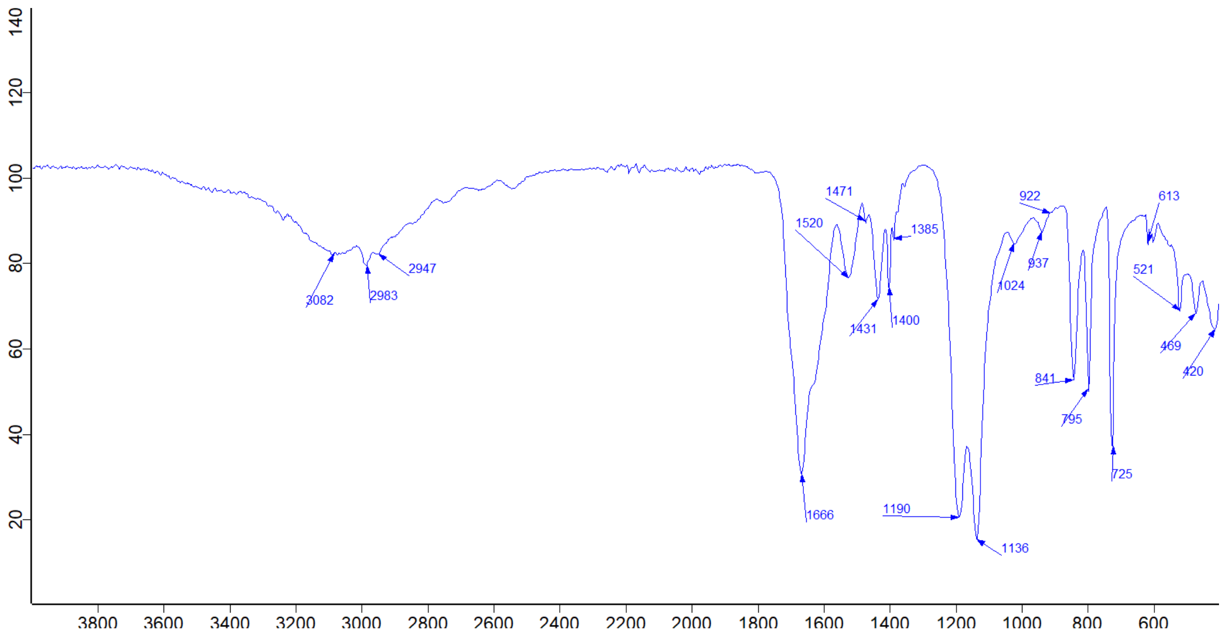

Figure S7. ATR-IR spectrum of $[Cu_2(^iPrNH_2)_2(\mu\text{-}O_2CCF_3)_4]$ (7).

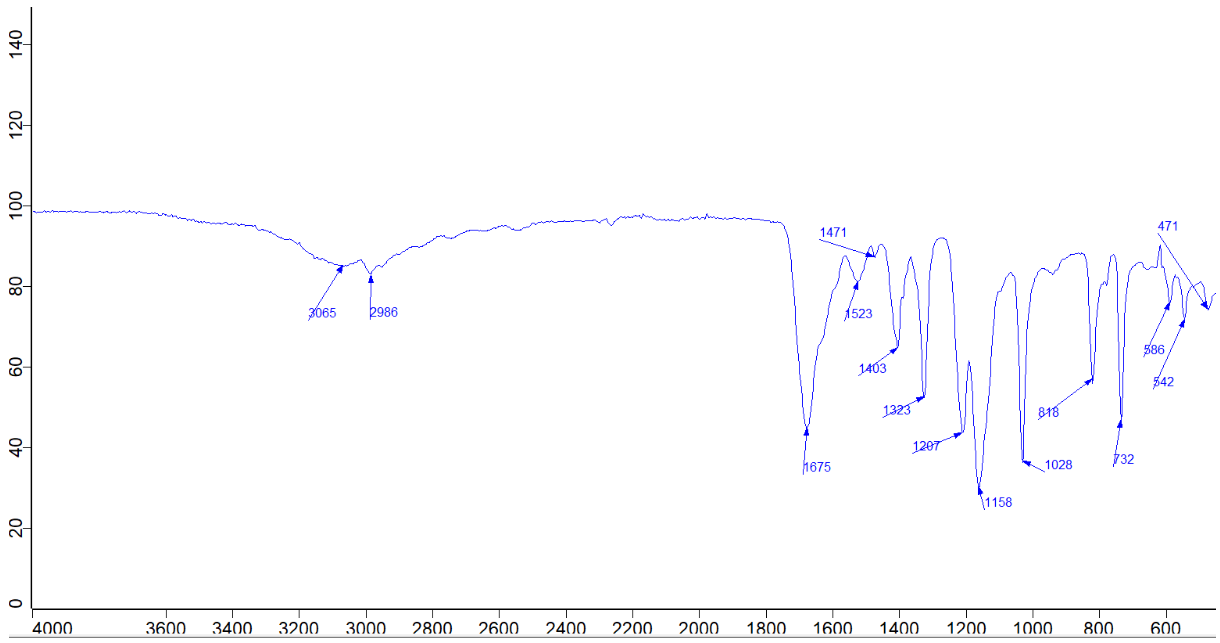

Figure S8. ATR-IR spectrum of $[Cu_2(^iPrNH_2)_2(\mu\text{-}O_2CC_2F_5)_4]$ (8).

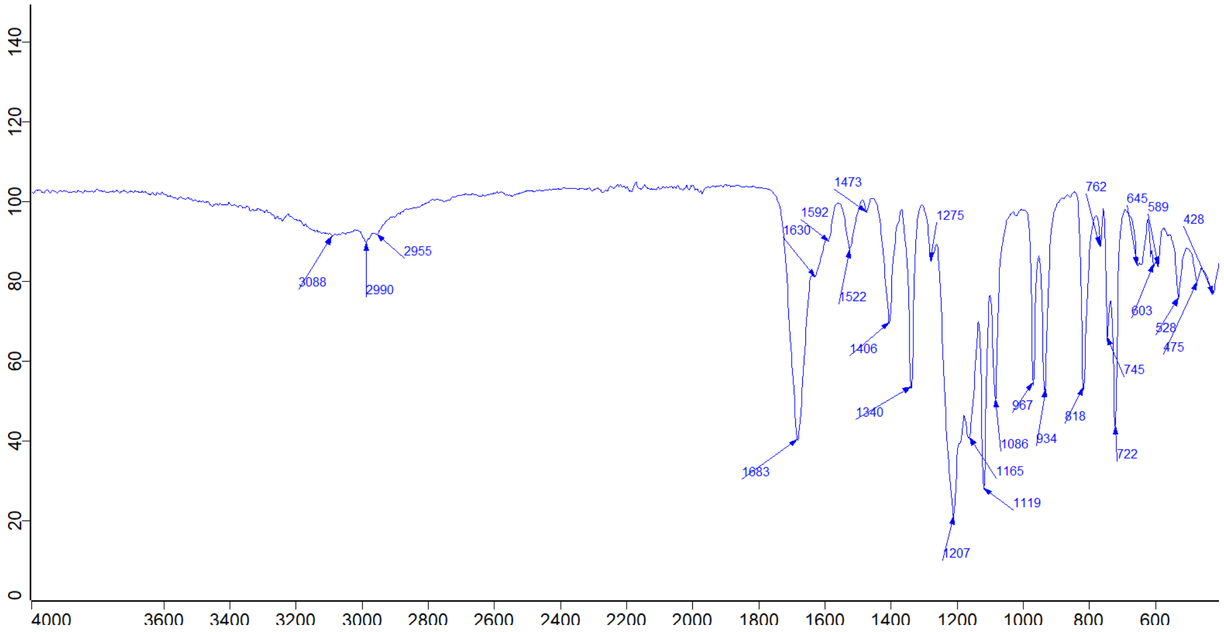

Figure S9. ATR-IR spectrum of $[Cu_2(^iPrNH_2)_2(\mu\text{-}O_2CC_3F_7)_4]$ (9).

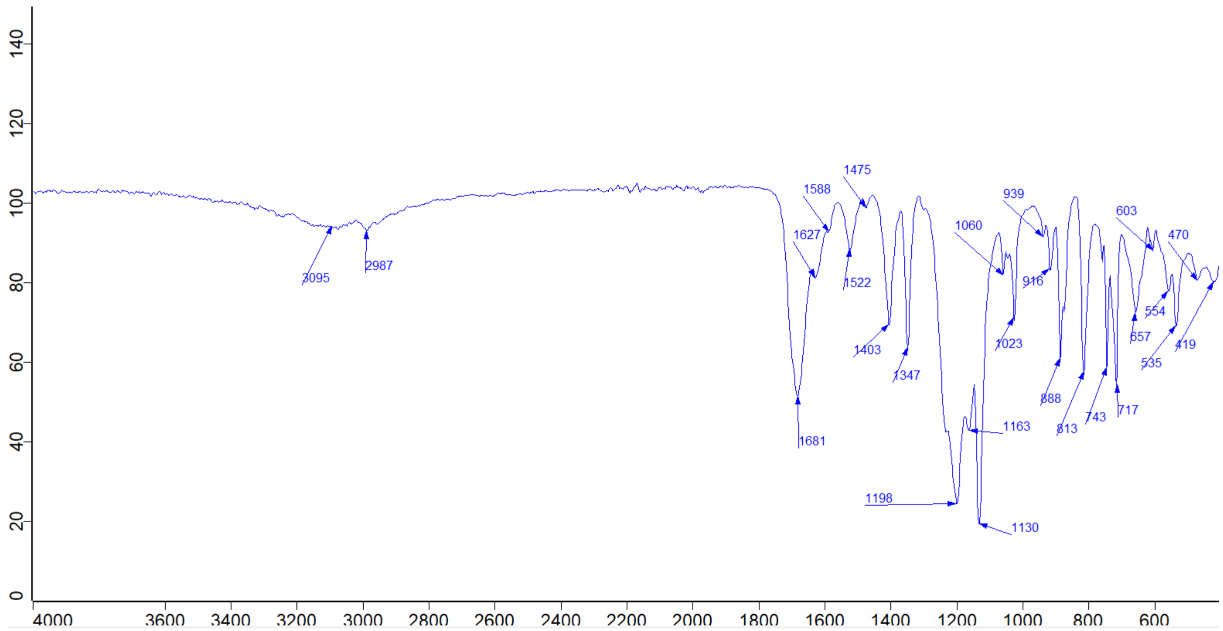

Figure S10. ATR-IR spectrum of $[Cu_2(^iPrNH_2)_2(\mu\text{-}O_2CC_3F_7)_4]$ (10).

Table S1. Mass spectrometry EI MS data of $[Cu_2(EtNH_2)_2(\mu\text{-}O_2CC_2F_5)_4]$ (2).

**Fragments**	***m/z***	**RI [%]**			
		**310 K**	**331 K**	**357 K**	**542 K**
$[C_2H_4N]^+$	42	3	1	2	-
$[CO_2]^+$	44	10	3	6	27
$[C_2H_7N]^{+\cdot}/[COOH]^{+\cdot}$	45	25	5	23	23
$[CF_2]^+$	50	6	1	6	5
$[CF_3]^+$	69	34	11	38	32
$[CF_3CO]^+$	97	12	4	12	9
$[C_2F_4]^+$	100	33	11	33	30
$[C_2F_5]^+$	119	41	21	45	35
$[Cu]^+$	63	14	6	11	12
$[Cu(O_2C)]^+$	107	6	5	4	2
$[Cu(EtNH_2)]^+$	108	4	3	3	-
$[Cu_2F]^+$	145	16	13	16	17
$[Cu_2(O_2CC_2F_5)]^+$	289	100	100	100	100
$[Cu_2(EtNH_2)(O_2CC_2F_5)]^+$	334	2	3	2	-
$[Cu_2(O_2CC_2F_5)_2]^{+\cdot}$	452	39	40	28	38
$[Cu_2(EtNH_2)(O_2CC_2F_5)_3]^+$	660	3	2	2	-
$[Cu_2(EtNH_2)_2(O_2CC_2F_5)_3]^+$	705	7	4	5	-

Table S2. Mass spectrometry EI MS data of $[Cu_2(EtNH_2)_2(\mu\text{-}O_2CC_3F_7)_4]$ (3).

Fragments	*m/z*	RI [%]			
		357 K	417 K	463 K	542 K
$[C_2H_4N]^+$	42	15	2	1	-
$[CO_2]^+$	44	36	8	7	9
$[C_2H_7N]^{+\cdot}$/$[COOH]^+$	45	38	7	3	4
$[CF_2]^+$	50	3	4	3	3
$[CF_3]^+$	69	45	33	23	23
$[C_2F_4]^+$	100	30	25	22	21
$[C_2F_5]^+$	119	34	12	7	7
$[C_3F_5]^+$	131	77	4	3	6
$[C_3F_7]^+$	169	25	19	8	11
$[Cu]^+$	63	6	17	15	12
$[Cu(O_2C)]^+$	107	2	8	7	2
$[Cu(EtNH_2)]^+$	108	3	3	2	-
$[Cu_2F]^+$	145	12	17	17	17
$[Cu_2(O_2CC_3F_7)]^+$	339	100	100	100	100
$[Cu_2(EtNH_2)(O_2CC_3F_7)]^+$	384	2	2	1	-
$[Cu_2(O_2CC_3F_7)_2]^{+\cdot}$	552	29	28	27	26
$[Cu_2(EtNH_2)(O_2CC_3F_7)_3]^+$	810	1	2	2	-
$[Cu_2(EtNH_2)_2(O_2CC_3F_7)_3]^+$	855	8	3	1	-

Table S3. Mass spectrometry EI MS data of $[Cu_2(EtNH_2)_2(\mu\text{-}O_2CC_4F_9)_4]$ (4).

Fragments	*m/z*	RI [%]			
		305 K	345 K	424 K	590 K
$[C_2H_4N]^+$	42	4	3	1	-
$[CO_2]^+$	44	45	34	20	27
$[C_2H_7N]^{+\cdot}/[COOH]^{+\cdot}$	45	10	10	3	1
$[CF_2]^+$	50	7	5	3	2
$[CF_3]^+$	69	92	73	46	34
$[C_2F_3]^+$	81	4	3	2	2
$[C_3F_3]^+$	93	8	5	4	7
$[CF_3CO]^+$	97	1	1	1	-
$[C_2F_4]^+$	100	84	66	42	18
$[C_2F_5]^+$	119	16	14	9	4
$[C_3F_5]^+$	131	100	80	52	100
$[C_3F_6]^+$	150	12	11	7	4
$[C_4F_7]^+$	181	6	5	3	13
$[C_4F_9]^+$	219	15	15	10	2
$[Cu]^+$	63	6	7	7	-
$[Cu_2]^{+\cdot}$	126	1	1	1	-
$[Cu_2F]^+$	145	9	12	12	-
$[Cu_2(O_2CC_4F_9)]^+$	389	69	100	100	1
$[Cu_2(EtNH_2)(O_2CC_4F_9)]^+$	434	1	1	-	-
$[Cu_2(O_2CC_4F_9)_2]^{+\cdot}$	652	16	25	26	-
$[Cu_2(EtNH_2)(O_2CC_4F_9)_3)]^+$	960	3	3	-	-
$[Cu_2(EtNH_2)_2(O_2CC_4F_9)_3]^+$	1005	1	1	-	-
$[Cu_3(O_2CC_4F_9)_5]^+$	1506	<1	<1	1	-

Table S4. Mass spectrometry EI MS data of $[Cu_2(EtNH_2)_2(\mu\text{-}O_2CC_5F_{11})_4]$ (5).

Fragments	*m/z*	RI [%]		
		**319 K**	**425 K**	**590 K**
$[C_2H_3N]^+$	41	1	-	1
$[C_2H_4N]^+$	42	2	-	-
$[CO_2]^+$	44	11	3	16
$[C_2H_7N]^{+\cdot}/[COOH]^{+\cdot}$	45	28	8	2
$[CF_2]^+$	50	4	2	1
$[CF_3]^+$	69	70	33	30
$[C_2F_3]^+$	81	3	1	2
$[C_3F_3]^+$	93	6	5	11
$[C_2F_4]^+$	100	39	24	7
$[C_2F_5]^+$	119	24	13	5
$[C_3F_5]^+$	131	100	67	100
$[C_3F_6]^+$	150	3	2	1
$[C_3F_7]^+$	169	6	3	3
$[C_4F_7]^+$	181	24	17	47
$[C_4F_9]^+$	219	3	2	1
$[C_5F_9]^+$	231	5	4	8
$[C_5F_{11}]^+$	269	14	8	-
$[Cu]^+$	63	6	3	-
$[Cu_2]^{+\cdot}$	126	1	1	-
$[Cu_2F]^+$	145	9	8	-
$[Cu_2(O_2CC_5F_{11})]^+$	439	94	100	-
$[Cu_2(EtNH_2)(O_2CC_5F_{11})]^+$	484	2	-	-
$[Cu_2(O_2CC_5F_{11})_2]^{+\cdot}$	752	22	21	-
$[Cu_2(EtNH_2)(O_2CC_5F_{11})_3]^+$	1110	4	1	-
$[Cu_2(EtNH_2)_2(O_2CC_5F_{11})_3]^+$	1155	2	-	-
$[Cu_3(O_2CC_5F_{11})_5]^+$	1756	-	2	-

Table S5. Mass spectrometry EI MS data of [$Cu_2(EtNH_2)_2(\mu\text{-}O_2CC_6F_{13})_4$] (6).

Fragments	*m/z*	RI [%]			
		305 K	398 K	431 K	616 K
[C_2H_4N]$^+$	42	9	2	1	-
[CO_2]$^+$	44	41	21	13	18
[C_2H_7N]$^{+\cdot}$/[COOH]$^{+\cdot}$	45	25	8	4	-
[CF_2]$^+$	50	4	4	4	1
[CF_3]$^+$	69	81	78	65	34
[C_2F_3]$^+$	81	3	4	3	1
[C_3F_3]$^+$	93	9	8	8	8
[C_2F_4]$^+$	100	39	56	55	10
[C_2F_5]$^+$	119	23	29	25	10
[C_3F_5]$^+$	131	100	96	81	100
[C_3F_6]$^+$	150	3	5	4	1
[C_3F_7]$^+$	169	9	14	12	2
[C_4F_7]$^+$	181	20	22	19	26
[C_5F_7]$^+$	193	1	2	2	2
[C_5F_9]$^+$	231	12	19	17	16
[C_6F_{13}]$^+$	319	3	9	10	-
[Cu]$^+$	63	3	6	6	-
[$Cu(O_2C)$]$^+$	107	1	3	4	-
[$Cu(EtNH_2)$]$^+$	108	1	2	1	-
[Cu_2F]$^+$	145	5	11	10	-
[$Cu_2(O_2CC_6F_{13})$]$^+$	489	34	100	100	-
[$Cu_2(EtNH_2)(O_2CC_6F_{13})$]$^+$	534	-	2	1	-
[$Cu_2(O_2CC_6F_{13})_2$]$^{+\cdot}$	852	4	18	20	-
[$Cu_2(EtNH_2)(O_2CC_6F_{13})_3$]$^+$	1260	-	2	2	-
[$Cu_2(EtNH_2)_2(O_2CC_6F_{13})_3$]$^+$	1305	1	1	-	-

Table S6. Mass spectrometry EI MS data of $[Cu_2(^iPrNH_2)_2(\mu\text{-}O_2CC_2F_5)_4]$ (8).

Fragments	*m/z*	RI [%]			
		357 K	404 K	458 K	505 K
$[C_2H_4N]^+$	42	1	1	47	21
$[C_3H_7]^+$	43	86	87	18	6
$[CO_2]^+$	44	7	5	4	80
$[C_2H_7N]^{+\cdot}$/$[COOH]^{+\cdot}$	45	68	41	52	40
$[CF_2]^+$	50	14	-	100	66
$[C_3H_7N]^+$	57	30	14	4	3
$[C_3H_8N]^+$	58	1	1	33	7
$[C_3H_9N]^{+\cdot}$	59	40	46	8	2
$[CF_3]^+$	69	77	4	3	2
$[CF_3CO]^+$	97	26	74	27	19
$[C_2F_4]^+$	100	66	2	-	100
$[C_2F_5]^+$	119	63	3	1	-
$[Cu]^+$	63	3	-	22	16
$[Cu(O_2C)]^+$	107	-	45	4	3
$[Cu(^iPrNH_2)]^+$	122	32	53	10	-
$[Cu_2]^{+\cdot}$	126	1	4	7	4
$[Cu_2F]^+$	145	2	-	19	13
$[Cu(^iPrNH_2)(O_2CC_2F_5)]^+$	285	10	24	7	-
$[Cu_2(O_2CC_2F_5)]^+$	289	14	3	60	38
$[Cu_2(^iPrNH_2)(O_2CC_2F_5)]^+$	348	9	16	2	-
$[Cu_2(O_2CC_2F_5)_2]^{+\cdot}$	452	5	-	16	11
$[Cu_2(^iPrNH_2)(O_2CC_2F_5)_3]^+$	674[a)]	-	-	-	-
$[Cu_2(^iPrNH_2)_2(O_2CC_2F_5)_3]^+$	733	65	72	1	-

a) The $\mathbf{[Cu_2(^iPrNH_2)(O_2CC_2F_5)_3]^+}$ ion (674 m/z) was detected but not at the temperatures selected for Table S6.

Table S7. Mass spectrometry EI MS data of [$Cu_2(^iPrNH_2)_2(\mu\text{-}O_2CC_3F_7)_4$] (9).

Fragments	*m/z*	RI [%]			
		340 K	372 K	499 K	544 K
$[C_2H_4N]^+$	42	67	95	100	10
$[CO_2]^+$	44	2	3	2	14
$[C_2H_7N]^{+\cdot}$/$[COOH]^+$	45	12	19	20	22
$[CF_2]^+$	50	10	5	44	49
$[C_3H_7N]^+$	57	4	6	7	-
$[C_3H_8N]^+$	58	61	100	42	1
$[C_3H_9N]^{+\cdot}$	59	10	16	10	-
$[CF_3]^+$	69	100	-	2	2
$[C_2F_4]^+$	100	48	-	1	1
$[C_2F_5]^+$	119	27	63	98	100
$[C_3F_5]^+$	131	54	59	97	79
$[C_3F_7]^+$	169	10	29	47	50
$[Cu]^+$	63	-	13	26	32
$[Cu(^iPrNH_2)]^+$	122	17	25	11	-
$[Cu_2]^{+\cdot}$	126	-	2	1	5
$[Cu_2F]^+$	145	2	9	20	23
$[Cu(^iPrNH_2)(O_2CC_3F_7)]^+$	335	2	4	3	-
$[Cu_2(O_2CC_3F_7)]^+$	339	5	24	51	54
$[Cu_2(^iPrNH_2)(O_2CC_3F_7)]^+$	398	3	5	1	-
$[Cu_2(O_2CC_3F_7)_2]^{+\cdot}$	552	-	4	10	9
$[Cu_2(^iPrNH_2)(O_2CC_3F_7)_3]^+$	824	-	2	2	-
$[Cu_2(^iPrNH_2)_2(O_2CC_3F_7)_3]^+$	883	16	24	-	-

The **$[Cu_3(O_2CC_3F_7)_5]^+$** ion (1256 m/z) was detected but not at the temperatures selected for Table S7.

Table S8. Mass spectrometry EI MS data of $[Cu_2(^iPrNH_2)_2(\mu\text{-}O_2CC_4F_9)_4]$ (10).

Fragments	*m/z*	RI [%]			
		**369 K**	**388 K**	**401 K**	**490 K**
$[C_2H_4N]^+$	42	4	6	4	14
$[C_3H_7]^+$	43	41	84	71	3
$[CO_2]^+$	44	3	5	5	37
$[C_2H_7N]^{+\cdot}/[COOH]^{+\cdot}$	45	25	42	68	9
$[CF_2]^+$	50	9	12	19	20
$[C_3H_7N]^+$	57	11	20	15	1
$[C_3H_8N]^+$	58	93	-	-	4
$[C_3H_9N]^+$	59	15	21	23	1
$[CF_3]^+$	69	43	1	72	4
$[C_2F_3]^+$	81	4	6	10	8
$[C_3F_3]^+$	93	12	17	27	31
$[CF_3CO]^+$	97	3	6	10	7
$[C_2F_4]^+$	100	51	100	1	100
$[C_2F_5]^+$	119	27	43	69	44
$[C_3F_5]^+$	131	90	1	1	-
$[C_3F_6]^+$	150	7	15	30	13
$[C_4F_7]^+$	181	8	10	17	7
$[C_4F_9]^+$	219	6	13	27	7
$[Cu]^+$	63	4	7	13	3
$[Cu_2]^{+\cdot}$	126	2	5	3	-
$[Cu_2F]^+$	145	2	8	18	2
$[Cu(^iPrNH_2)(O_2CC_4F_9)]^+$	385	2	2	3	-
$[Cu_2(O_2CC_4F_9)]^+$	389	6	29	64	5
$[Cu_2(^iPrNH_2)(O_2CC_4F_9)]^+$	448	3	4	5	-
$[Cu_2(O_2CC_4F_9)_2]^{+\cdot}$	652	1	5	9	1
$[Cu_2(^iPrNH_2)(O_2CC_4F_9)_3)]^+$	974	1	1	2	-
$[Cu_2(^iPrNH_2)_2(O_2CC_4F_9)_3]^+$	1033	18	20	21	-

The $\mathbf{[Cu_3(O_2CC_4F_9)_5]^+}$ ion (1506 m/z) was detected but not at the temperatures selected for Table S8.

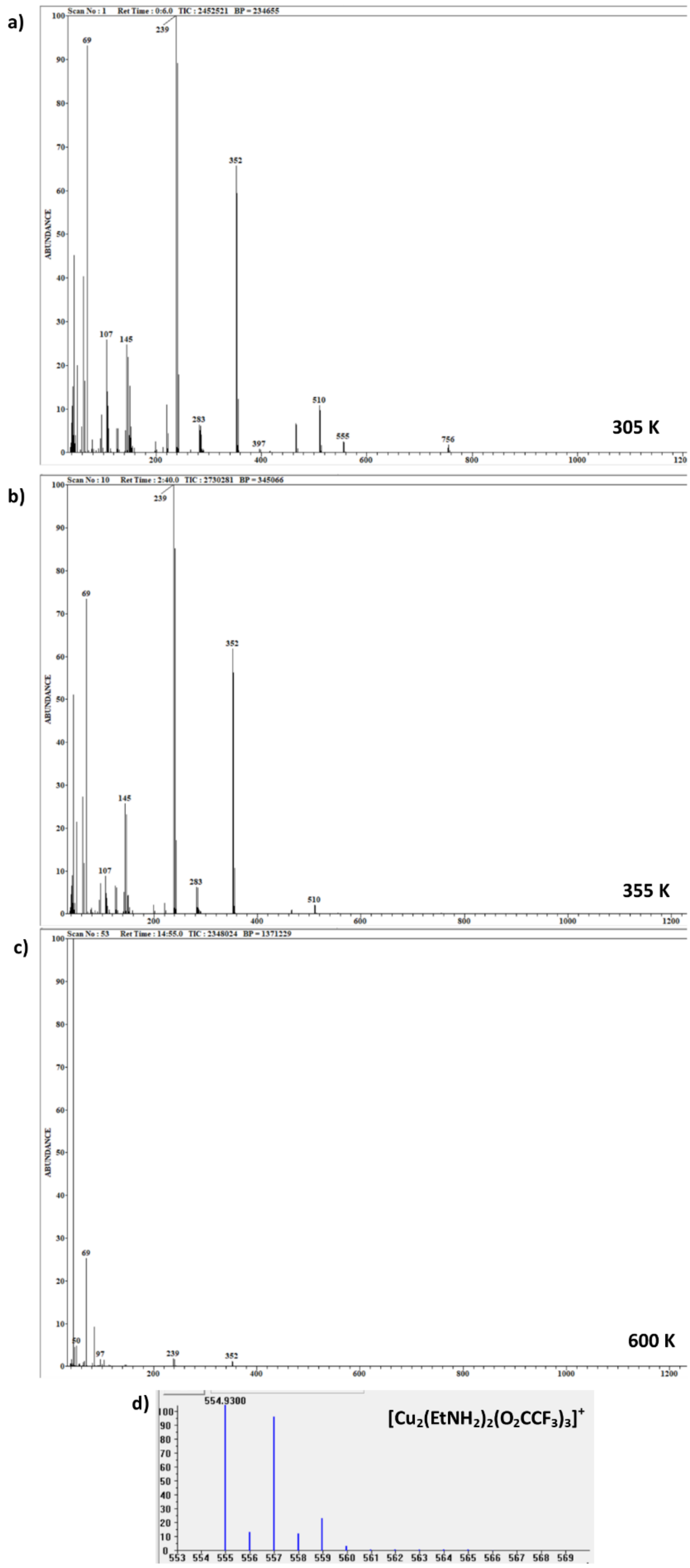

Figure S11. Mass spectrometry EI MS spectra of $[Cu_2(EtNH_2)_2(\mu\text{-}O_2CCF_3)_4]$ (1) at temperature: a) 305 K, b) 355 K, c) 600 K and d) simulation of isotopic pattern for a pseudomolecular ion $[Cu_2(EtNH_2)_2(O_2CCF_3)_3]^+$.

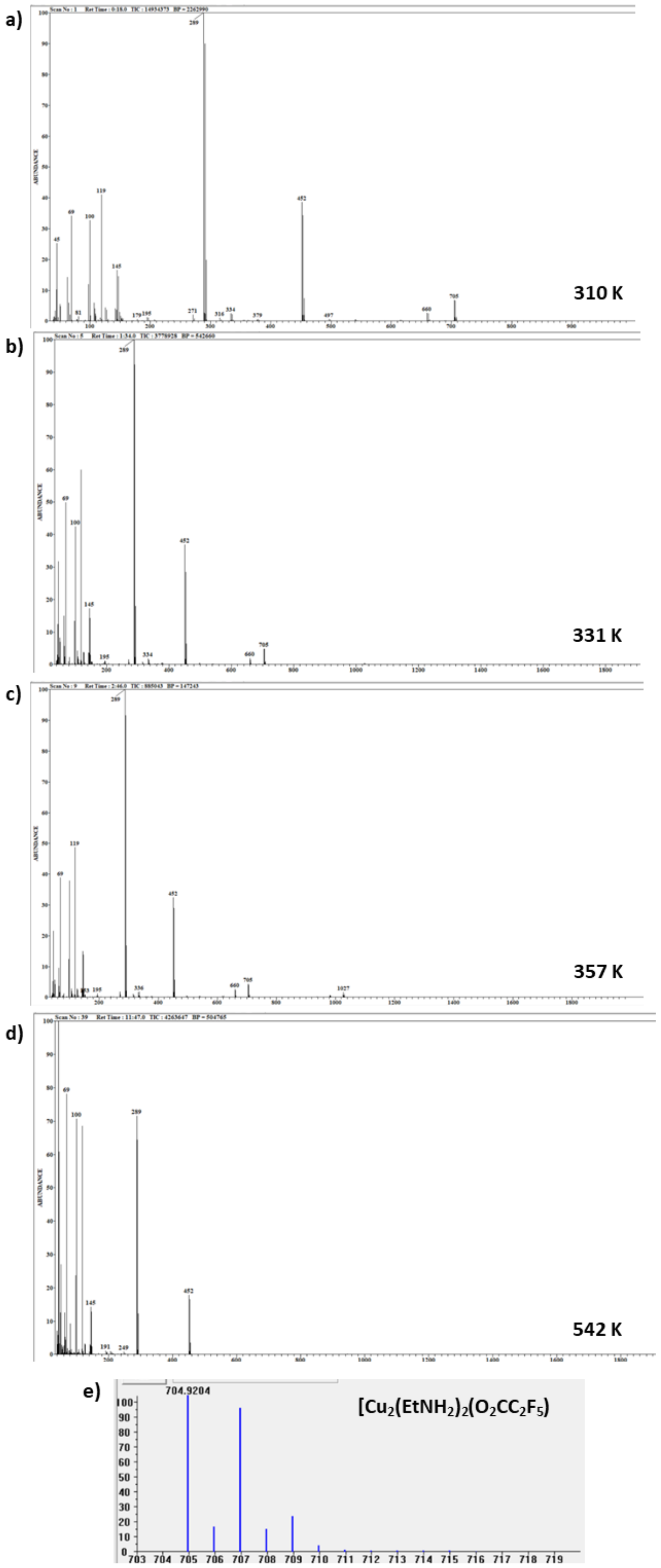

Figure S12. Mass spectrometry EI MS spectra of $[Cu_2(EtNH_2)_2(\mu\text{-}O_2CC_2F_5)_4]$ (2) at temperature: a) 310 K, b) 331 K, c) 357 K d) 542 K and e) simulation of isotopic pattern for a pseudomolecular ion $[Cu_2(EtNH_2)_2(O_2CC_2F_5)_3]^+$.

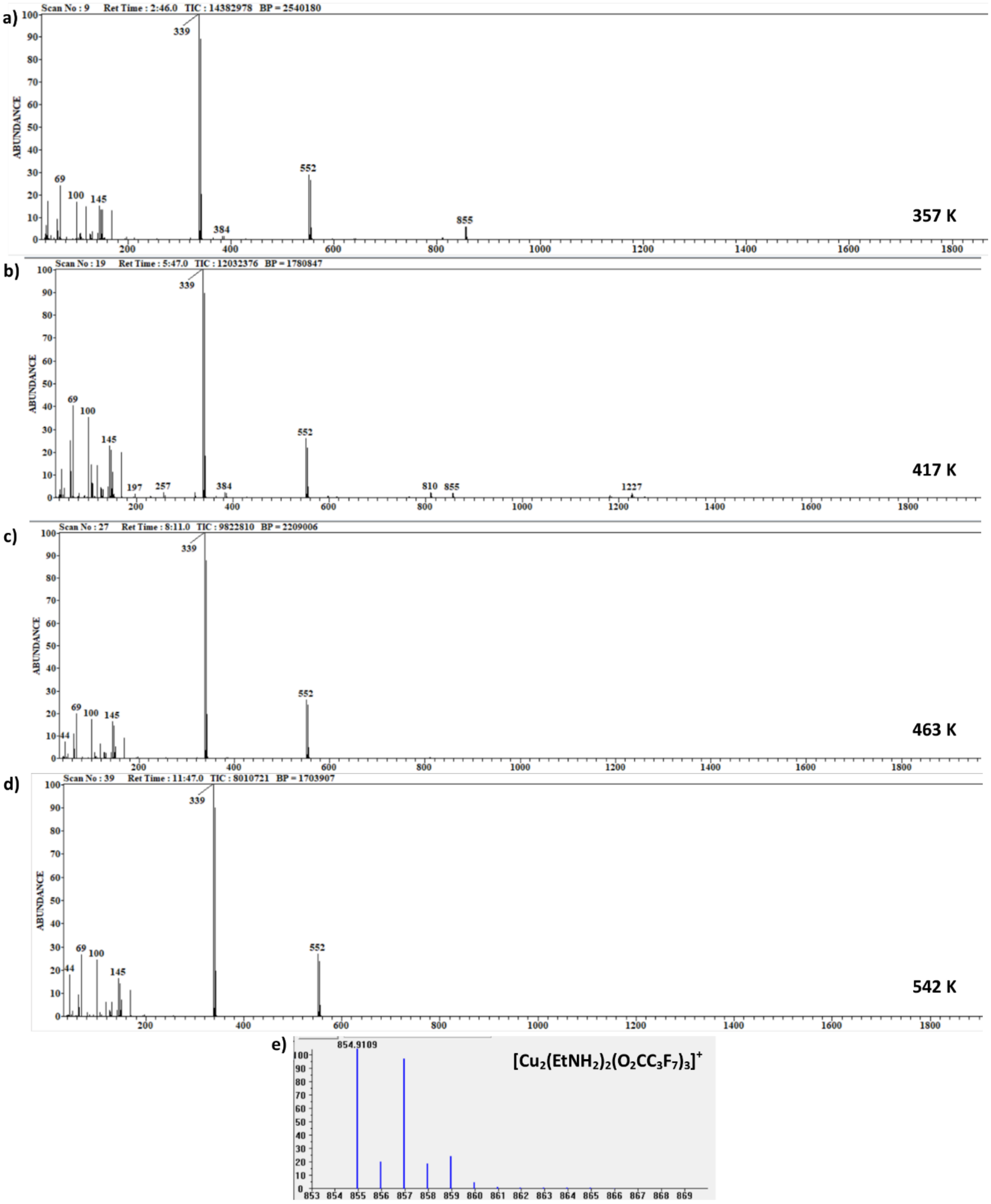

Figure S13. Mass spectrometry EI MS spectra of $[Cu_2(EtNH_2)_2(\mu\text{-}O_2CC_3F_7)_4]$ (3) at temperature: a) 357 K, b) 417 K, c) 463 K d) 542 K, and e) simulation of isotopic pattern for a pseudomolecular ion $[Cu_2(EtNH_2)_2(O_2CC_3F_7)_3]^+$.

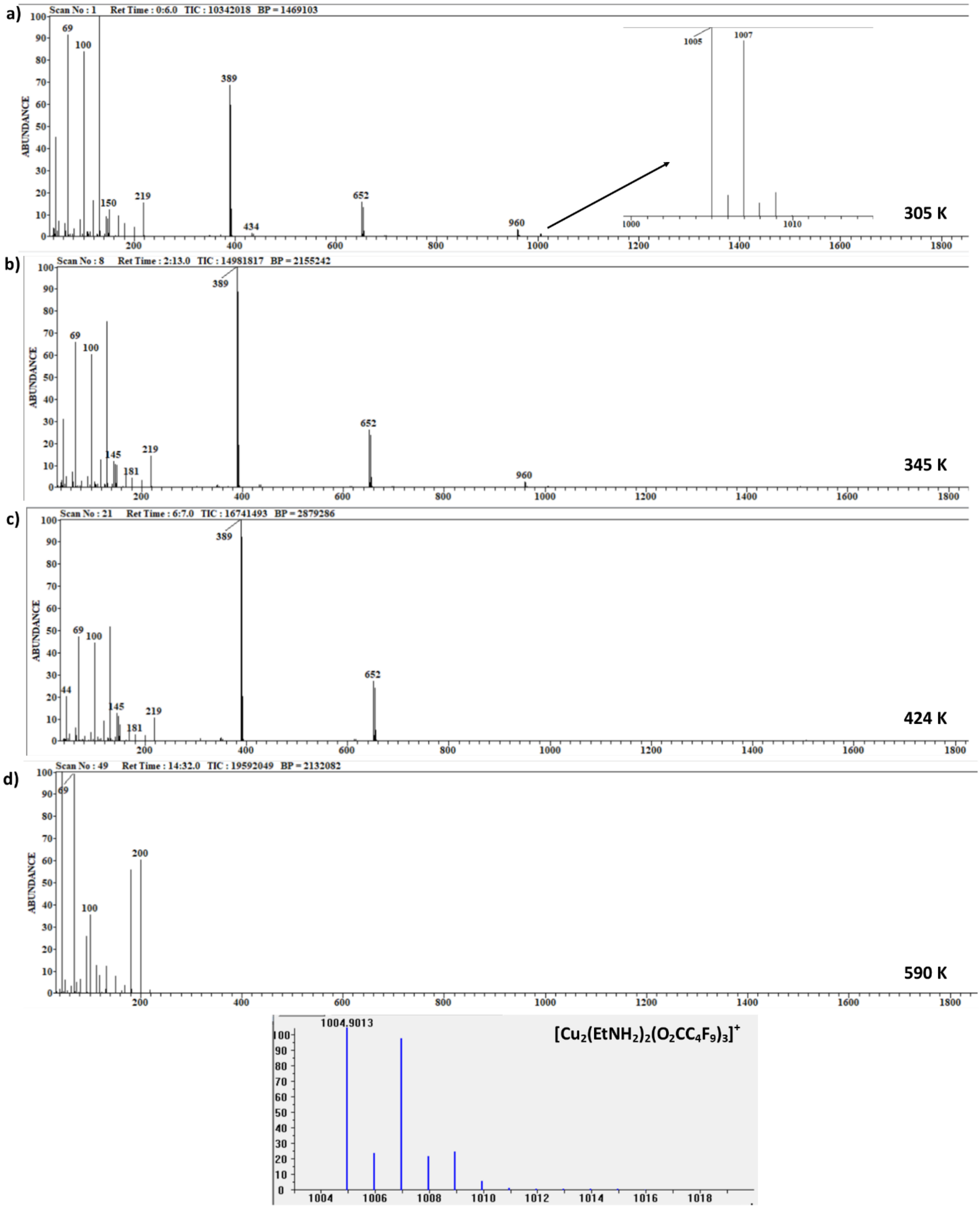

Figure S14. Mass spectrometry EI MS spectra of [$Cu_2(EtNH_2)_2(\mu\text{-}O_2CC_4F_9)_4$] (4) at temperature: a) 305 K, b) 345 K, c) 424 K d) 590 K, and e) simulation of isotopic pattern for a pseudomolecular ion [$Cu_2(EtNH_2)_2(O_2CC_4F_9)_3$]$^+$.

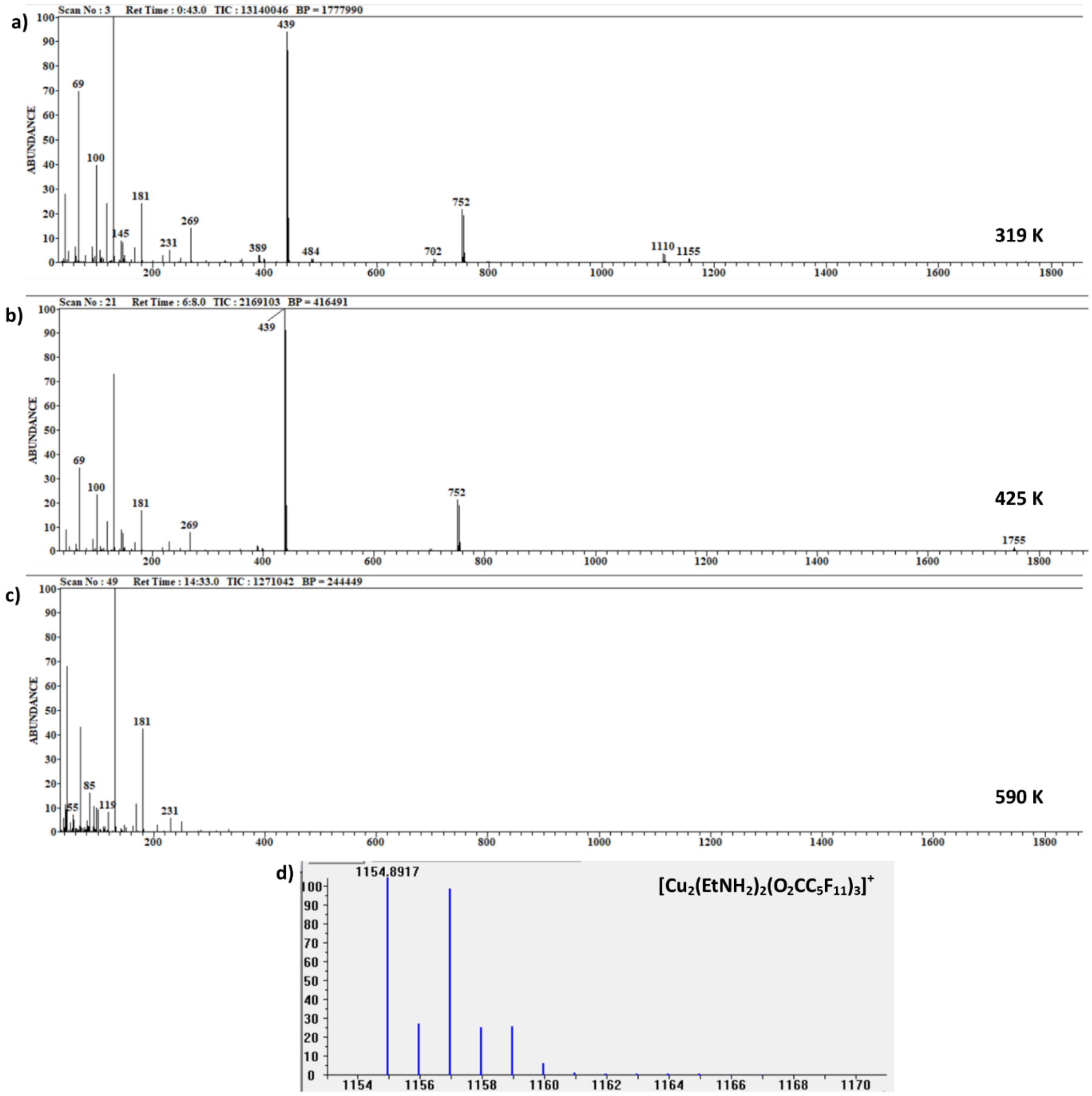

Figure S15. Mass spectrometry EI MS spectra of $[Cu_2(EtNH_2)_2(\mu\text{-}O_2CC_5F_{11})_4]$ (5) at temperature: a) 319 K, b) 425 K, c) 590 K, and d) simulation of isotopic pattern for a pseudomolecular ion $[Cu_2(EtNH_2)_2(O_2CC_5F_{11})_3]^+$.

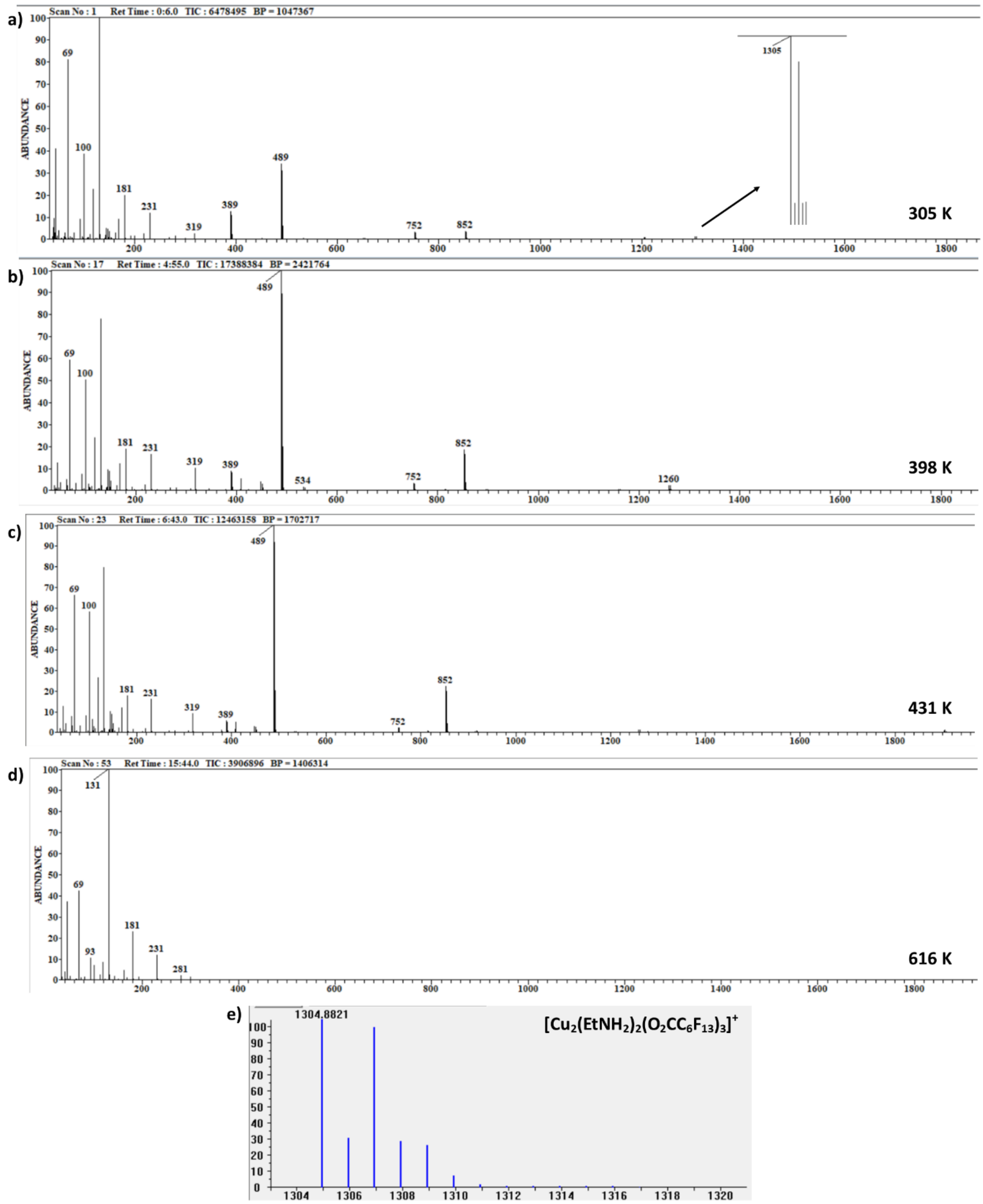

Figure S16. Mass spectrometry EI MS spectra of $[Cu_2(EtNH_2)_2(\mu\text{-}O_2CC_6F_{13})_4]$ (6) at temperature: a) 305 K, b) 398 K, c) 431 K d) 616 K, and e) simulation of isotopic pattern for a pseudomolecular ion $[Cu_2(EtNH_2)_2(O_2CC_6F_{13})_3]^+$.

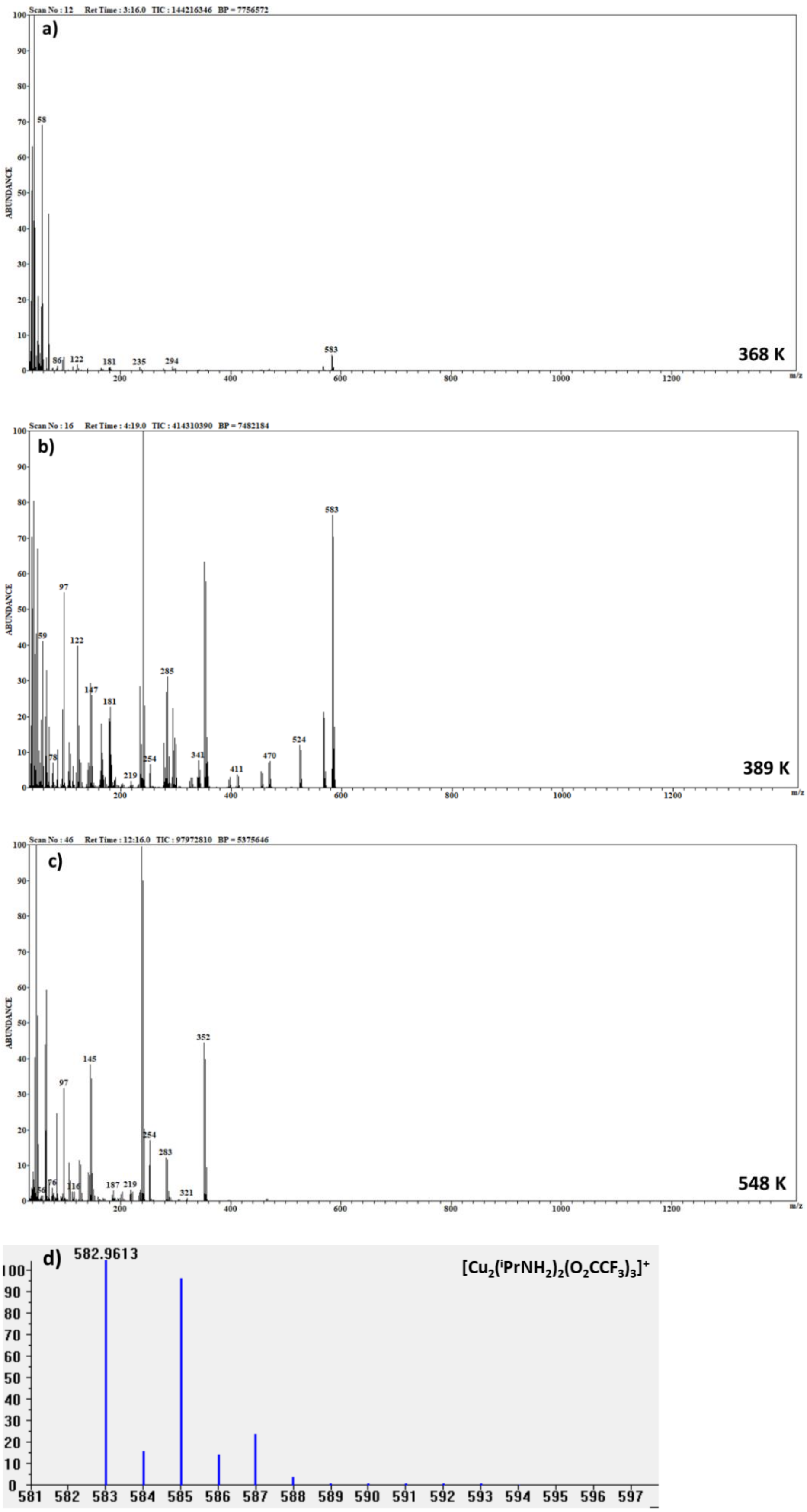

Figure S17. Mass spectrometry EI MS spectra of $[Cu_2(^iPrNH_2)_2(\mu\text{-}O_2CCF_3)_4]$ (7) at temperature: a) 368 K, b) 389 K, c) 548, and d) simulation of isotopic pattern for a pseudomolecular ion $[Cu_2(^iPrNH_2)_2(O_2CCF_3)_3]^+$.

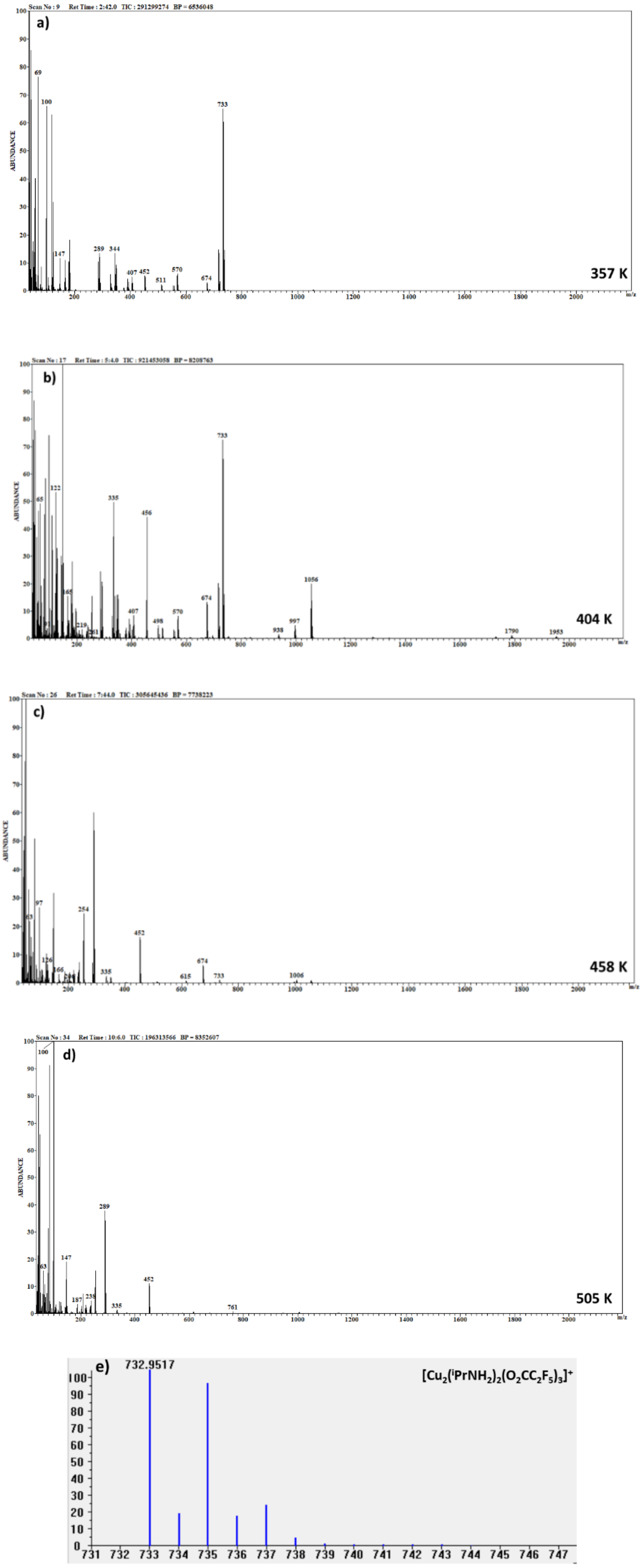

Figure S18. Mass spectrometry EI MS spectra of $[Cu_2(^iPrNH_2)_2(\mu\text{-}O_2CC_2F_5)_4]$ (8) at temperature: a) 357 K, b) 404 K, c) 458 K, d) 505 K, and e) simulation of isotopic pattern for a pseudomolecular ion $[Cu_2(^iPrNH_2)_2(O_2CC_2F_5)_3]^+$.

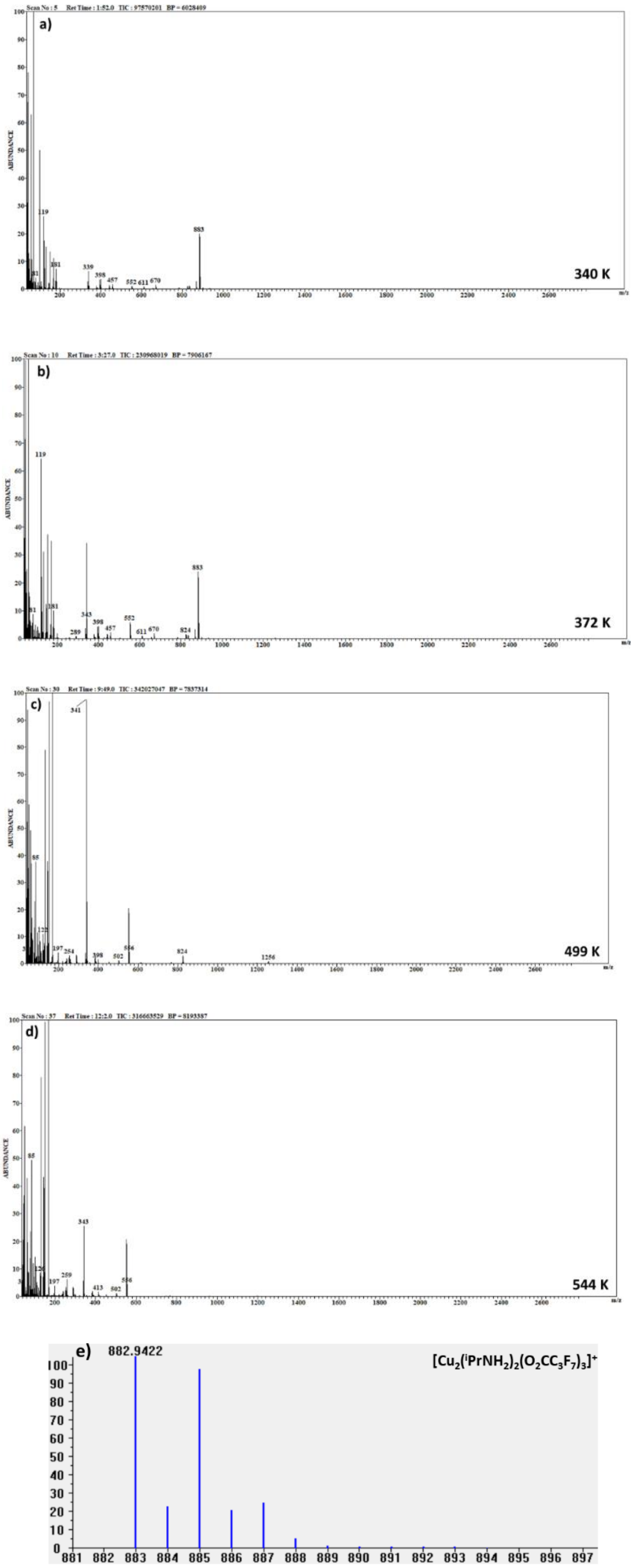

Figure S19. Mass spectrometry EI MS spectra of $[Cu_2(^iPrNH_2)_2(\mu\text{-}O_2CC_3F_7)_4]$ (9) at temperature: a) 340 K, b) 372 K, c) 499 K, d) 544 K, and e) simulation of isotopic pattern for a pseudomolecular ion $[Cu_2(^iPrNH_2)_2(O_2CC_3F_7)_3]^+$.

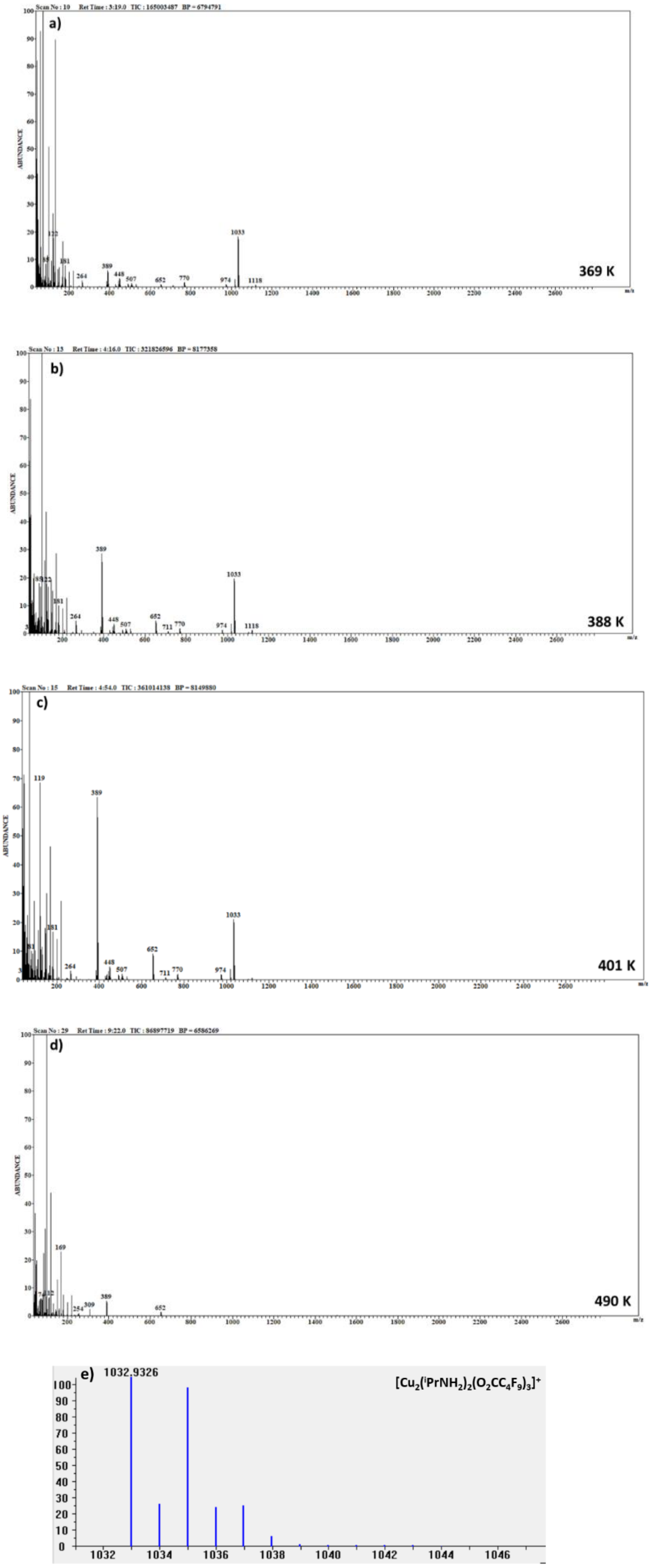

Figure S20. Mass spectrometry EI MS spectra of [$Cu_2(^iPrNH_2)_2(\mu\text{-}O_2CC_4F_9)_4$] (10) a) 369 K, b) 388 K, c) 401 K, d) 490 K, and e) simulation of isotopic pattern for a pseudomolecular ion [$Cu_2(^iPrNH_2)_2(O_2CC_4F_9)_3$]$^+$.

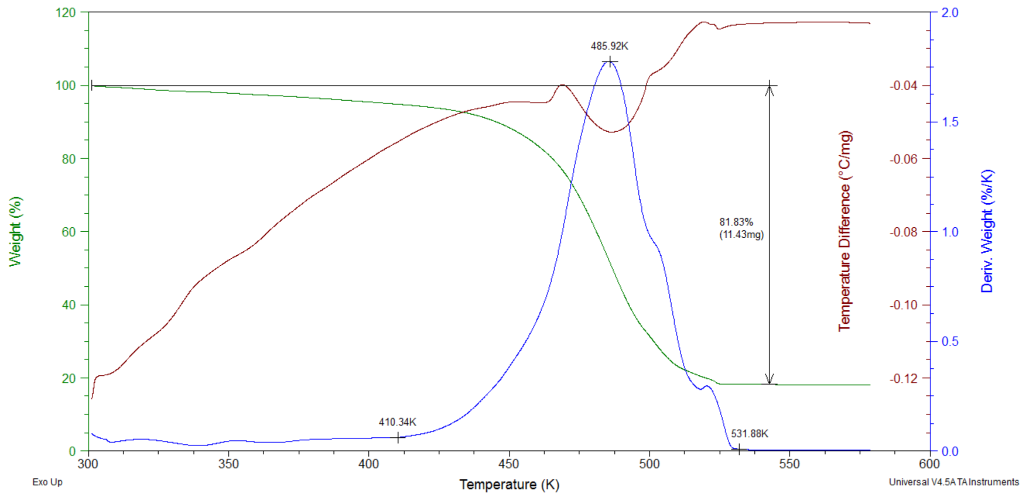

Figure S21. Thermal decomposition of $[Cu_2(EtNH_2)_2(\mu\text{-}O_2CCF_3)_4]$ (1) (TG, DTG, DTA curves).

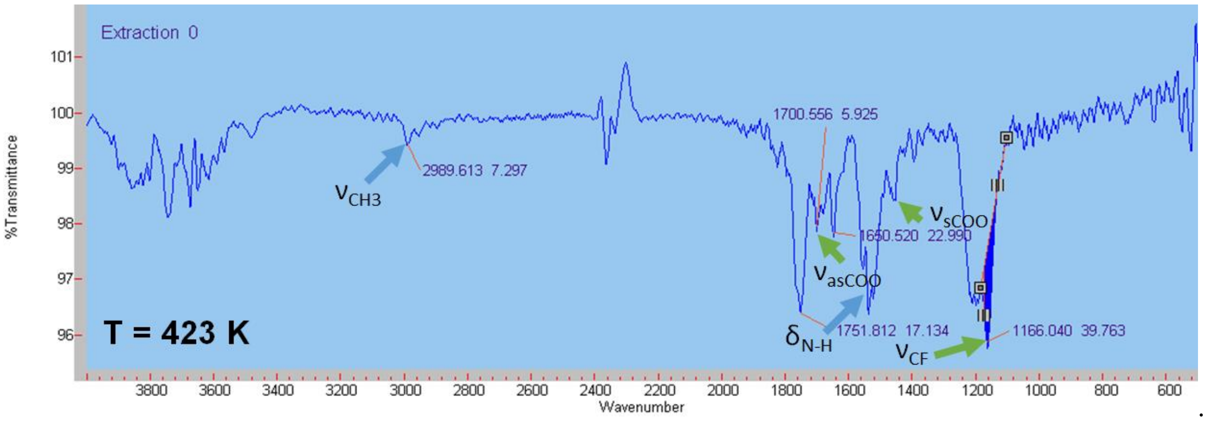

Figure S22. TGA/IR spectrum of $[Cu_2(EtNH_2)_2(\mu\text{-}O_2CCF_3)_4]$ (1) registered at temperature 423 K.

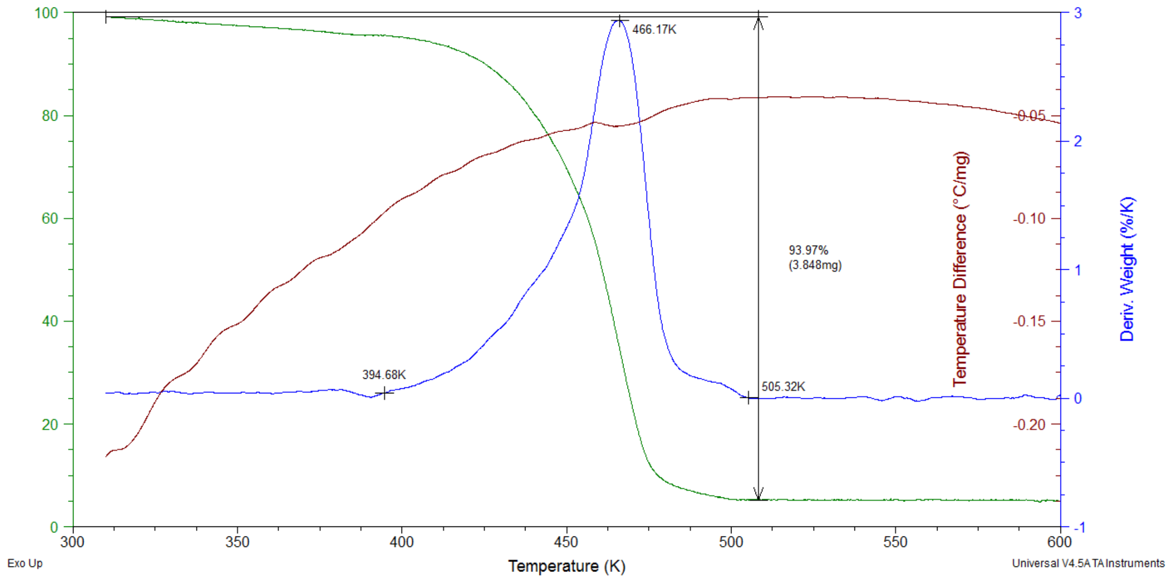

Figure S23. Thermal decomposition of $[Cu_2(EtNH_2)_2(\mu\text{-}O_2CC_3F_7)_4]$ (3) (TG, DTG, DTA curves).

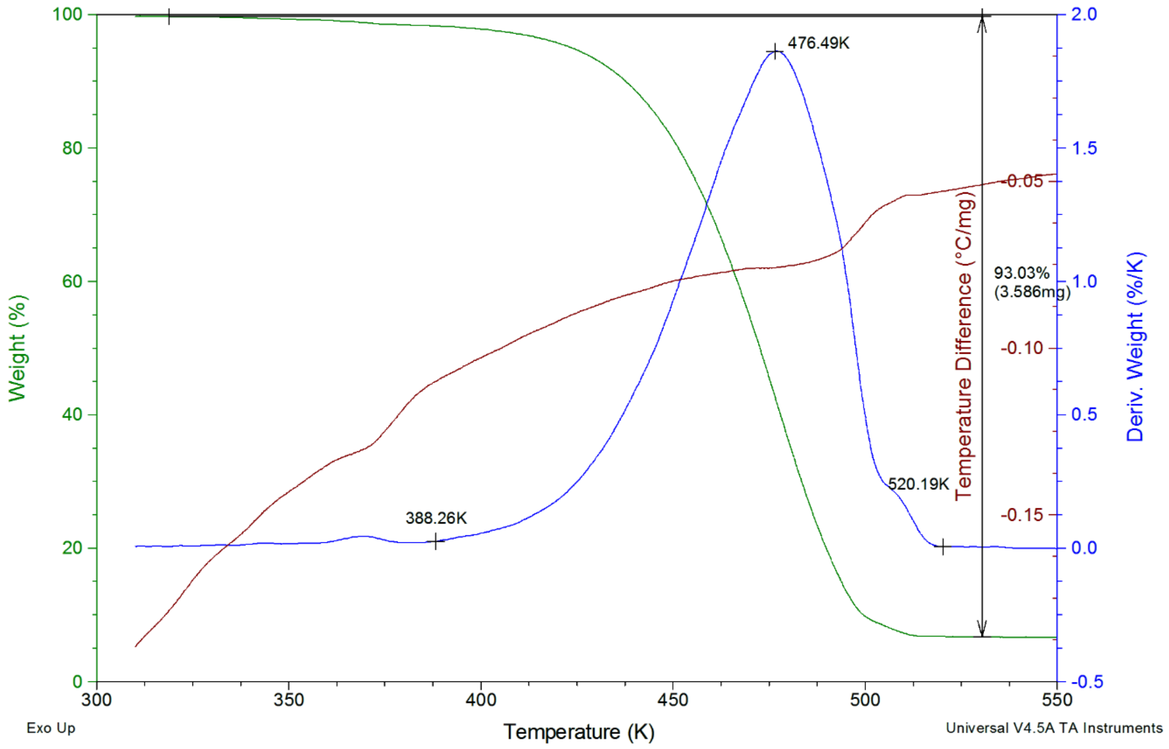

Figure S24. Thermal decomposition of [$Cu_2(EtNH_2)_2(\mu\text{-}O_2CC_4F_9)_4$] (4) (TG, DTG, DTA curves).

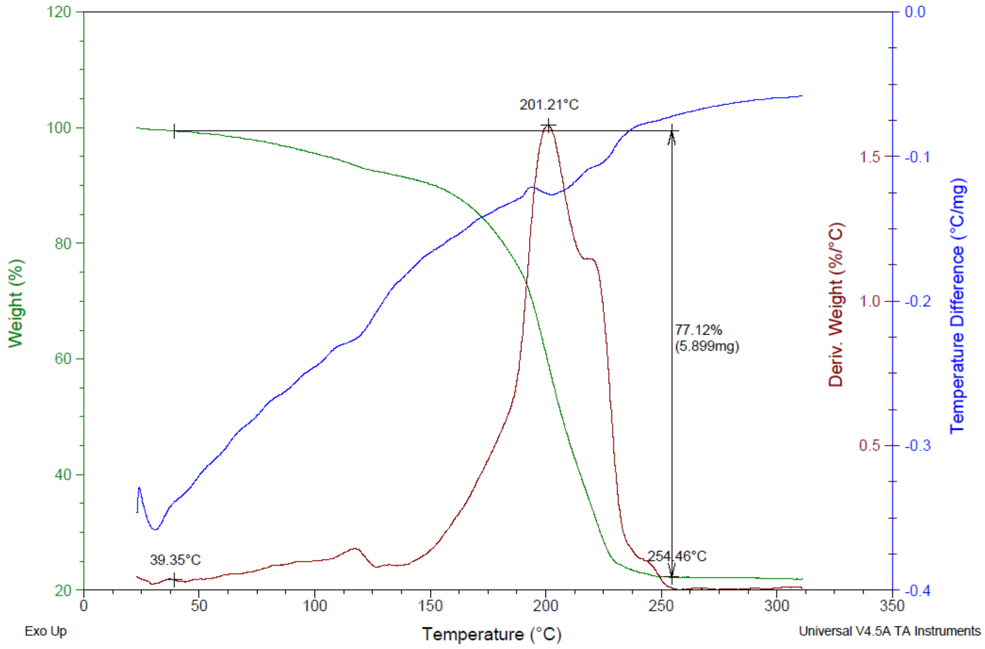

Figure S25. Thermal decomposition of [$Cu_2(^iPrNH_2)_2(\mu\text{-}O_2CCF_3)_4$] (7).

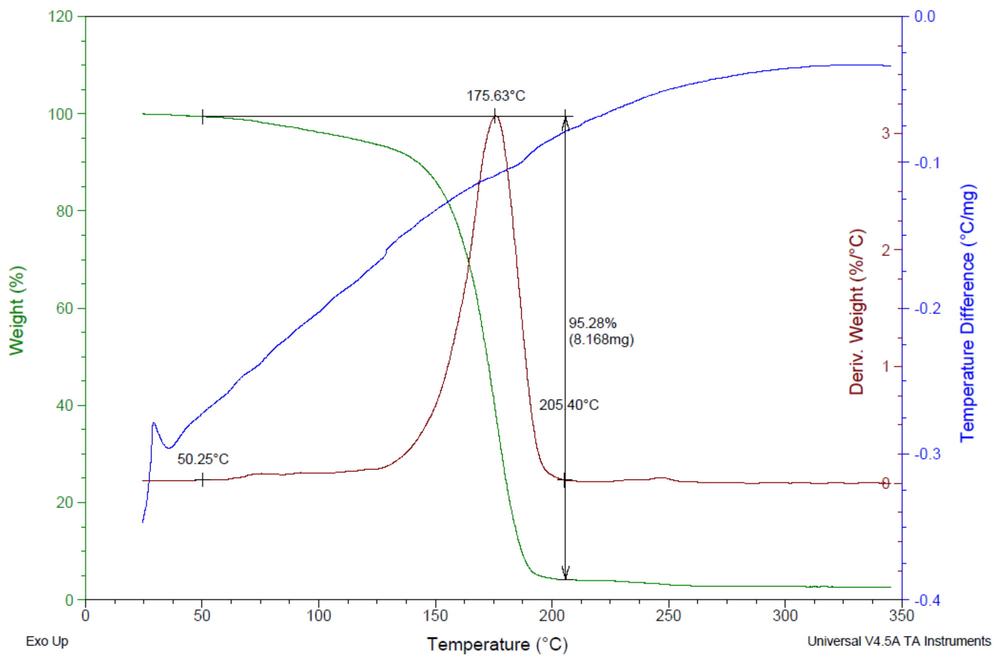

Figure S26. Thermal decomposition of [$Cu_2(^iPrNH_2)_2(\mu\text{-}O_2CC_2F_5)_4$] (8).

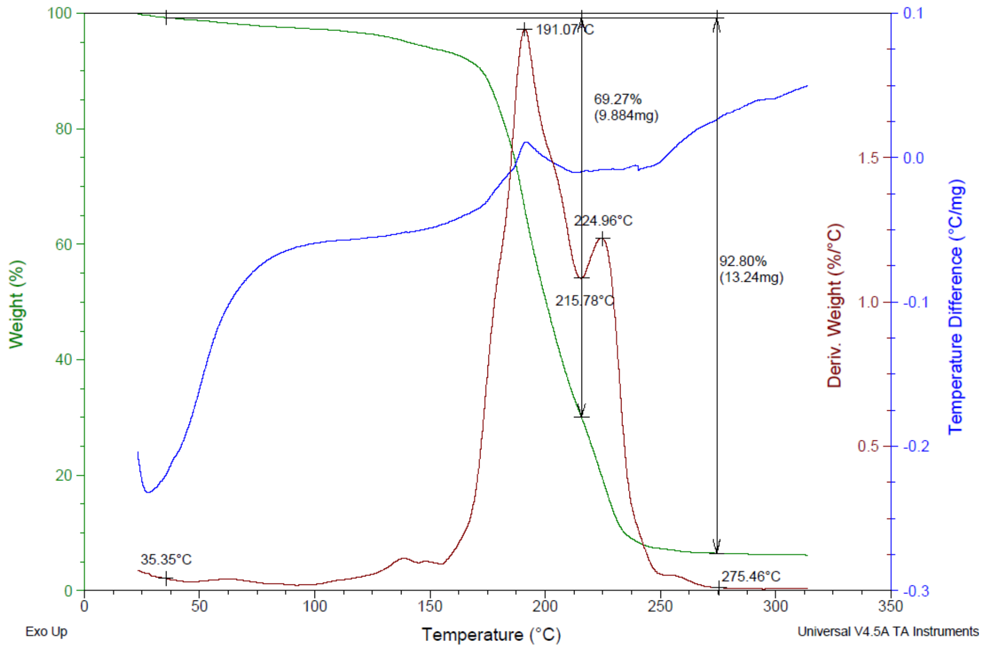

Figure S27. Thermal decomposition of [$Cu_2(^iPrNH_2)_2(\mu\text{-}O_2CC_3F_7)_4$] (9).

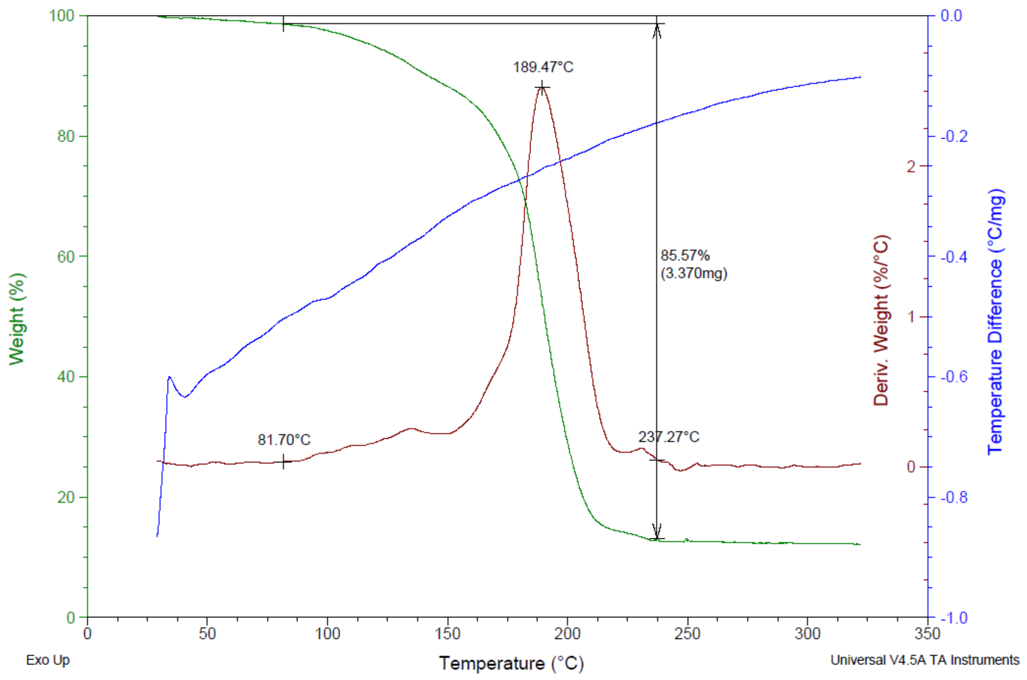

Figure S28. Thermal decomposition of [$Cu_2(^iPrNH_2)_2(\mu\text{-}O_2CC_3F_7)_4$] (10).

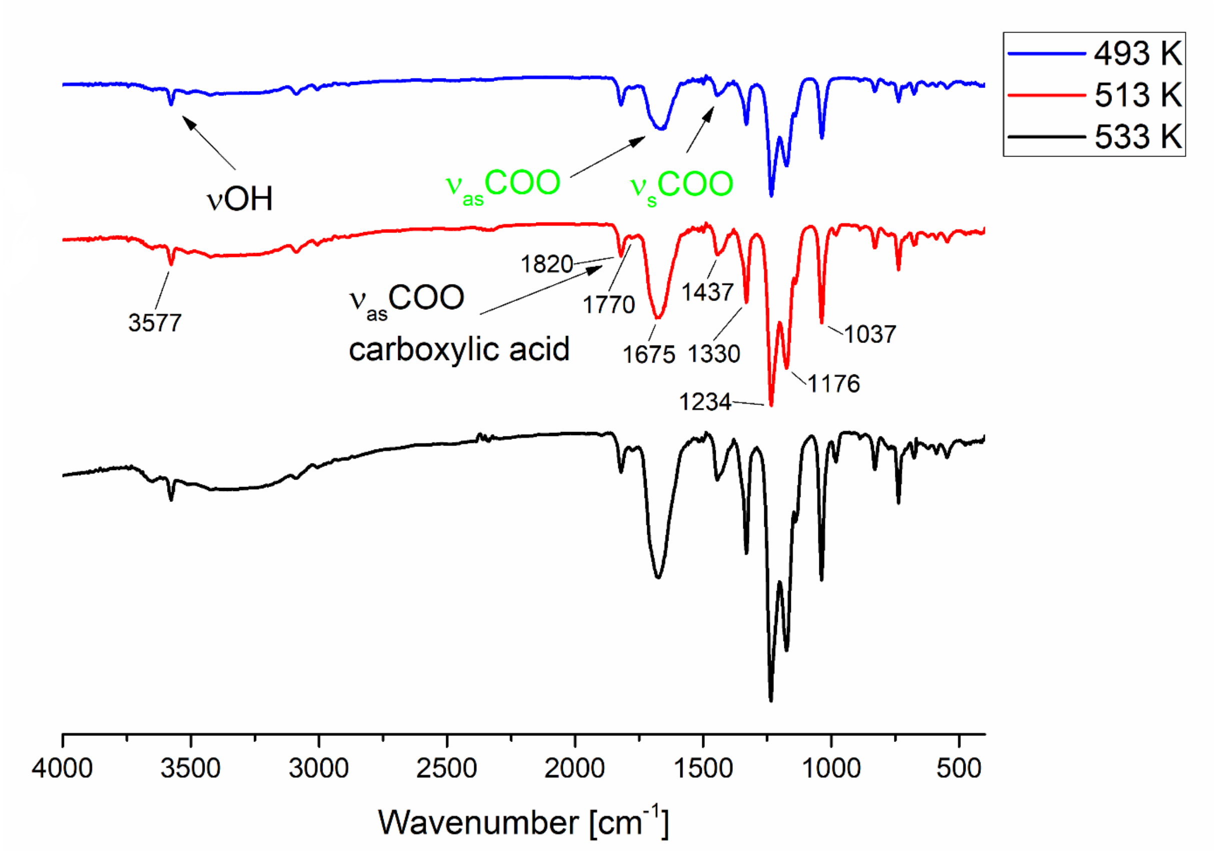

Figure S29. Temperature variable infrared spectra VT IR of vapors formed during the [$Cu_2(\mu\text{-}O_2CC_2F_5)_4$] compound heating.

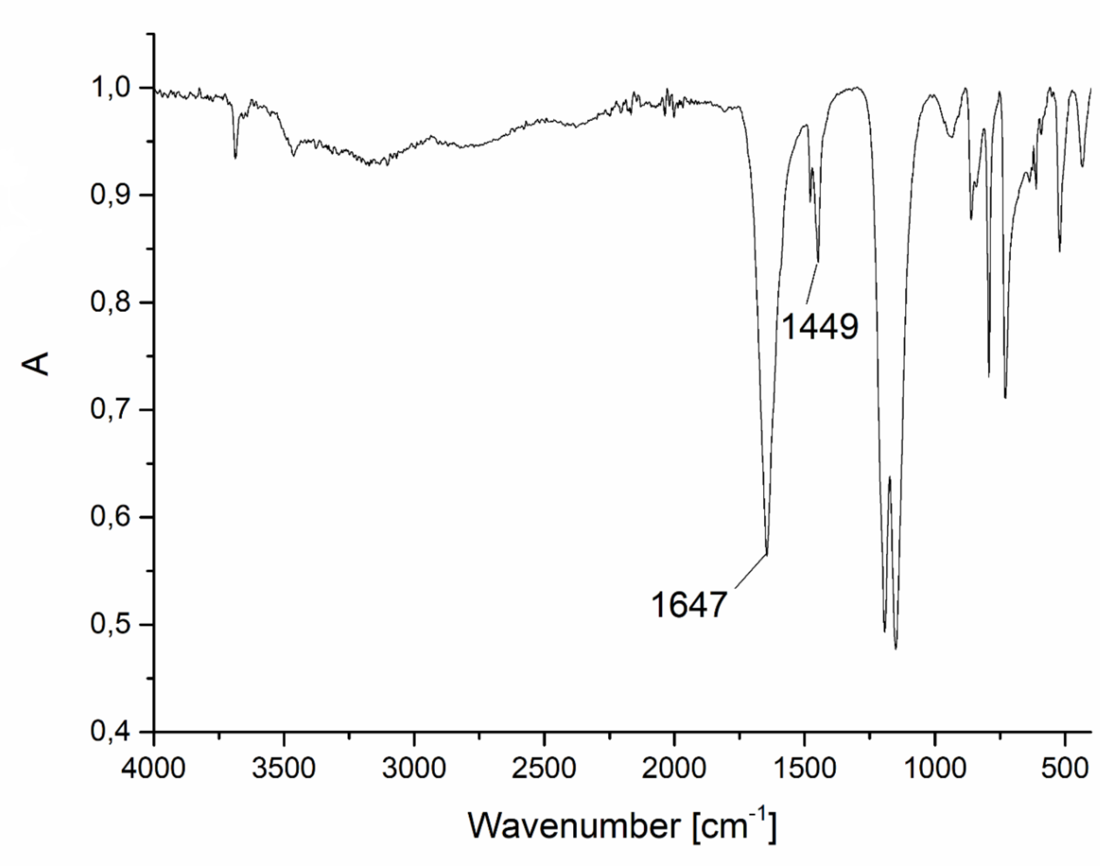

Figure S30. Infrared spectra for the [Cu_2(μ-O_2CCF_3)$_4$] in the solid state.

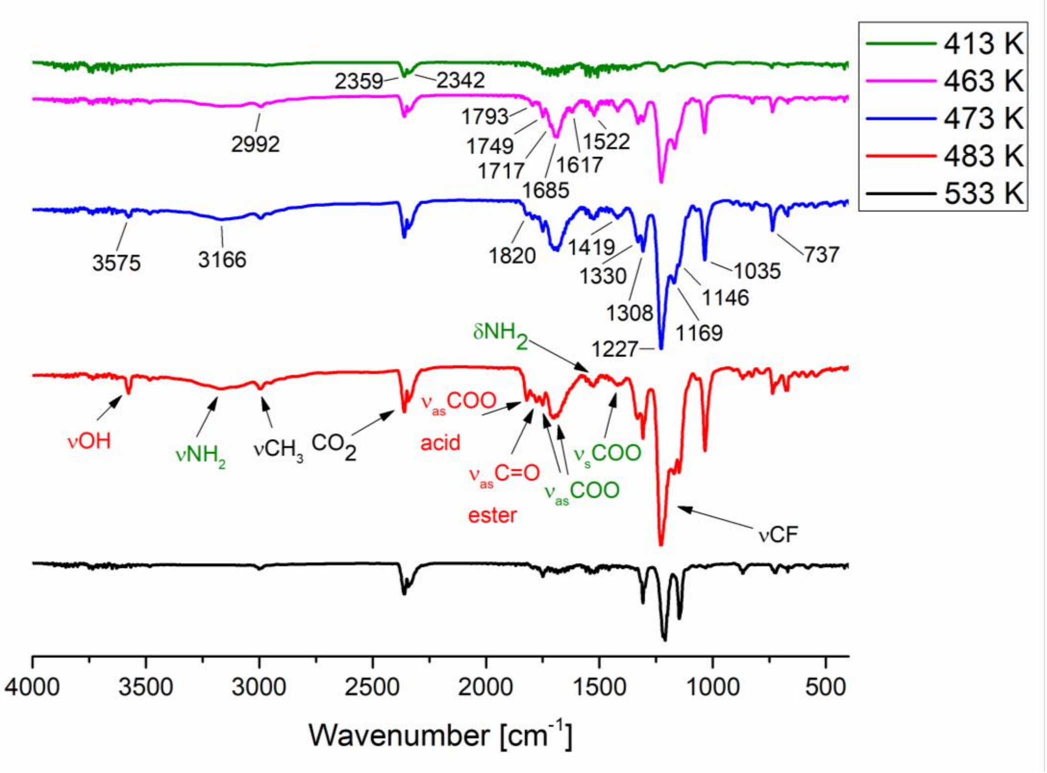

Figure S31. Temperature variable infrared spectra VT IR of vapors formed during the [Cu_2($EtNH_2$)$_2$(μ-$O_2CC_2F_5$)$_4$] complex (2) heating.

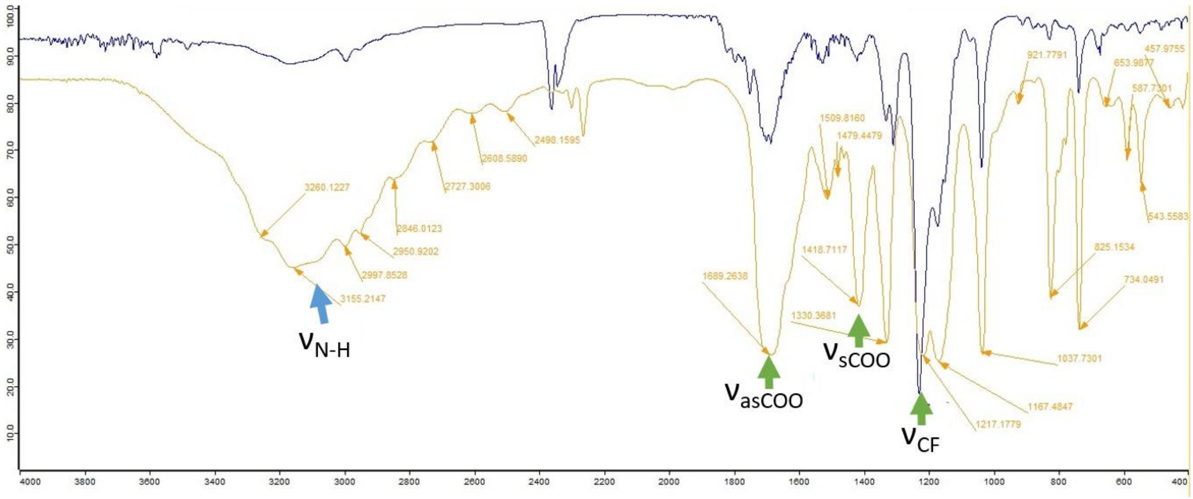

Figure S32. The spectra comparison for the $[Cu_2(EtNH_2)_2(\mu\text{-}O_2CC_2F_5)_4]$ complex (2) in the solid state (orange line) and in the gas phase at 473 K (blue line).

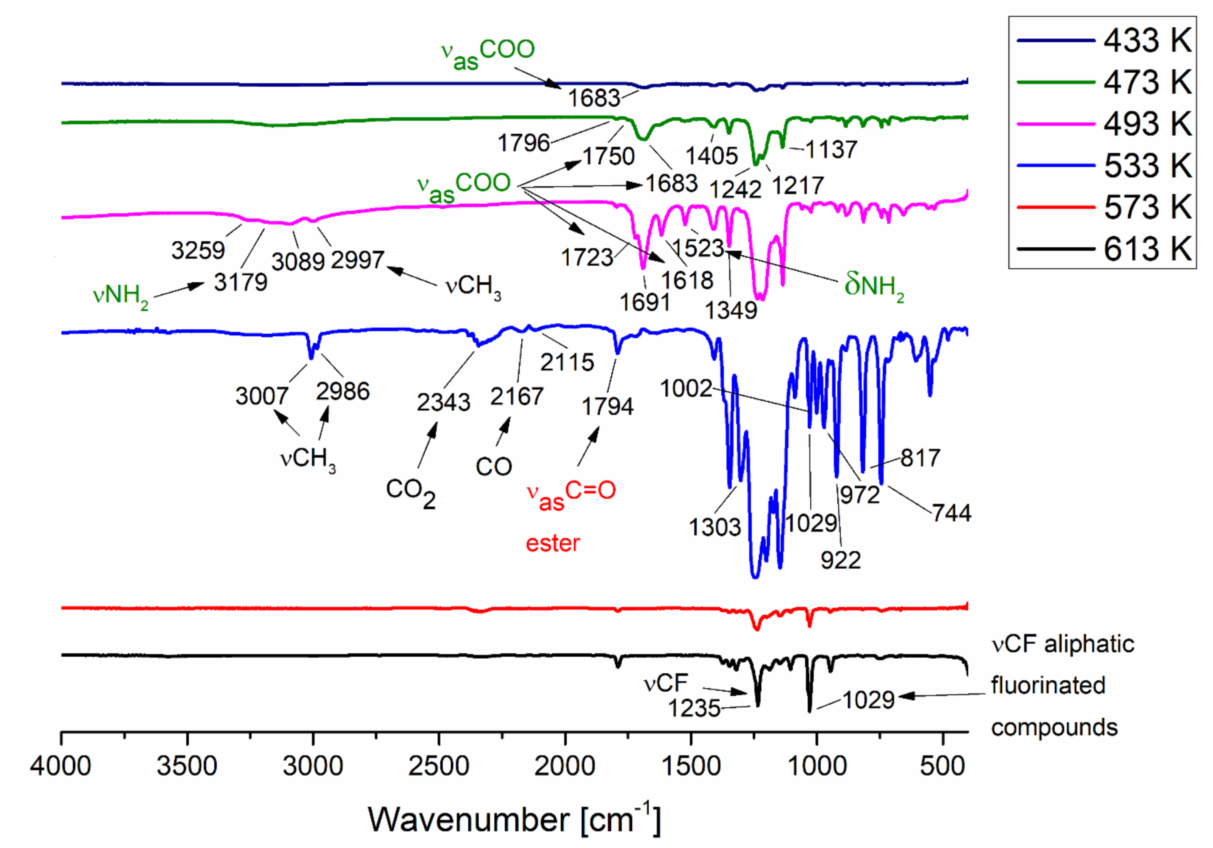

Figure S33. Temperature variable infrared spectra VT IR of vapors formed during the $[Cu_2(EtNH_2)_2(\mu\text{-}O_2CC_4F_9)_4]$ complex (4) heating.

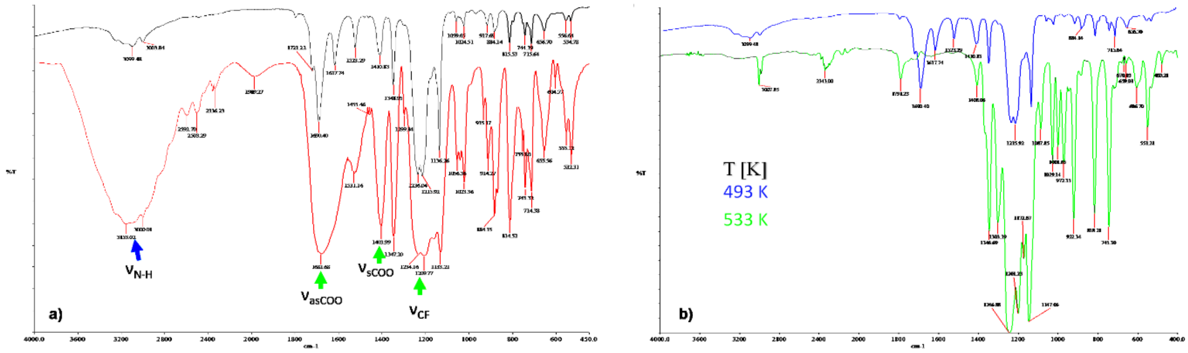

Figure S34. The spectra comparison for the [Cu2(EtNH2)2(μ-O2CC4F9)4] complex (4) a) in the solid state (red line) and in the gas phase at 493 K (black line) b) at temperature 493 K and 533 K.

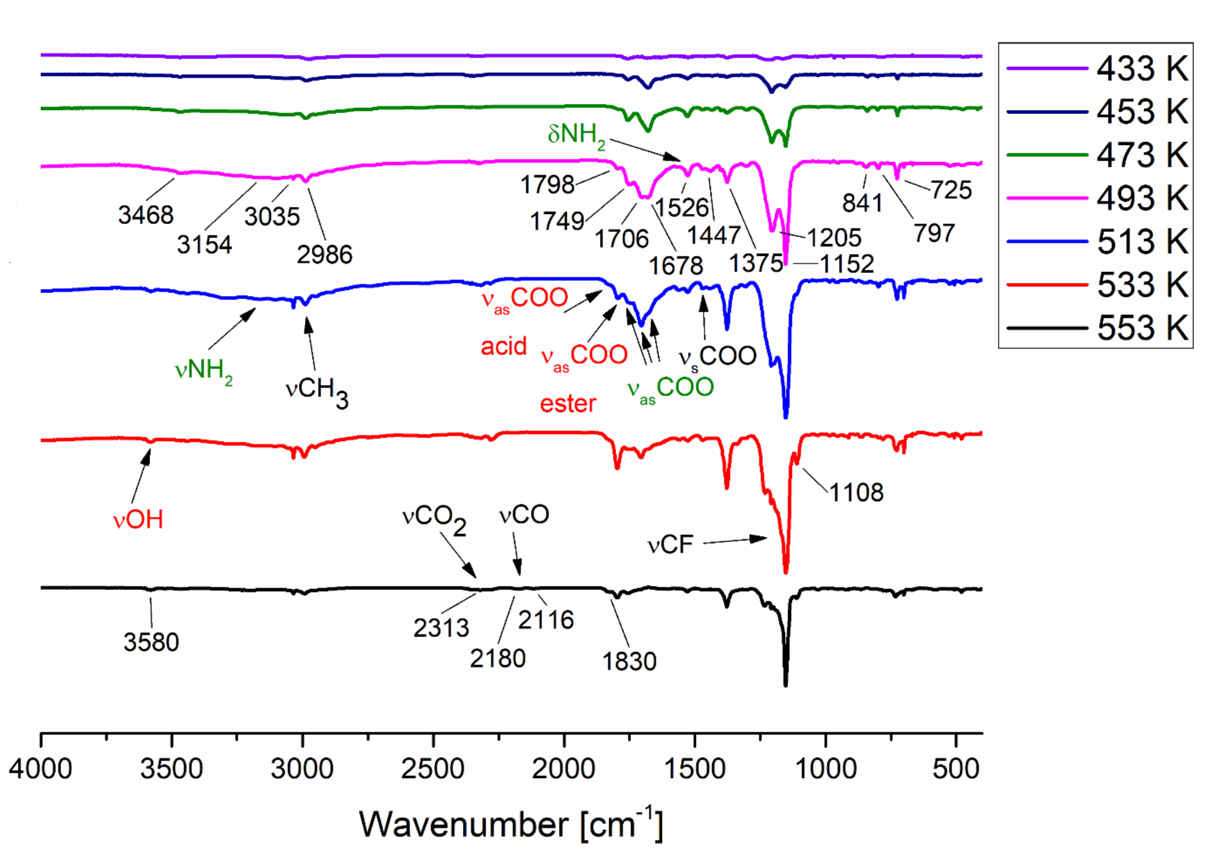

Figure S35. Temperature variable infrared spectra VT IR for the vapor formed during the [Cu2(iPrNH2)2(μ-O2CCF3)4] complex (7) heating.

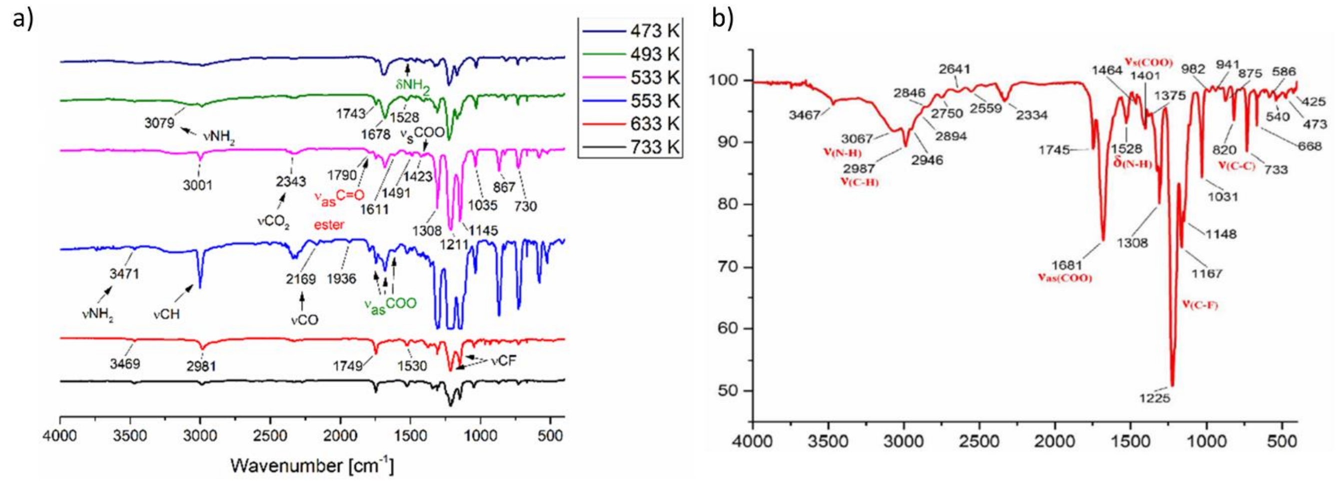

Figure S36. Temperature variable infrared spectra VT IR of vapors formed a) during the [$Cu_2(^iPrNH_2)_2(\mu\text{-}O_2CC_2F_5)_4$] complex (8) heating 433–733K and b) at temperature 493 K (red line).

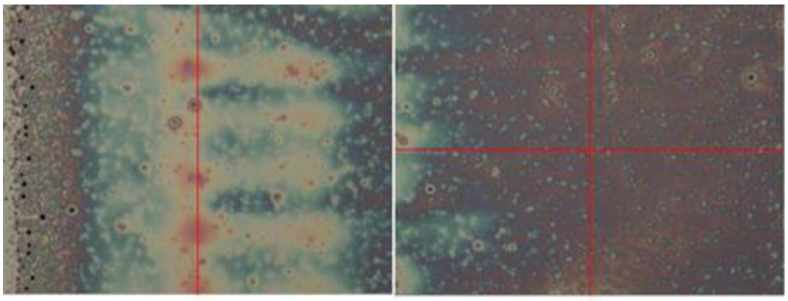

A B

Figure S37. Optical microscope images of dip-coated [$Cu_2(EtNH_2)_2(\mu\text{-}O_2CC_3F_7)_4$](3)/Si before heating, immersion time 30 s, before heating (A) the immersion line, (B) middle of the layer.

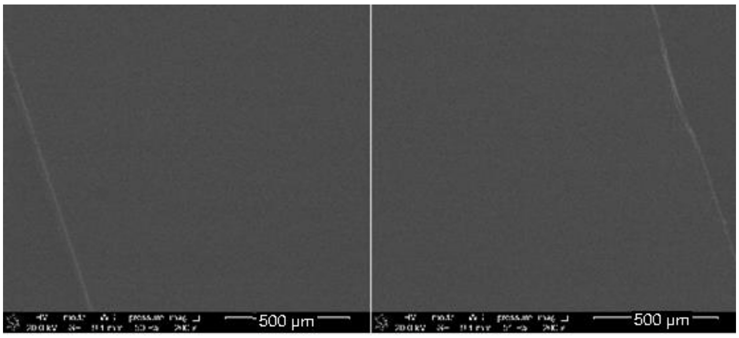

Figure S38. SEM image of dip-coated [$Cu_2(EtNH_2)_2(\mu\text{-}O_2CC_3F_7)_4$](3)/Si immersion time 30 s, Mag = 200×, before heating.

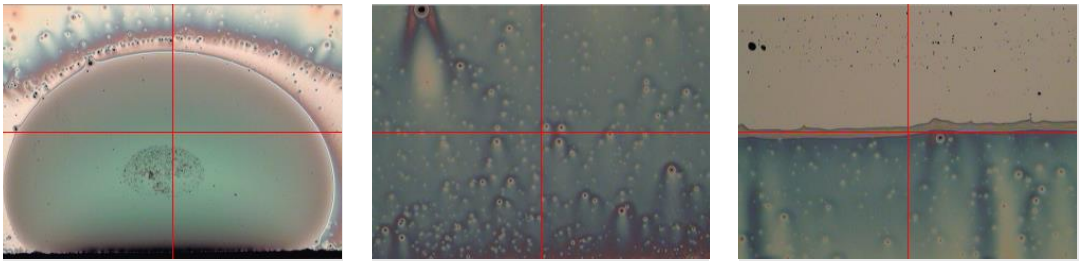

(**a**) (**b**) (**c**)

Figure S39. Optical microscope images of dip-coated $[Cu_2(EtNH_2)_2(\mu\text{-}O_2CC_3F_7)_4]$(3)/Si, before heating, immersion time 20 s, coating count 10, Mag 5x, the lower part of the substrate (A), middle of the layer (B), immersion line (C).

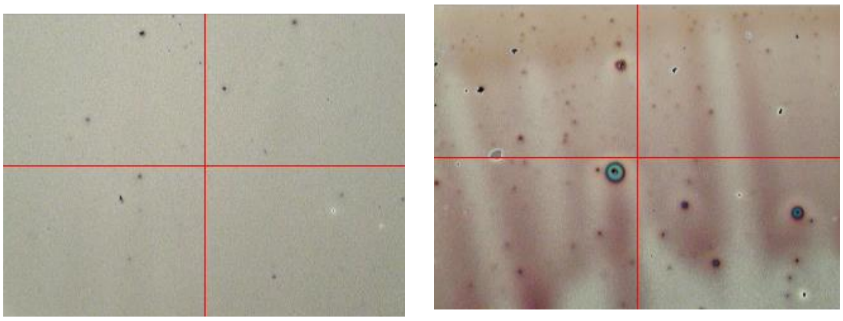

(**a**) (**b**)

Figure S40. Images from optical microscope after heating dip-coated (A) $[Cu_2(EtNH_2)_2(\mu\text{-}O_2CC_3F_7)_4]$**(3)**/Si immersion time 20 s; (B) $[Cu_2(EtNH_2)_2(\mu\text{-}O_2CC_2F_5)_4]$**(2)**/Si immersion time 20 s.
